# Length and Sequence-Selective Polymer Synthesis Templated by a Combination of Covalent and Noncovalent Base-Pairing Interactions

**DOI:** 10.1021/jacs.4c13452

**Published:** 2024-11-16

**Authors:** Federica Balduzzi, Vihanga Munasinghe, Oliver N. Evans, Agustin Lorusso Notaro Francesco, Cecilia J. Anderson, Salvatore Nigrelli, Luis Escobar, Rafel Cabot, Joseph T. Smith, Christopher A. Hunter

**Affiliations:** Yusuf Hamied Department of Chemistry, University of Cambridge, Lensfield Road, Cambridge CB2 1EW, U.K.

## Abstract

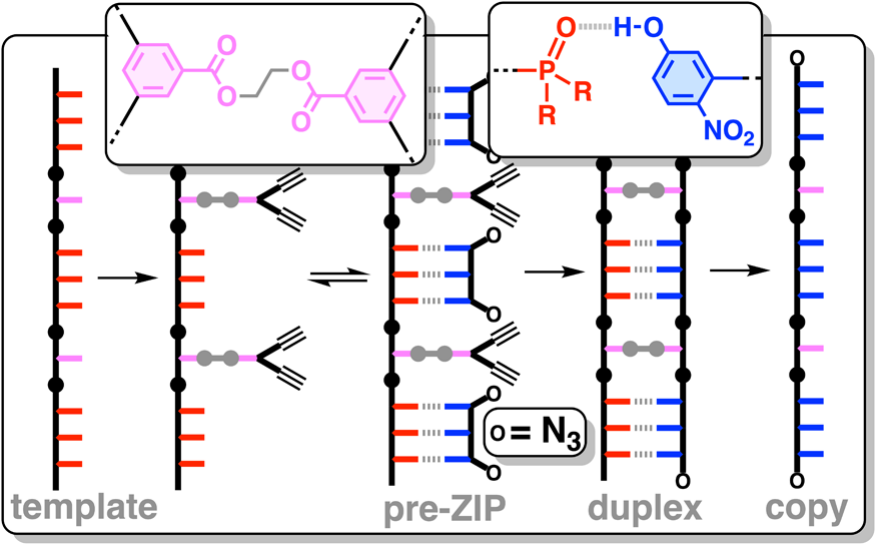

Information can be encoded and stored in sequences of monomer units organized in linear synthetic polymers. Replication of sequence information is of fundamental importance in biology; however, it represents a challenge for synthetic polymer chemistry. A combination of covalent and noncovalent base pairs has been used to achieve high-fidelity templated synthesis of synthetic polymers that encode information as a sequence of different side-chain recognition units. Dialkyne building blocks were attached to the template by using ester base pairs, and diazide building blocks were attached to the template by using H-bond base pairs. Copper-catalyzed azide–alkyne cycloaddition reactions were used to zip up the copy strand on the template, and the resulting duplex was cleaved by hydrolyzing the covalent ester base pairs. By using recognition-encoded melamine oligomers with either three phosphine oxide or three 4-nitrophenol recognition units to form the noncovalent base pairs, exceptionally high affinities of the diazides for the template were achieved, allowing the templated polymerization step to be carried out at low concentrations, which promoted on-template intramolecular reactions relative to competing intermolecular processes. Two different templates, a 7-mer and an 11-mer, were used in the three-step reaction sequence to obtain the sequence-complementary copy strands with minimal amounts of side reaction.

## Introduction

Developments in directed evolution have enabled scientists to efficiently sample biochemical sequence space, allowing the discovery of new functional molecules in a time- and resource-effective manner.^1,2^ However, this process relies on the transfer of information encoded by DNA or RNA sequences and limits the structures that can be explored to nucleic acids and peptides. Synthetic polymers that can be used to replicate sequence information would enable similar approaches to be applied to different systems.^3^ There are many examples of synthetic systems that form duplexes by exploiting base-pairing motifs that are based on H-bonds,^4−8^ metal–ligand coordination,^9,10^ salt bridges,^11,12^ or dynamic covalent chemistry.^13,14^ However, the development of template-directed synthesis mechanisms to copy sequence information in synthetic oligomers has proven more challenging.^15−17^

**Figure 1:**
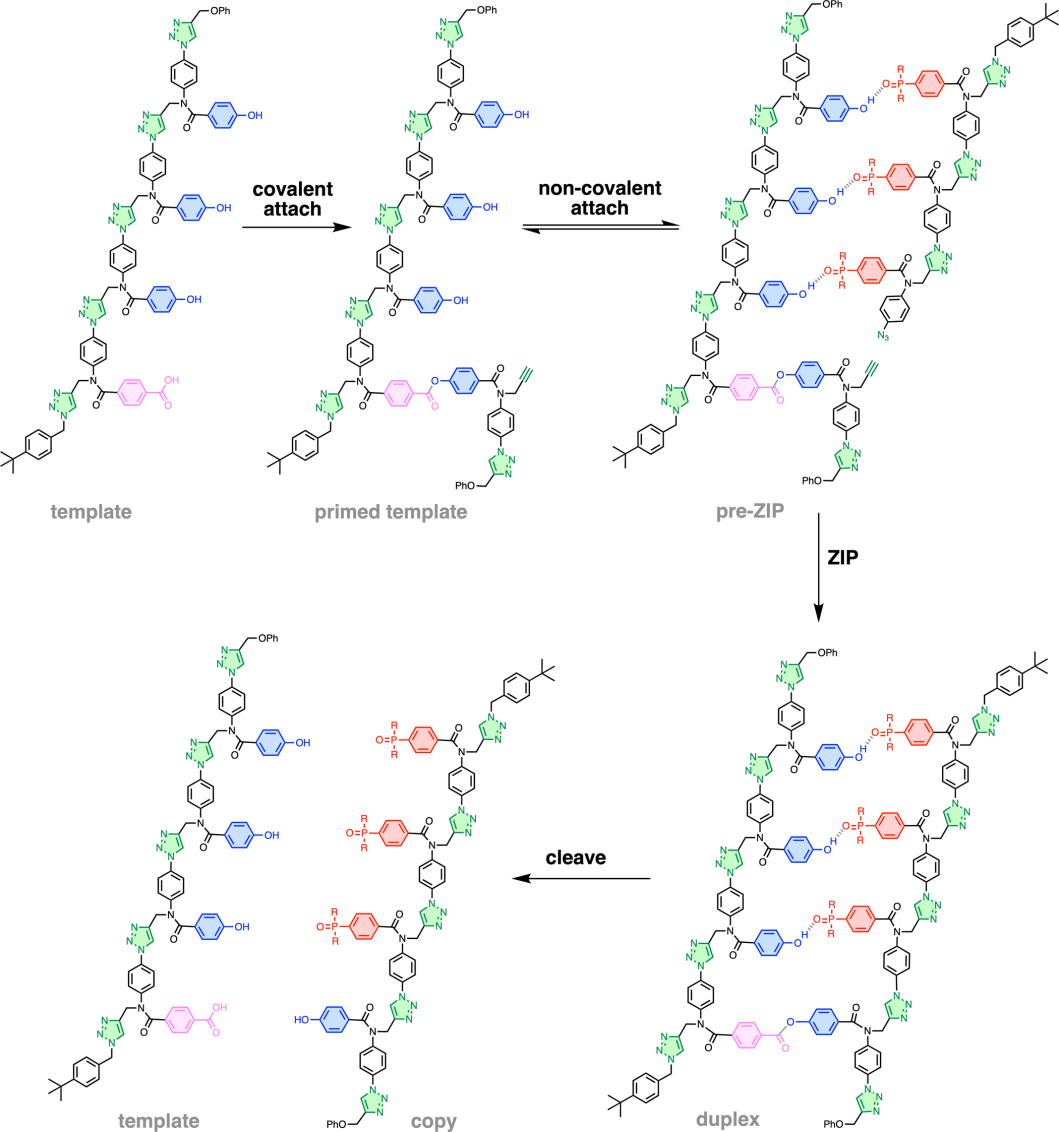
Template-directed oligomer synthesis using a covalent primer and noncovalent base-pairing interactions. A covalent base pair was used to attach the primer to the template by ester chemistry. H-bonding interactions with the phosphine oxide 3-mer gave the pre-ZIP intermediate, and an intramolecular CuAAC reaction resulted in the formation of the duplex. Hydrolysis of the ester base pair in the cleave step released the product and regenerated the template.^19^

We have previously shown that it is possible to transfer sequence information from one synthetic oligomer to another using covalent base pairing that is based on the formation and cleavage of esters of carboxylic acids and phenols.^18^ We recently showed that it should be possible to extend this approach to noncovalent base-pairing interactions based on H-bonding between phenol and phosphine oxide oligomers, as illustrated in Figure 1.^19^ A covalent primer bearing an alkyne was first attached to the template using an ester base pair, and then, an azide was attached to the template using H-bonding interactions. Figure 1 shows the binding of a phosphine oxide 3-mer to three phenols on the template via cooperative H-bonding interactions. An intramolecular copper-catalyzed azide–alkyne cycloaddition (CuAAC) reaction in the complex led to the selective formation of the duplex, as shown in Figure 1, and when the reaction was carried out in the presence of a competing azide that did not bind to the template, no incorporation of competing azide was observed. Finally, hydrolysis of the ester base pair was used to cleave the product from the template.

**Figure 2:**
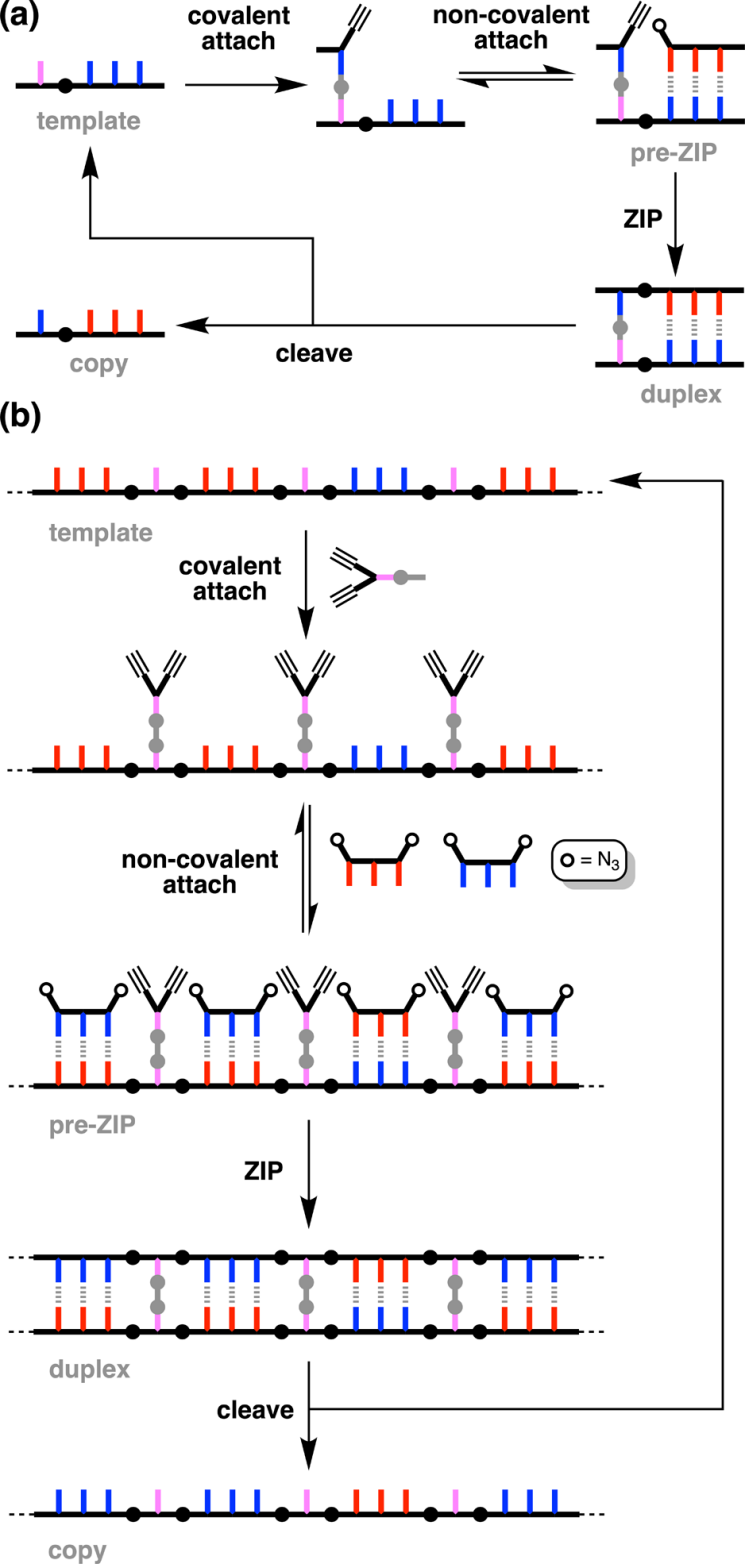
Schematic representations of the (a) ligation process in Figure 1 and (b) generalization to a mixed sequence polymer. Dialkynes are covalently bonded to the template via ester base pairs (gray circles). A diol linker (gray) is used to connect two carboxylic acids (pink), so that a carboxylic acid on the template is directly copied as a carboxylic acid on the copy strand. Diazides are noncovalently attached to the template via H-bond base pairs between phenols (blue) and phosphine oxides (red). The ZIP step represents CuAAC polymerization of the two building blocks. The cleave step represents hydrolysis of the diester linkers, regenerating the template and liberating the copy strand.

This proof of principle experiment, which corresponds to the ligation of two components, is illustrated schematically in Figure 2a. Figure 2b shows how this approach could be generalized to template-directed polymerization of dialkynes and diazides, providing a mechanism for the transfer of sequence information from the template to the copy in the replication of synthetic polymers. The steps in the replication cycle in Figure 2b are precisely the same as those in Figure 2a. The covalent base-pairing system in the polymerization process in Figure 2b uses a diester to form a covalent base pair between two carboxylic acid monomers. We have previously shown that this construct facilitates iterative rounds of replication because carboxylic acid recognition units on the template are directly replicated as carboxylic acids on the copy strand.^20^

A key feature of the process in Figure 2b is that the alkyne units are covalently attached to the template, which eliminates the possibility of any off-template reactions. The rate-limiting step of the CuAAC reaction is the activation of the alkyne by copper^21^; therefore, the selectivity of the templating process is governed entirely by the relative values of the effective molarity for the intramolecular reaction of the activated copper complex with an azide bound to the template and the concentration of unbound azides in solution that compete via intermolecular reactions.^22^ The copolymerization of two symmetrical building blocks illustrated in Figure 2b also solves one of the major limitations of the triazole backbone shown in Figure 1. The alkyne–azide monomers used to build the template strand in Figure 1 lead to directionality in the backbone, and replication experiments using this system produced product duplexes that have both parallel and antiparallel backbones. This backbone directionality compromises the fidelity of template-directed synthesis of longer polymers because two chains growing in different directions on the same template cannot connect. The use of dialkynes and diazides in Figure 2b leads to a polymer with a symmetrical backbone and no directionality issues.

**Figure 3:**
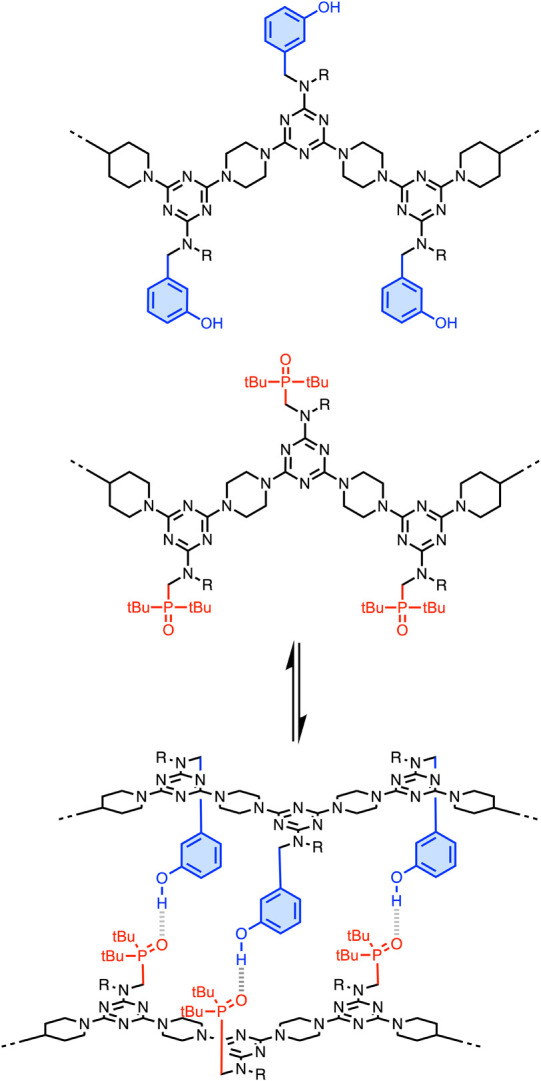
REMOs form duplexes via noncovalent base-pairing interactions between phenol and phosphine oxide side chains. R is a solubilizing group.

**Figure 4:**
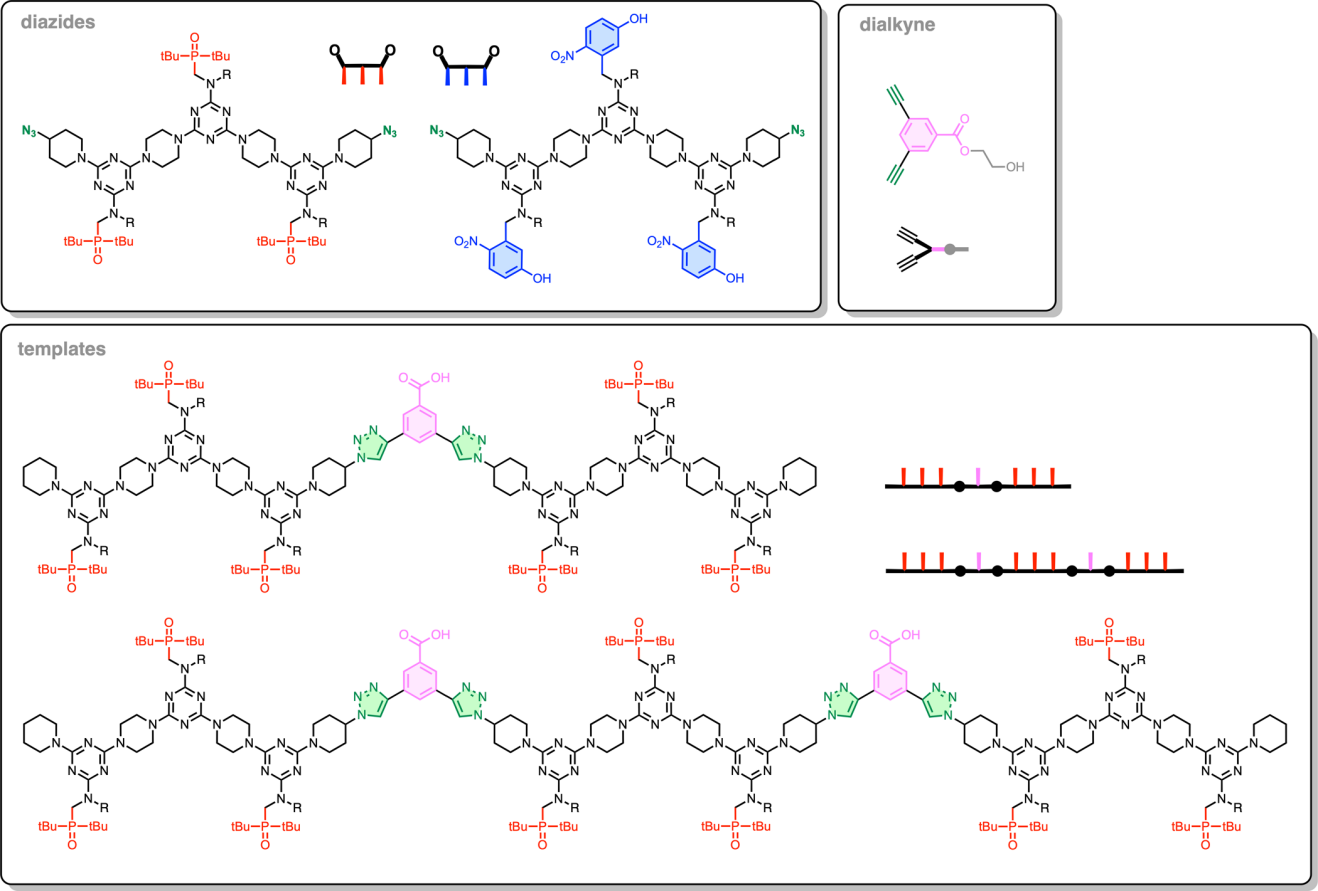
Structures of the dialkynes, diazides, and templates used to realize the templating process shown in Figure 2b.

Here, we describe the sequence-selective synthesis of synthetic polymers using the templating strategy, as outlined in Figure 2b. We use the recognition-encoded melamine oligomer (REMO) architecture illustrated in Figure 3 for the noncovalent base-pairing interactions. The REMO backbone is assembled using S_N_Ar reactions between dichlorotriazines and piperazine, and a range of different side chains can be readily incorporated as recognition units.^23^Figure 3 shows the H-bonded duplex that is formed between oligomers equipped with complementary phenol and phosphine oxide side chains.^24^ The templating experiments illustrated in Figure 1 revealed that the binding affinity of the noncovalent component plays a major role in determining the efficiency of the copying process. When an azide with one phosphine oxide unit was used, no template effect was observed, but increasing the number of phosphine oxide units increased the number of H-bonding interactions between the azide and the template, and the yield of the templated product increased dramatically.^25^ We therefore opted to use REMO 3-mers equipped with terminal azides as the noncovalent building blocks for the polymerization reaction, as shown in Figure 2b. In order to further increase the binding affinity for the template, the phenol recognition units shown in Figure 3 were replaced with 4-nitrophenols, which are much better H-bond donors.^26^ The experiments described below demonstrate successful sequence-selective templated synthesis of polymers with seven and 11 recognition units using the components shown in Figure 4.

## Results and Discussion

### Synthesis

**Scheme 1:**
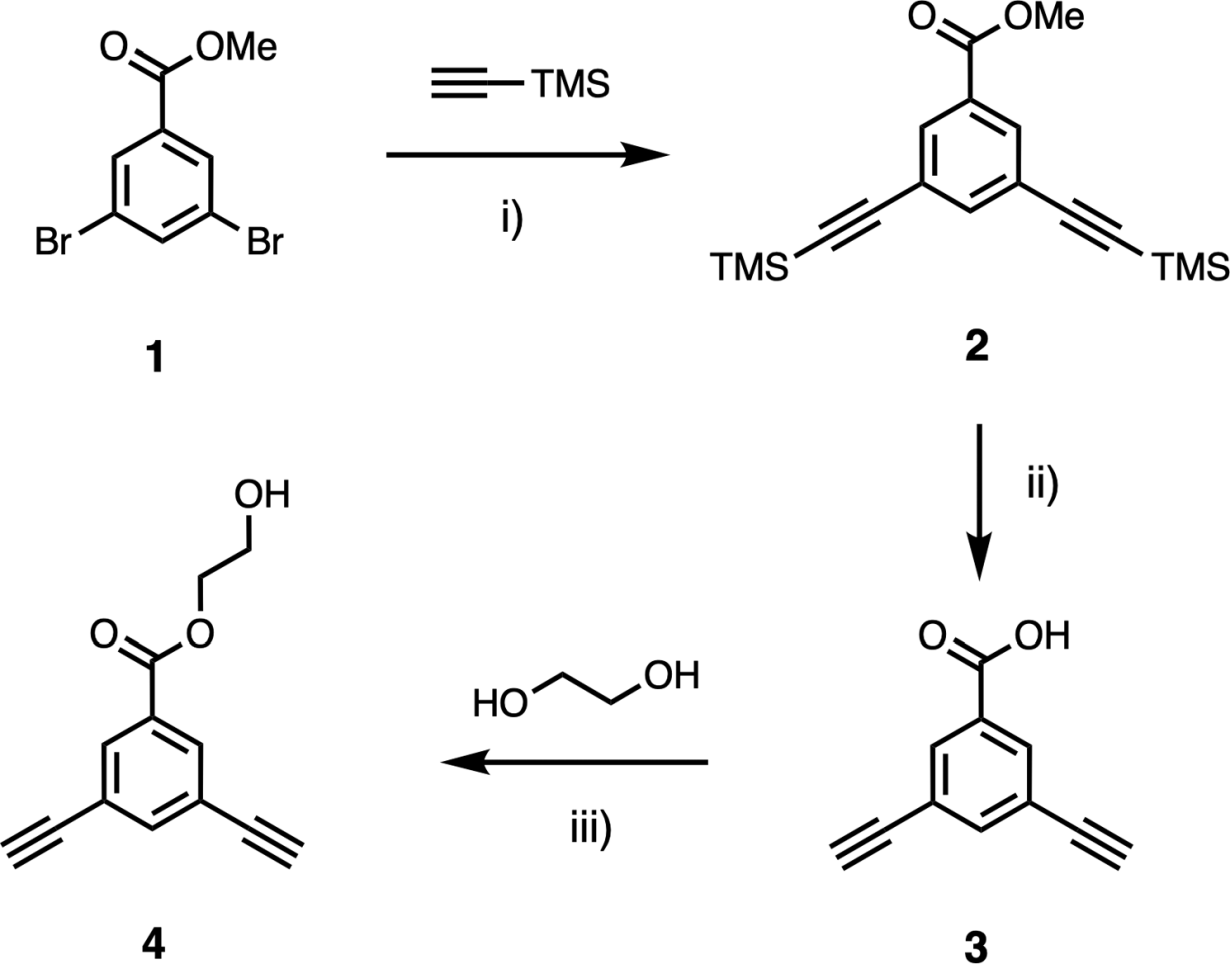
Synthesis of Dialkyne **4** (i) Pd(PPh_3_)_4_, CuI, NEt_3_, toluene, 90°C, 16 h, 45%; (ii) KOH, MeOH:THF 3:1, r.t., 16 h, 80%; and (iii) EDC, DMAP, THF, r.t., 16 h, 88%.

**Scheme 2:**
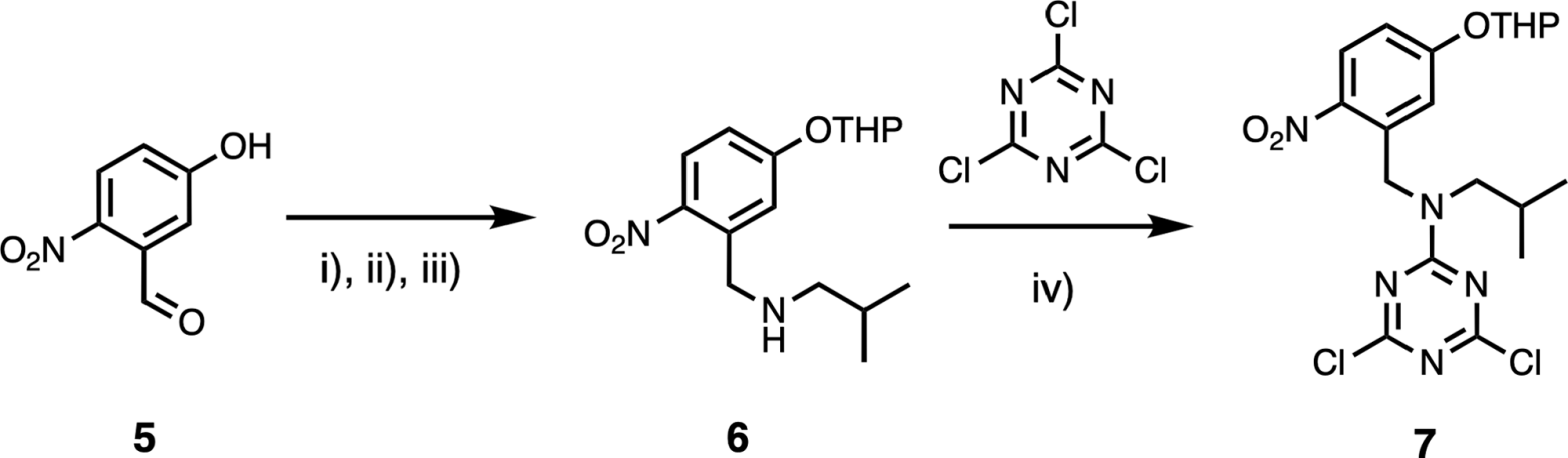
Synthesis of 4-Nitrophenol Dichlorotriazine **7** (i) 3,4-Dihydro-2H-pyran, *p*-toluenesulfonic acid, DCM, r.t., 20 h; (ii) isobutylamine, DCM, r.t., 16 h; (iii) NaBH_4_, MeOH, r.t., 3 h, 75% over 3 steps; and (iv) DIPEA, THF, −78°C, 2 h, 86%.

**Scheme 3:**
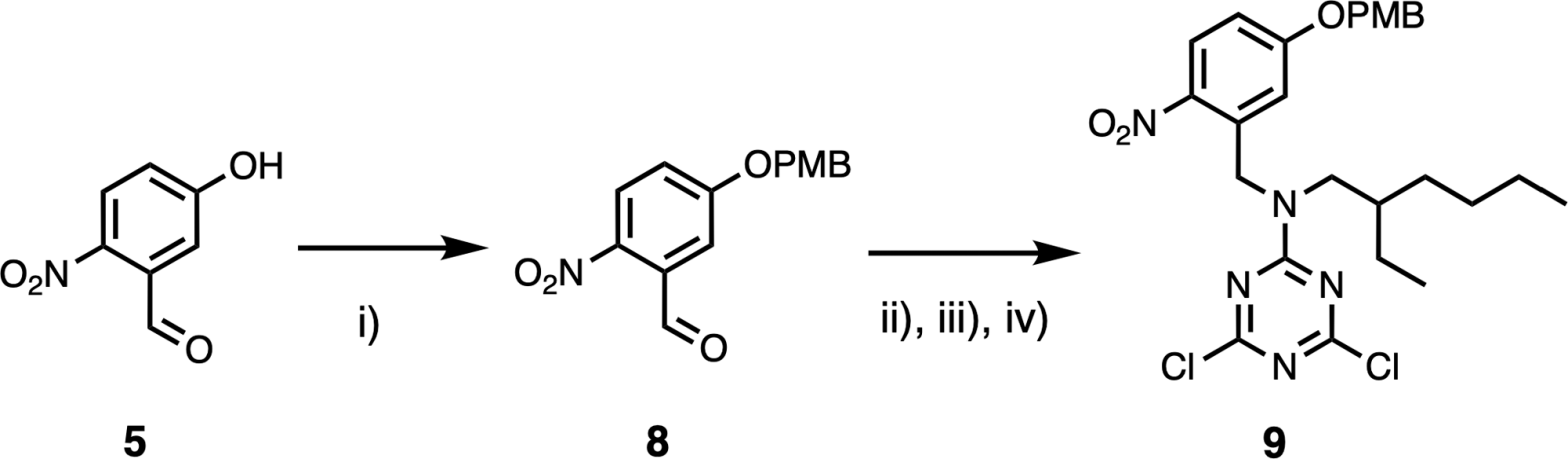
Synthesis of 4-Nitrophenol Dichlorotriazine **9** (i) PMB-Cl, K_2_CO_3_, DMF, r.t., 20 h, 85%; (ii) 2-ethyl-1-hexylamine, MeOH:CH_2_Cl_2_ 7:3, r.t., 4 h; (iii) NaBH_4_, MeOH:CH_2_Cl_2_ 7:3, r.t., 24 h; and (iv) cyanuric chloride, DIPEA, THF, −78°C, 1 h, 76% over 3 steps.

**Scheme 4:**
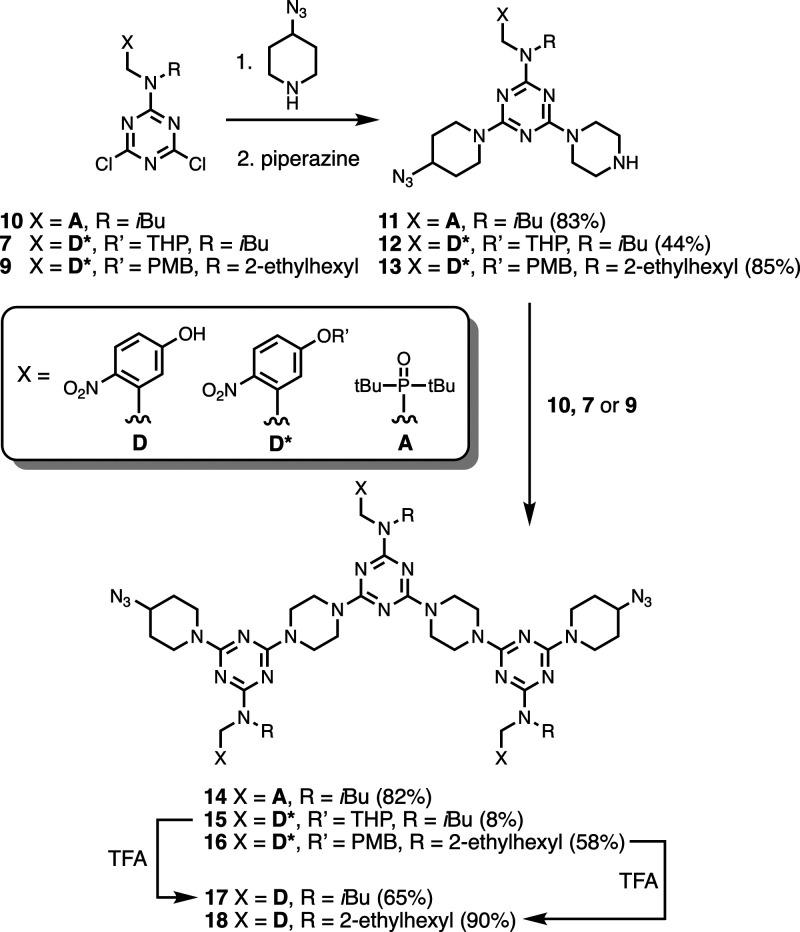
Synthesis of Diazides **zAAAz** and **zDDDz**

**Scheme 5:**
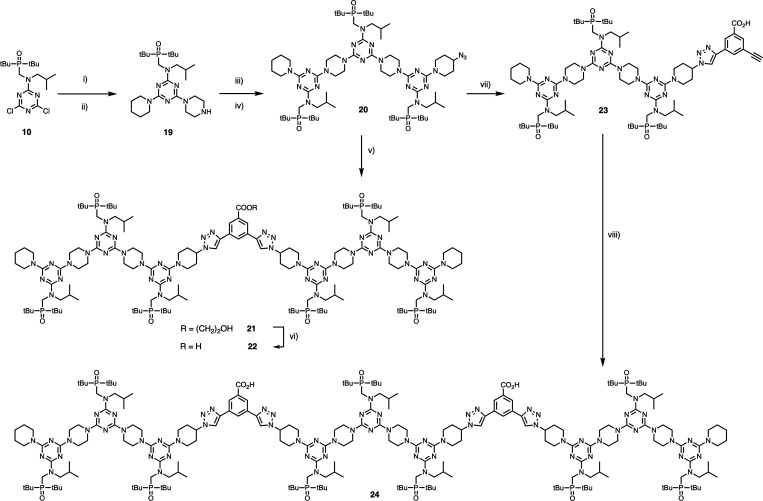
Synthesis of 7-mer Template **pAAA-C-AAAp** (**22**) and 11-mer template **pAAA-C-AAA-C-AAAp** (**24**) (i) Piperidine, DIPEA, THF, 0°C, 2 h; (ii) piperazine, THF, 60°C, 16 h, 95% over 2 steps; (iii) **10**, DIPEA, THF, 0°C, 2 h; (iv) **11**, THF, 60°C, 16 h, 91% over 2 steps; (v) **4**, [Cu(MeCN)_4_]PF_6_, TBTA, THF, r.t., 16 h, 48%; (vi) LiOH, THF:H_2_O:MeOH 5:2:2, r.t., 4 h; (vii) **3**, [Cu(MeCN)_4_]PF_6_, TBTA, THF, r.t., 16h, 32%; and (viii) **14**, [Cu(MeCN)_4_]PF_6_, TBTA, THF, r.t., 16 h.

Dialkyne **4** was synthesized from methyl 3,5-dibromobenzoate in a straightforward manner in three steps (Scheme 1). The key compounds used for the synthesis of REMO are dichlorotriazines equipped with recognition units. The phosphine oxide dichlorotriazine **10** was prepared from cyanuric chloride as described previously,^24^ and Schemes 2 and 3 show the synthesis of the two 4-nitrophenol dichlorotriazines **7** and **9**, which have different alkyl-solubilizing groups and -protecting groups. The dichlorotriazines were used in sequential S_N_Ar reactions to make two different types of diazides, **zAAAz** and **zDDDz** (Scheme 4). We use the following conventions for writing the sequence of an oligomer: **A** and **D** for H-bond acceptor and donor side chains, respectively, **C** for a carboxylic acid side chain, and **z** or **p** for terminal azide or piperidine units, respectively.^27^ Sequential S_N_Ar reactions were used to prepare a phosphine oxide 3-mer with only one azide group **pAAAz**. This compound was used in a CuAAC reaction with dialkyne **4** to obtain the 7-mer template **pAAA-C-AAAp** after hydrolysis of the ester (Scheme 5). Two sequential CuAAC reactions were used to prepare the 11-mer template **pAAA-C-AAA-C-AAAp** from **pAAAz**, **zAAAz**, and dialkyne **3** (Scheme 5).

### Binding Studies

**Figure 5:**
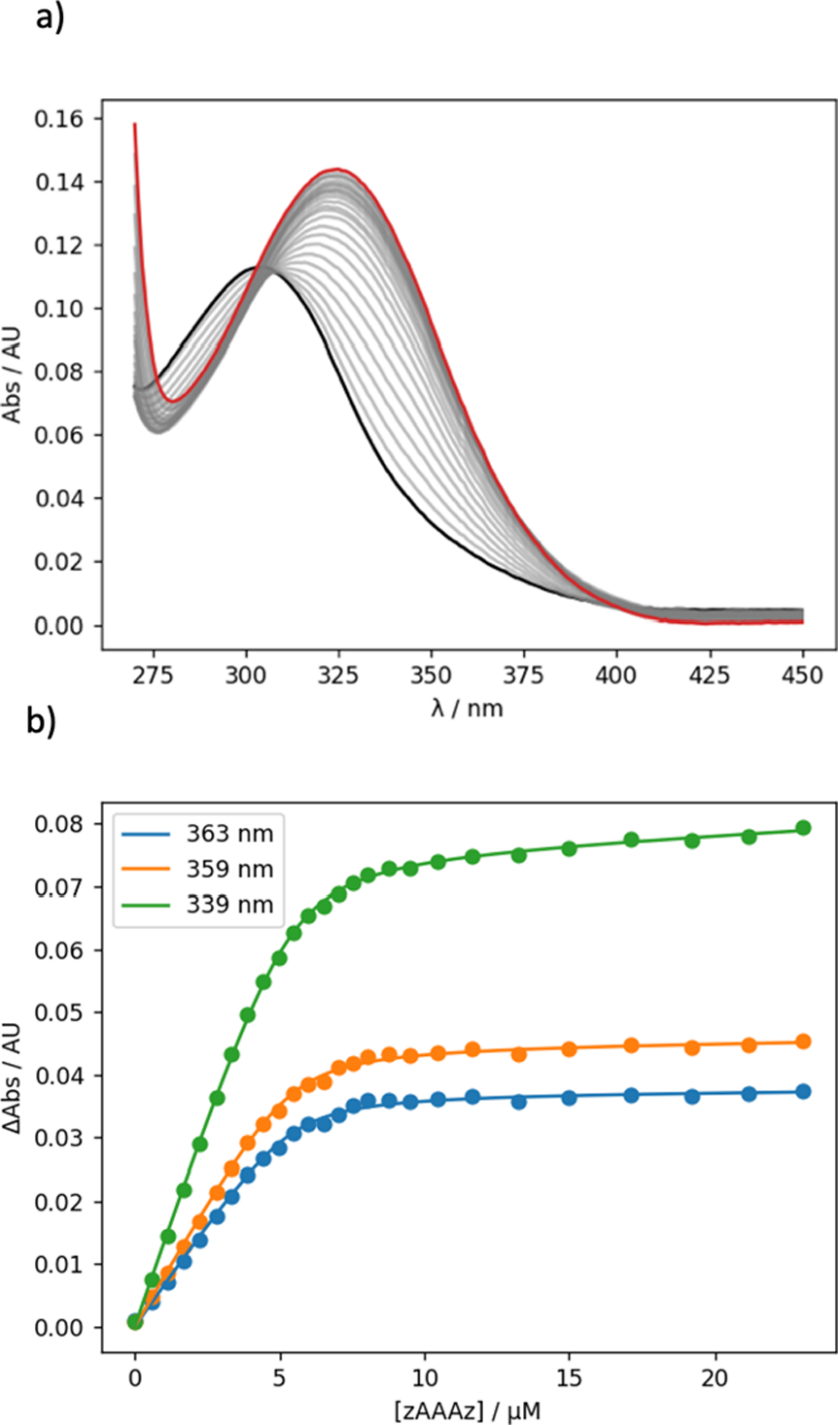
UV–vis absorption titration of **zAAAz** into **zDDDz** (5 μM) in dichloromethane at 298 K. (a) Overlay of the UV/vis absorption spectra. The initial spectrum is shown in black, and the final spectrum in red. (b) Points are the experimental measurements at selected wavelengths, and the lines are the best fit to a 1:1 binding isotherm that allows for the absorption of the guest (*K* = 4.7 × 10^6^ M^–1^).

In order to establish the best concentration regime for carrying out the templating reaction, the association constant for the formation of a duplex between complementary REMO 3-mers was measured by UV–vis absorption titrations. Figure 5a shows UV–vis absorption spectra for the addition of **zAAAz** to **zDDDz** in dichloromethane. The 4-nitrophenol units give rise to a strong absorption band at 304 nm in the UV–vis spectrum of **zDDDz**, and when **zAAAz** was added, a new band appeared at 324 nm. A large increase in the wavelength of the absorption maximum is characteristic of the formation of H-bonds with the 4-nitrophenol recognition units. The best fit to a 1:1 binding isotherm allowing for the contribution due to the absorption of the guest gave an association constant of (4.7 ± 0.4) × 10^6^ M^–1^ in dichloromethane. The corresponding association constant for the formation of a single H-bond between the 4-nitrophenol and phosphine oxide 1-mers, **pDp** and **pAp**, is 3 orders of magnitude lower in dichloromethane (*K* = (1.5 ± 0.2) × 10^3^ M^–1^, see the SI for details), which confirms that the **zAAAz**•**zDDDz** duplex is fully assembled with three cooperative H-bonding interactions at concentrations greater than 10 μM in dichloromethane.

### Template-Directed Synthesis of a 7-mer

**Figure 6:**
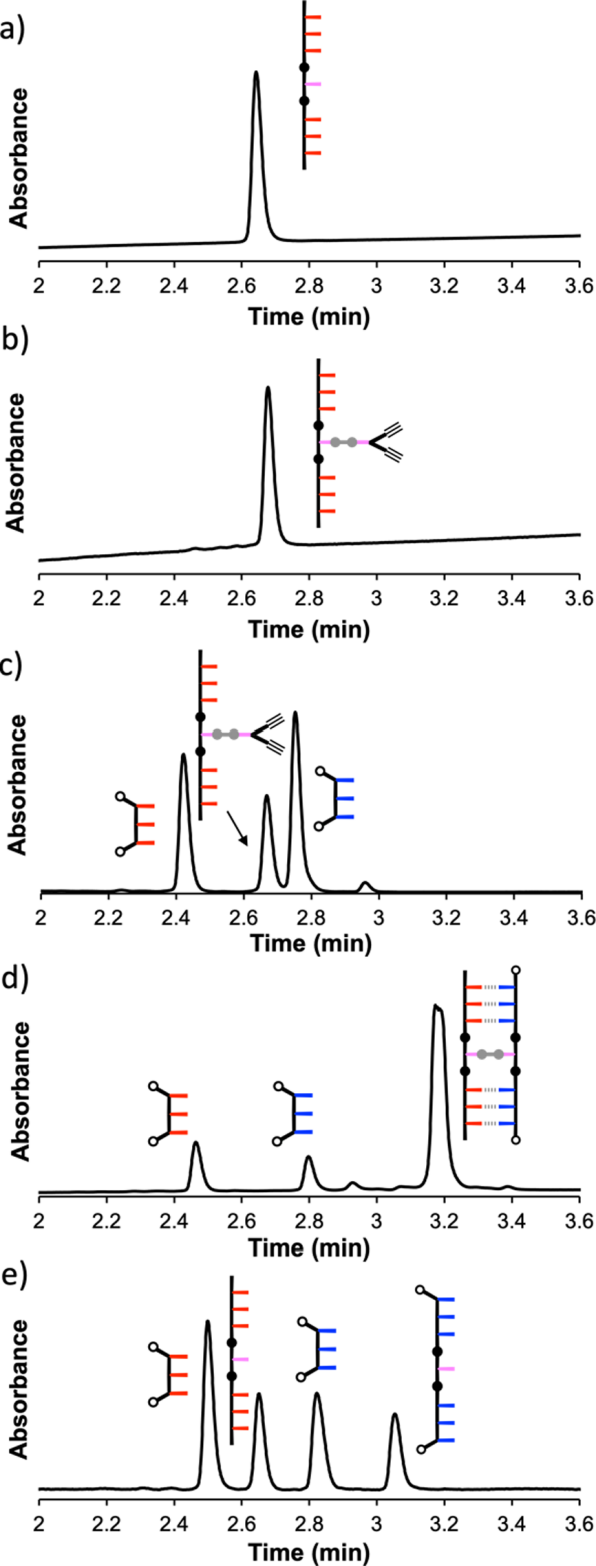
UPLC traces of the crude reaction mixtures obtained from polymerization using the 7-mer template **pAAA-C-AAAp**: (a) starting template; (b) attachment of the dialkyne; (c) mixture of the esterified template (10 μM), **14** (40 μM), and **17** (40 μM) before addition of the copper catalyst; (d) product mixture obtained after the CuAAC reaction (40 μM [Cu(CH_3_CN)_4_]PF_6_, 40 μM TBTA, DCM, r.t., 48 h); and (e) product mixture obtained after hydrolysis with LiOH in THF/H_2_O. UPLC conditions: C4 column at 40 °C using gradient of 30–100% of THF/formic acid (0.1%) in water/formic acid (0.1%) over 4 min and then 100% THF/formic acid (0.1%) over 2 min. The UV–vis absorbance at 254 nm is plotted.

**Figure 7:**
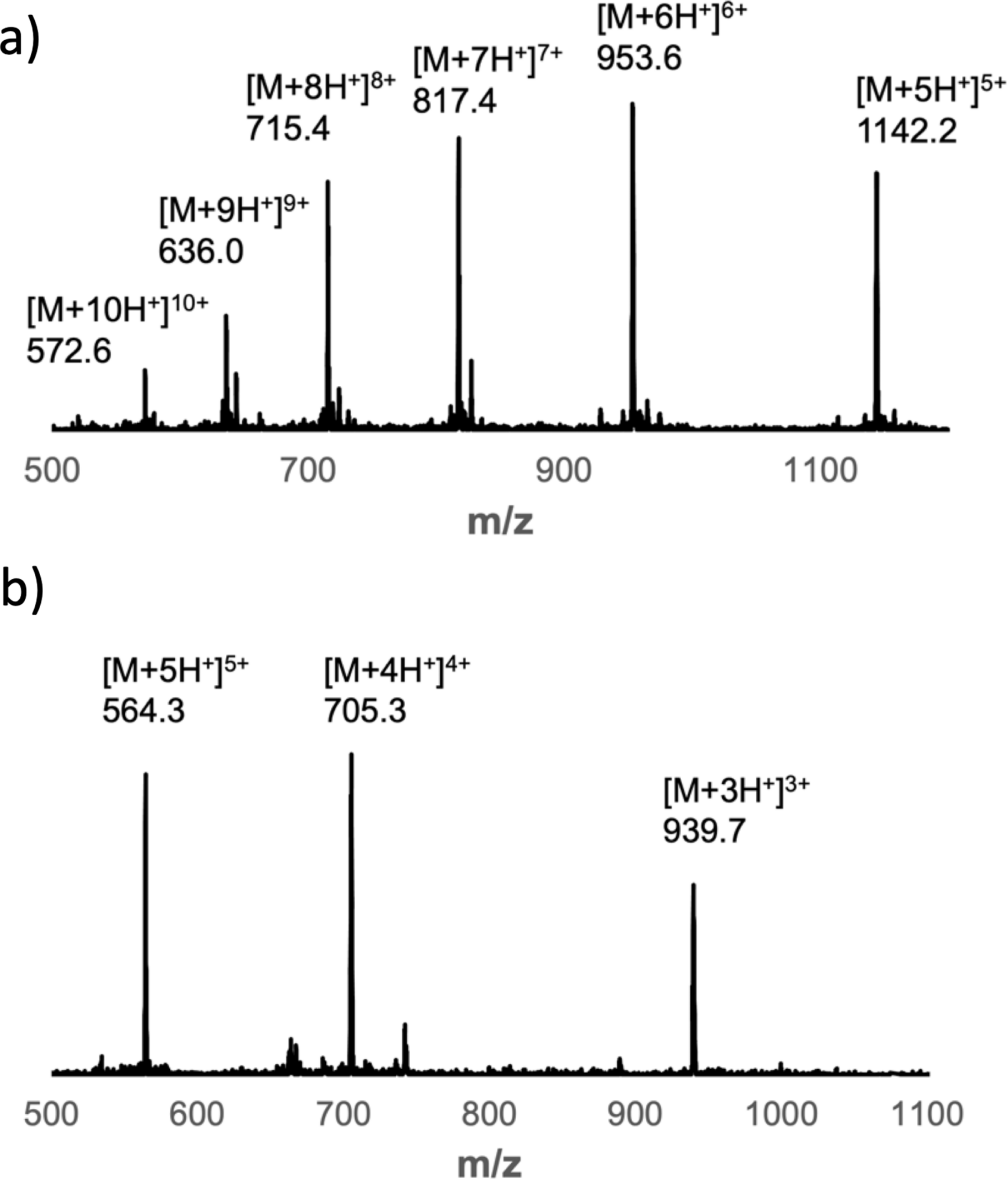
(a) ESI-MS of the UPLC peak due to the 7-mer duplex product in Figure 6d. Calculated mass (ESI^+^): 1142.8 [M+5H]^5+^, 952.9 [M+6H]^6+^, 816.9 [M+7H]^7+^, 715.0 [M+8H^+^]^8+^, 635.8 [M+9H^+^]^9+^, 572.3 [M+10H^+^]^10+^. (b) ESI-MS of the UPLC peak due to the 7-mer copy strand, **zDDD-C-DDDz** in Figure 6e. Calculated mass (ESI^+^): 939.1 [M+3H]^3+^, 704.6 [M+4H]^4+^, and 563.9 [M+5H]^5+^.

The 7-mer template **pAAA-C-AAAp** was used in the reaction sequence, as shown in Figure 2b. The first step is the covalent attachment of the dialkyne to the template via the formation of an ester base pair, which proceeded cleanly (Figure 6a,b). An excess of the two diazides, **zAAAz** and **zDDDz**, was added to a 10 μM solution of the esterified template in dichloromethane to form the pre-ZIP intermediate, and the addition of [Cu(CH_3_CN)_4_]PF_6_ and TBTA initiated the CuAAC reaction (the ZIP step of Figure 2b). Figure 6c,d shows the UPLC traces of the reaction mixture before and after the CuAAC reaction. The crude product mixture contains the two diazide starting materials, none of the template starting materials, and a new peak with a retention time of 3.2 min in the UPLC trace. Figure 7a shows the mass spectrum corresponding to this peak, which indicates that the product is the duplex formed by the reaction of the template with two equiv of **zDDDz**. No incorporation of competing diazide **zAAAz** was detected. The sequence selectivity of this process indicates that the product is formed via a templated pathway involving intramolecular reactions between the alkynes on the covalent ester base pair with **zDDDz** bound to complementary **AAA** sites on the template. The other diazide **zAAAz** does not have recognition units that would allow it to bind to the template; hence, the only pathway for the reaction of an activated copper alkyne complex on the template with this compound would be an intermolecular process, which is clearly much slower than the templated intramolecular process. The crude product mixture from the ZIP step was then subjected to ester hydrolysis using lithium hydroxide to cleave the copy strand from the template. The UPLC trace of the crude product mixture shows that this reaction also proceeded cleanly to give four different oligomers, the unreacted diazides, **zAAAz** and **zDDDz**, the 7-mer template, **pAAA-C-AAAp**, and the 7-mer copy strand, **zDDD-C-DDDz** (Figure 6e). Figure 7b shows the mass spectrum corresponding to the new peak in the UPLC trace, which confirms that it contains only the copy strand, **zDDD-C-DDDz**.

Although the heights of the peaks in the UPLC traces in Figure 6 are related to the relative amounts of different species that are present, the peak widths and extinction coefficients differ between the species. For example, **zAAAz**, **zDDDz**, and **pAAA-C-AAAp** were mixed in a 4:4:1 ratio in Figure 6c. The peaks due to **zDDDz** and **zAAAz** are similar in intensity, but the peak due to **zAAAz** is only double the intensity of the peak due to **pAAA-C-AAAp**. The intensity of the **pAAA-C-AAAp** peak reflects the fact that it has twice as many chromophores as **zAAAz**. In Figure 6e, the peaks due to **zDDDz**, **pAAA-C-AAAp**, and **zDDD-C-DDDz** all have similar intensities, and the peak due to **zAAAz** is twice as intense. The 7-mers have roughly double the extinction coefficient of the 3-mers, and after the ZIP reaction, there are four equiv of **zAAAz** left; hence, this peak is twice as intense as the 7-mer peaks. Only two equivalents of **zDDDz** remain because the other two equivalents are used to make **zDDD-C-DDDz**; hence, these two peaks have a similar intensity. These results suggest that the extinction coefficient is roughly proportional to the length of the oligomer; hence, visual inspection of the peak intensity provides a reasonable method for gauging the relative amounts of different species present.

### Template-Directed Synthesis of an 11-mer

**Figure 8:**
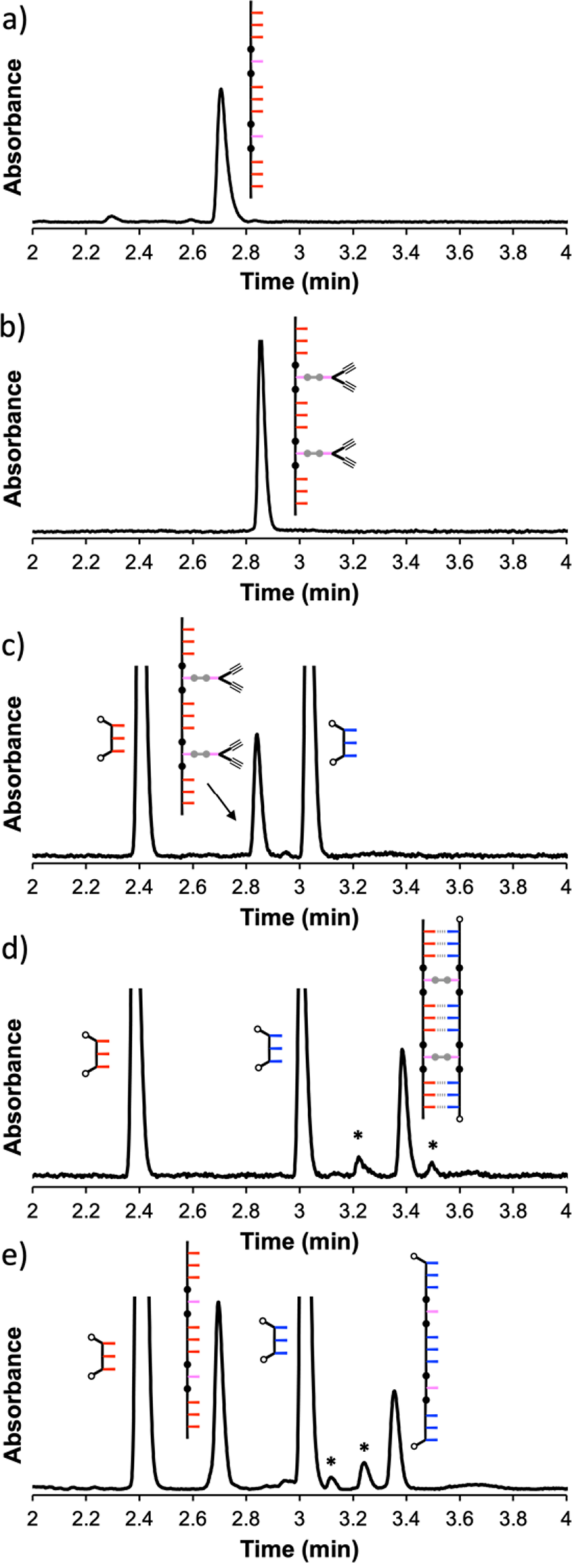
UPLC traces of the crude reaction mixtures obtained from polymerization using the 11-mer template **pAAA-C-AAA-C-AAAp**: (a) starting template; (b) attachment of the dialkynes; (c) mixture of the esterified template (5 μM), **14** (90 μM), and **18** (90 μM) before addition of the copper catalyst; (d) product mixture obtained after the CuAAC reaction (40 μM [Cu(CH_3_CN)_4_]PF_6_, 40 μM TBTA, DCM, r.t., 48 h); and (e) product mixture obtained after hydrolysis with LiOH in THF/H_2_O. Side products are marked with asterisks in panels (d) and (e). UPLC conditions: C4 column at 40 °C using gradient of 30–100% of THF/formic acid (0.1%) in water/formic acid (0.1%) over 4 min, and then 100% THF/formic acid (0.1%) over 2 min. The UV–vis absorbance at 254 nm is plotted.

**Figure 9:**
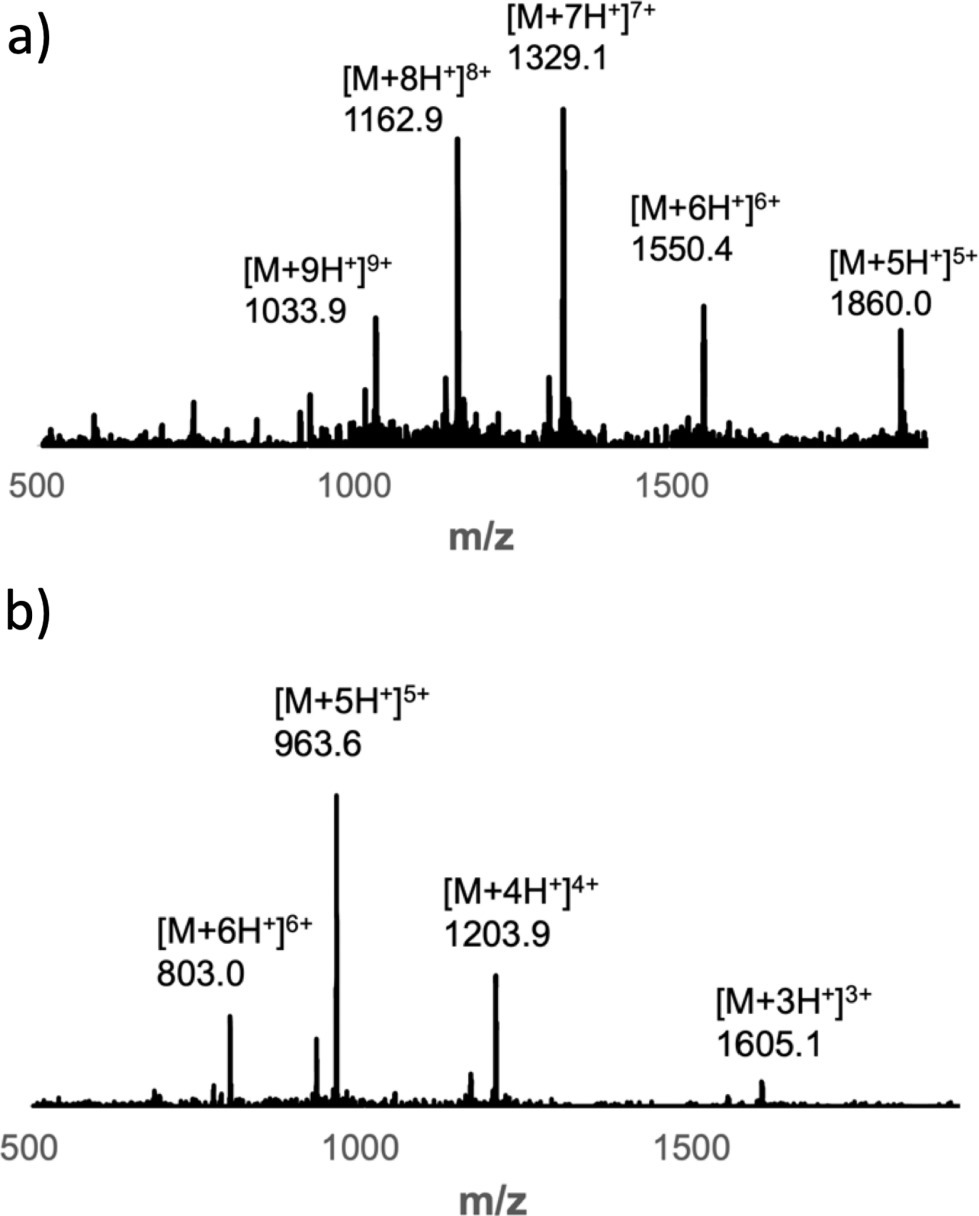
(a) ESI-MS of the UPLC peak due to the 11-mer duplex product in Figure 8d. Calculated mass (ESI^+^): 1860.1 [M+5H]^5+^, 1550.3 [M+6H]^6+^, 1328.9 [M+7H]^7+^, 1163.0 [M+8H]^8+^, 1033.8 [M+9H]^9+^. (b) ESI-MS of the UPLC peak due to the 11-mer copy strand, **zDDD-C-DDD-C-DDDz** in Figure 8e. Calculated mass (ESI^+^): 1604.9 [M+3H]^3+^, 1204.2 [M+4H]^4+^, 963.5 [M+5H]^5+^, and 802.9 [M+6H]^6+^.

The reaction sequence shown in Figure 2b was then carried out using the 11-mer template **pAAA-C-AAA-C-AAAp**. UPLC traces of the crude reaction mixtures obtained after each step of the cycle are shown in Figure 8. Again, the esterification reaction to covalently attach the dialkynes proceeded cleanly (Figure 8b). The CuAAC reaction in the ZIP step resulted in one major product (Figure 8d), and the mass spectrum in Figure 9a shows that this product is the 11-mer duplex, in which three **zDDDz** units reacted with the alkynes on the template. There are two smaller peaks indicated with asterisks in the UPLC trace in Figure 8d, and these products correspond to the incorporation of two or four **zDDDz** units (see the SI). No incorporation of the competing diazide **zAAAz** was observed, which indicates that all of the CuAAC reactions are intramolecular processes involving alkynes and azides bound to the template. Hydrolysis of the ester base pairs with lithium hydroxide cleaved the duplex, regenerating the template and releasing the copy strand (Figure 8e). Figure 9b shows the mass spectrum corresponding to the peak at 3.4 min in the UPLC trace, which confirms that the duplex has been fully hydrolyzed to give the copy strand, **zDDD-C-DDD-C-DDDz**.

**Figure 10:**
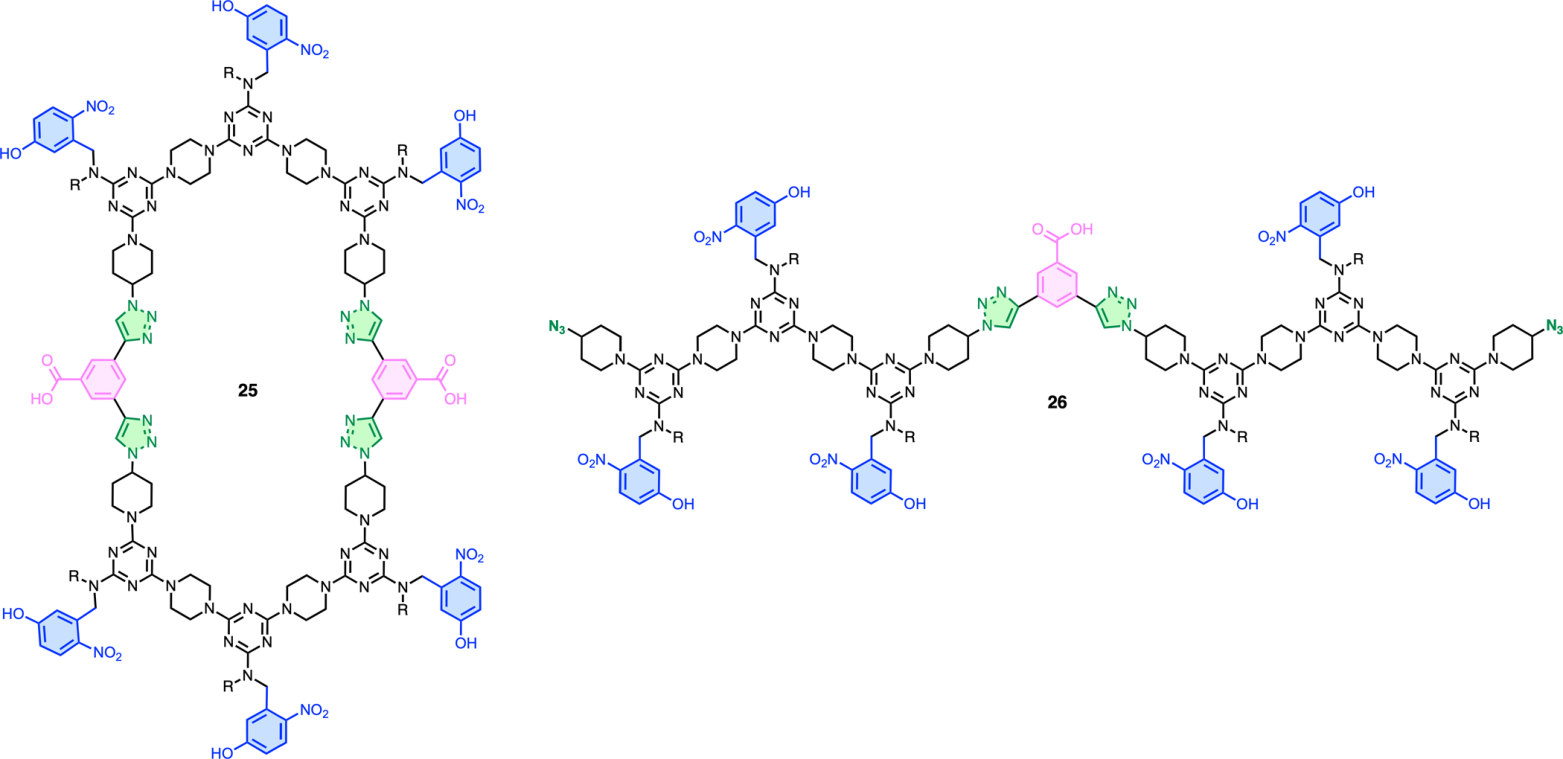
Structures of the two truncated side products observed in polymerization reactions using the 11-mer template.

**Figure 11:**
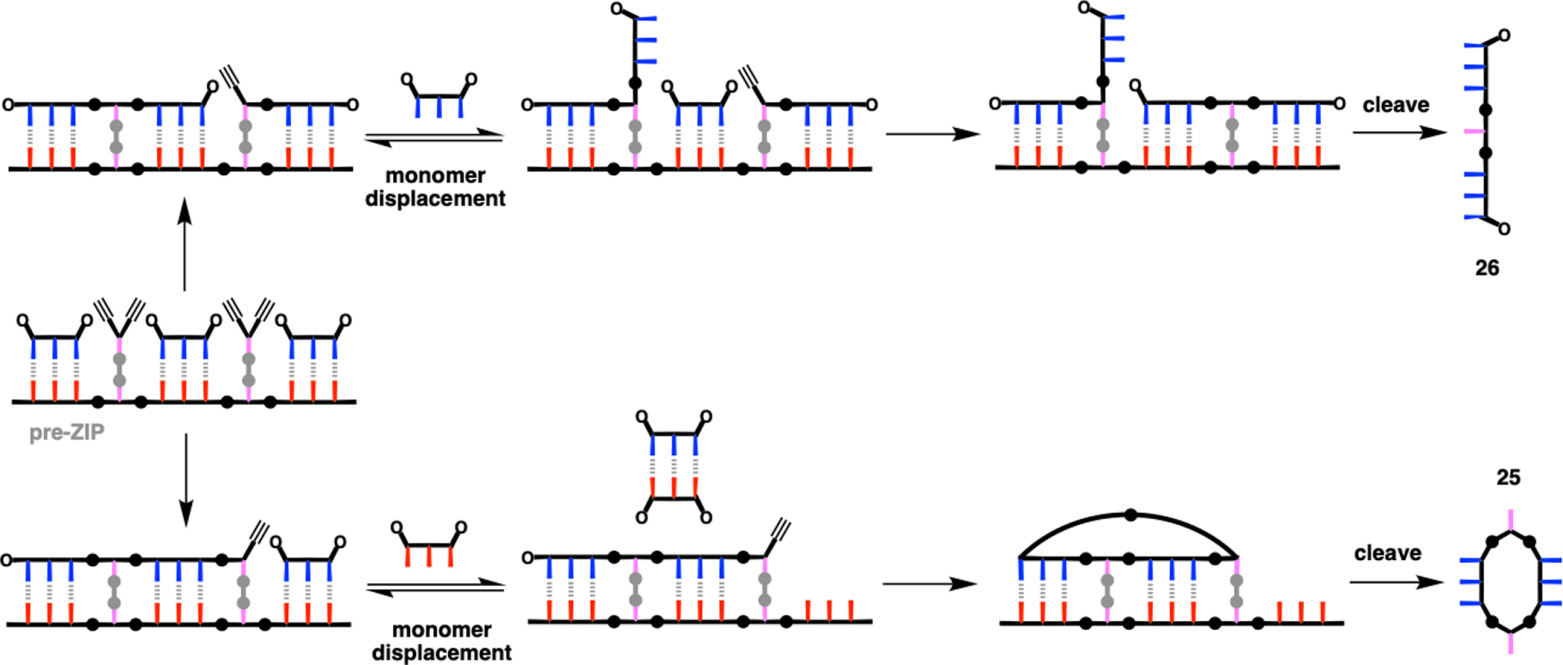
Displacement equilibria in partially zipped intermediates account for the two side products observed in polymerization reactions using the 11-mer template.

Templating with the 11-mer is more challenging than templating with the 7-mer because the formation of the duplex requires four rather than two CuAAC reactions, and the number of possible competing reactions increases correspondingly. Figure 10 shows the structures of the two truncated side products identified in the UPLC trace of the final product mixture, as shown in Figure 8e. Figure 11 shows the reaction pathways that could account for the side reactions. The mass spectra associated with the side product peaks in Figure 8d show that one compound arises from the incorporation of two equivalents of **zDDDz** and the other arises from the incorporation of four equivalents of **zDDDz**. Figure 11 illustrates how both of these side products could arise from the displacement of **DDD** units from the template by the excess of diazides present in the solution. Displacement of a partially reacted **zDDDz** from the template by another **zDDDz** would lead to the incorporation of four equivalents of **zDDDz**, and the truncated linear product **26** would be obtained after the cleave step. The removal of unreacted **zDDDz** from a partially reacted template by binding to **zAAAz** would lead to a macrocylization reaction on the template, and the truncated cyclic product **25** would be obtained after the cleave step. Neither of the processes shown in Figure 11 can affect CuAAC reactions on the 7-mer template, which is consistent with the absence of truncated copies in the product mixture shown in Figure 6e. Further evidence of the nature of the side reactions on the 11-mer template was obtained by varying the relative proportions of the two diazides present in the reaction mixture. Increasing the proportion of **zDDDz** increased the amount of truncated linear product, and increasing the proportion of **zAAAz** increased the amount of truncated cyclic product (see the SI for details), which is consistent with the two reaction pathways shown in Figure 11.

## Conclusions

**Figure 12:**
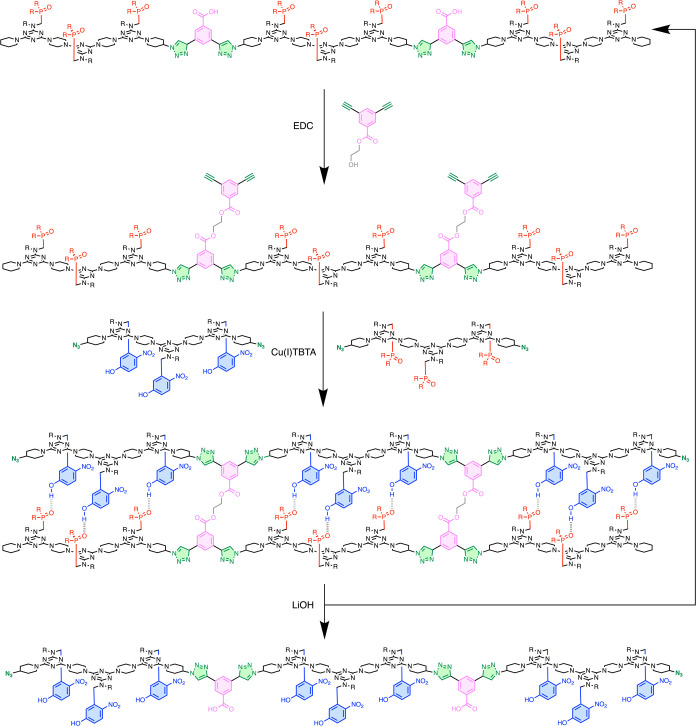
Three-step cycle for sequence-selective synthesis of an 11-mer using a complementary template.

The experiments described here show that a combination of covalent and noncovalent base-pairing interactions can be used to successfully transfer sequence information from a synthetic polymeric template to a complementary copy strand. The three-step reaction sequence is illustrated in Figure 12. Dialkynes were covalently attached to the template using ester base pairs, and diazides were noncovalently attached to the template using H-bonding interactions. CuAAC reactions were used to zip up the copy strand on the template, and the resulting duplex was cleaved by hydrolyzing the ester base pairs. By using REMOs with either three phosphine oxide or three 4-nitrophenol recognition units to form the noncovalent base pairs, exceptionally high affinities of the diazides for the template were achieved, allowing the ZIP step in Figure 2b to be carried out at low concentrations, which promoted on-template intramolecular reactions relative to competing intermolecular processes. When a 7-mer template was used, only the sequence-complementary copy strand was formed. When an 11-mer template was used, small amounts of two different side products were observed in addition to the sequence-complementary copy strand. These side products are formed via intramolecular reactions on the template and arise from competitive binding interactions with the excess diazides present in the solution. Nevertheless, the hybrid base-pair approach described here represents an attractive strategy for the development of replication processes for longer mixed sequence synthetic polymers.

As shown in Figures 2b and 12, the three-step reaction sequence regenerates the original template along with the copy strand. Both oligomers are equipped with two carboxylic acids and nine H-bond recognition sites, and both oligomers can, therefore, be used as the template in repeated rounds of a polymer replication cycle. Although it might be possible to use the crude product mixtures for iterative replication cycles, Figures 6 and 8 show that the template and copy strands can also be separated by chromatography, which would give a greater level of control. The use of noncovalent base pairs significantly improves the overall time for one cycle of replication compared with the all-covalent system reported previously.^18^ Replication using the protecting group chemistry required for the all-covalent base-pair approach is a six-step process, whereas the approach described here is a three-step process. The time taken to carry out the complete cycle shown in Figure 12 was about 4 days without optimization of any of the steps.

## Supporting Information Available

Materials and methods, detailed synthetic procedures, characterization including NMR spectra and HRMS of all compounds, UV–vis absorption titration data, UPLC and mass spectra for templating experiments (PDF)
