# Supplementary material for: Length and Sequence-Selective Polymer Synthesis Templated by a Combination of Covalent and Noncovalent Base-Pairing Interactions
Source: J Am Chem Soc. 2024 Nov 16;146(47):32837–47. doi: 10.1021/jacs.4c13452 (PMC11613443; doi:10.1021/jacs.4c13452)
Supplement: Supplementary file 1 — ja4c13452_si_001.pdf [file ja4c13452_si_001.pdf]

Length and Sequence-Selective Polymer Synthesis Templated by a Combination of Covalent and Non-Covalent Base-Pairing Interactions.

Federica Balduzzi, Vihanga Munasinghe, Oliver N. Evans, Agustin Lorusso Notaro Francesco, Cecilia J. Anderson, Salvatore Nigrelli, Luis Escobar, Rafel Cabot, Joseph T. Smith and Christopher A. Hunter*

Yusuf Hamied Department of Chemistry, University of Cambridge, Lensfield Road, Cambridge CB2 1EW, UK.

herchelsmith.orgchem@ch.cam.ac.uk

Supplementary information

1. General experimental details S2
2. Synthesis and characterization S3
3. Template experiments S58
4. Binding studies S60
5. References S64

1. General experimental details

Commercial reagents were purchased from Sigma-Aldrich, Alfa Aesar, Fisher Scientific, Fluorochem or Acros Organics and were used without further purification unless otherwise specified. All air and moisture sensitive manipulations were carried out using standard vacuum line and Schlenk techniques. Anhydrous THF and CH_2Cl_2 were obtained from a Grubbs PS-MD-5 solvent purification system. Thin layer chromatography was carried out using silica gel 60F (Merck) on glass plates and visualised using UV light (254 or 365 nm) and/or developed using potassium permanganate. Flash chromatography was carried out on an automated system (Combi*Flash*® Rf$^+$ or Combi*Flash*®Rf$^+$ Lumen) using prepacked cartridges of silica (25 µm or 50 µm PuriFlash® columns). All NMR spectroscopy was carried out on a Bruker 400 MHz DPX400, 400 MHz AVIII400, 500 MHz DCH cryoprobe, 500 MHz TCI Cryoprobe or 700 MHz TXOCryo spectrometer using the residual solvent as the internal standard. All chemical shifts (δ) are quoted in ppm and coupling constants given in Hz. UPLC analysis of samples was performed using Waters Acquity H-class UPLC coupled with a single quadrupole Waters SQD2. Three different columns were used: Acquity UPLC CSH C18 column (130 Å, 1.7 µm, 2.1 mm x 50 mm), Acquity UPLC BEH C8 column (130 Å, 1.7 µm, 2.1 mm x 50 mm) and Acquity UPLC BEH C4 column (300 Å, 1.7 µm, 2.1 mm x 50 mm). Analytical reversed-phase HPLC was performed on an Agilent HP-1100 Series HPLC system, using a XBridge® BEH C8 (2.5 µm, 4.6 x 75 mm) column. Preparative reversed-phase HPLC was performed on an Agilent HP-1100 Series preparative HPLC system, using a a XBridge® BEH C8 (5 µm, 19 x 250 mm) column. HRMS analysis was performed in an Agilent 1100 pump and autosampler with a Waters LCT Premier TOF (Time of Flight) mass spectrometer.

2. Synthesis and characterization

2.1 Synthesis of dialkyne 4

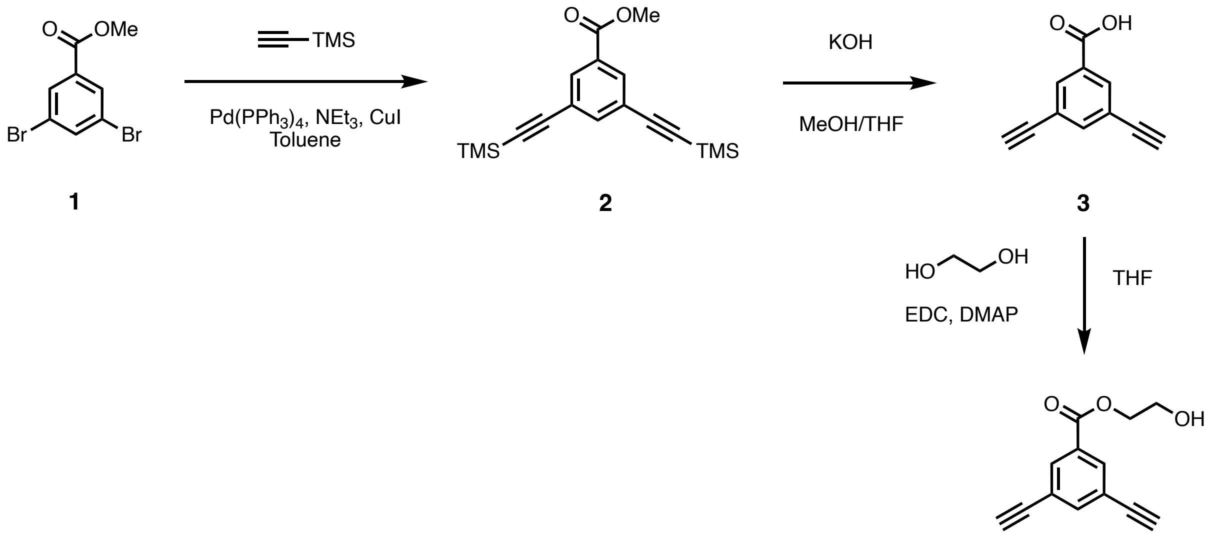

Scheme S1. Synthesis of dialkyne **4**.

Synthesis of compound 2

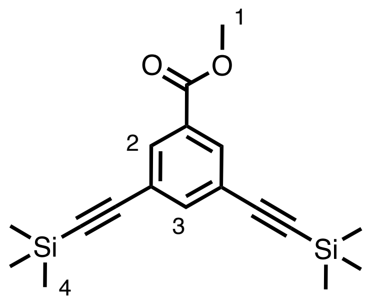

Under an inert N_2 atmosphere, methyl 3,5-dibromobenzoate (2 g, 6.8 mmol), $Pd(PPh_3)_4$ (0.82 g, 0.71 mmol) and CuI (0.14 mg, 0.71 mmol) were suspended in degassed toluene (24 mL) and degassed NEt_3 (24 mL). Trimethylsilylacetylene (2.4 mL, 17.8 mmol) was added, and the suspension was stirred at 90°C for 16 hours. The reaction mixture was filtered through Celite and washed with EtOAc. The organic phase was washed with HCl (1M, 2 x 100 mL) and concentrated under reduced pressure. The residue was purified by column chromatography (EtOAc:petroleum ether 40-60°C, 1:9) and the resulting solid was washed with MeOH. Compound **2** was obtained as a white solid (1 g, 45%).

^{1}H NMR (400 MHz, $CDCl_3$) δ 8.04 (d, *J* = 1.6 Hz, 2H, C2H), 7.72 (t, *J* = 1.6 Hz, 1H, C3H), 3.92 (s, 3H, C1H_3), 0.25 (s, 18H, C4H_3). *Data in agreement with literature.*[1]

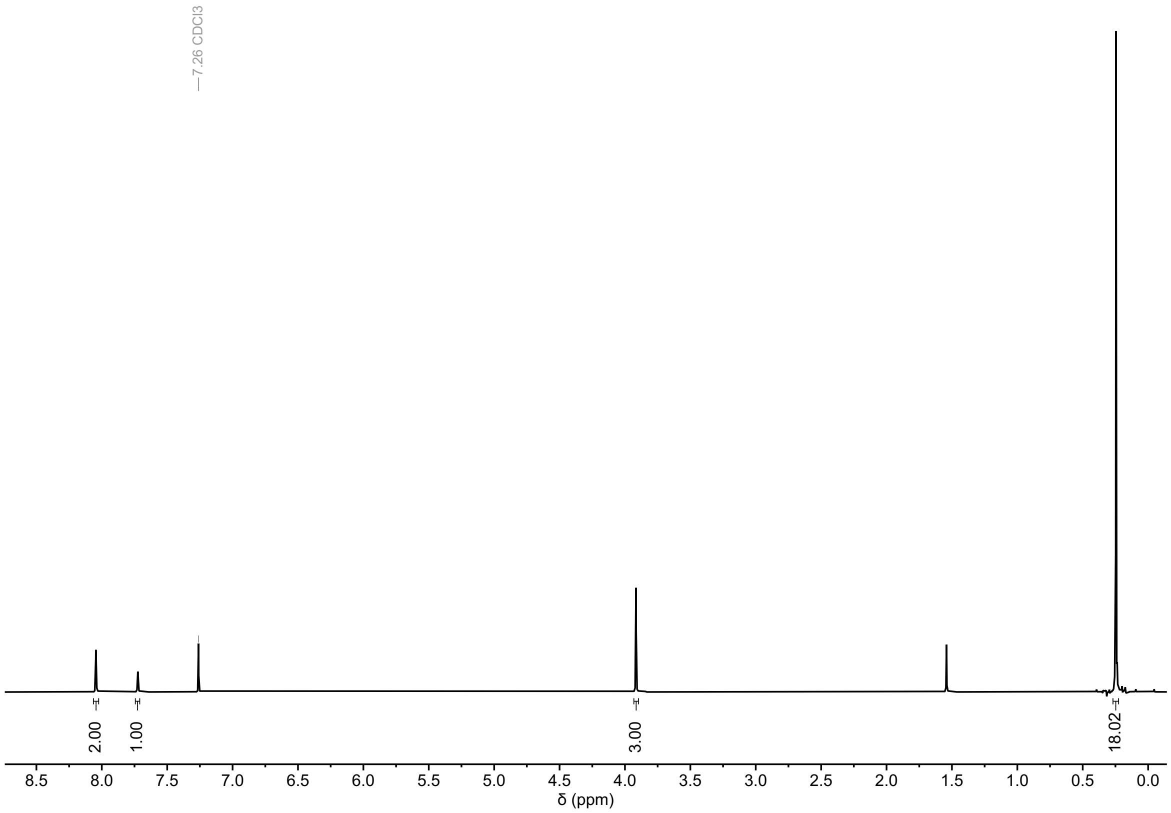

Figure S1. ^{1}H NMR (400 MHz, $CDCl_3$) of compound **2**.

Synthesis of compound 3

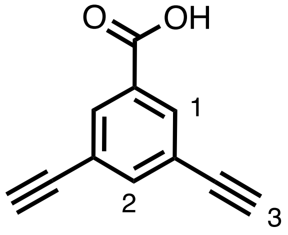

Compound **2** (250 mg, 0.76 mmol) was dissolved in a mixture of MeOH (1.5 mL) and THF (0.5 mL). A solution of KOH in H_2O (6M, 1.3 mL, 7.8 mmol) was added and the reaction mixture was stirred at room temperature for 16 hours. The solvents were removed *in vacuo* to give compound **3** as a pale yellow solid (103 mg, 80%).

^{1}H NMR (400 MHz, $(CD_3)_2SO$) δ 7.95 (d, *J* = 1.6 Hz, 2H, C1H), 7.80 (d, *J* = 1.6 Hz, 1H, C2H), 4.41 (s, 2H, C3H). *Data in agreement with literature.*[1]

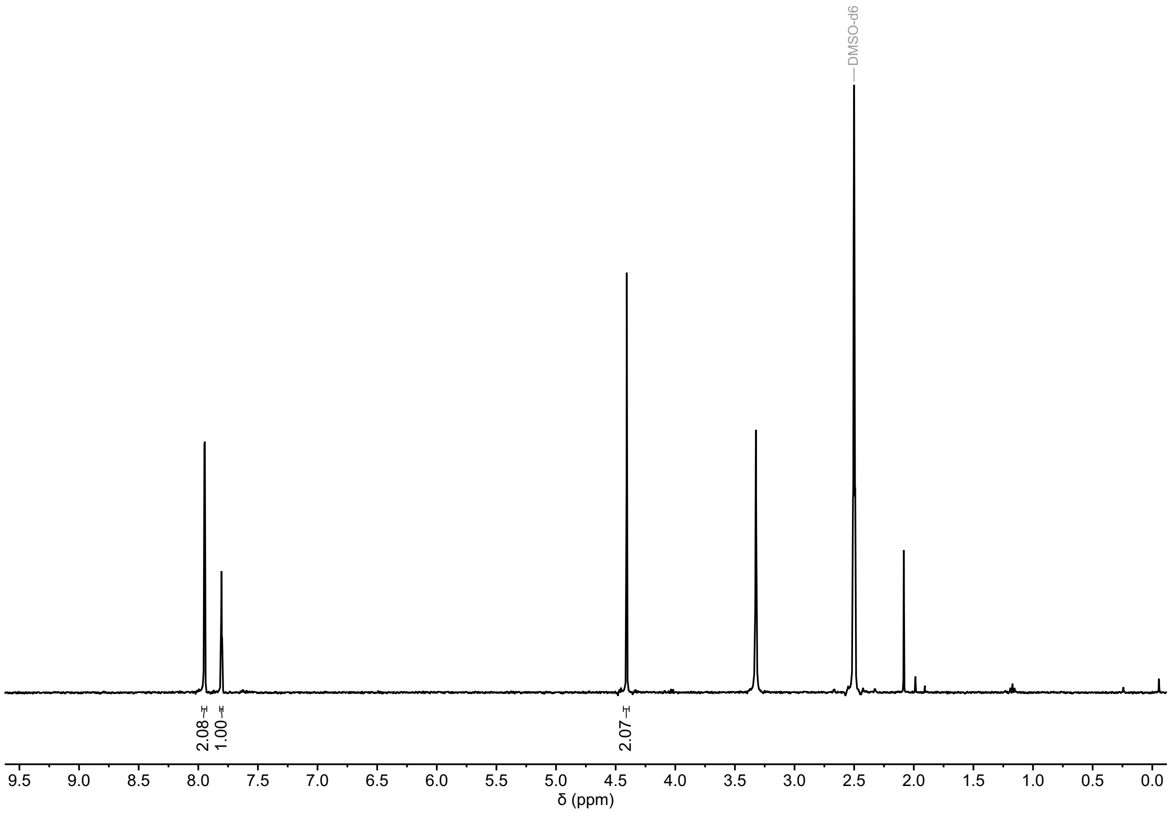

Figure S2. ^{1}H NMR (400 MHz, $(CD_3)_2SO$) of compound **3**.

Synthesis of compound 4

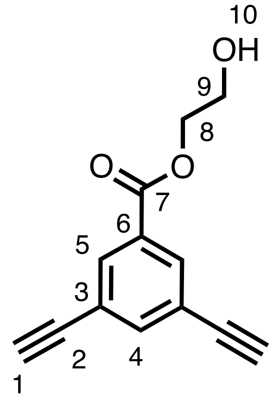

Ethylene glycol (98 μL, 0.18 mmol), EDC (67 mg, 0.352 mmol) and DMAP (43 mg, 0.352mmol) were dissolved in THF (10 mL). Compound **3** (30 mg, 0.18 mmol) was added, and the reaction mixture was stirred at room temperature for 16 hours. The solution was diluted with DCM (30 mL) and the organic phase was washed with HCl (1M, 20 mL), H_2O (20 mL), brine (20 mL) and dried with $MgSO_4$. The solvent was removed *in vacuo* to afford the product as a white solid (55 mg, 0.57 mmol, 88%).

^{1}H NMR (500 MHz, $(CD_3)_2SO$) δ 8.04 (d, *J* = 1.6 Hz, 2H, C5H), 7.83 (t, *J* = 1.6 Hz, 1H, C4H), 5.01 (t, *J* = 5.9 Hz, 1H, OH), 4.43 (s, 2H, C1H), 4.31 – 4.25 (m, 2H, C8H_2), 3.71 (q, *J* = 5.1 Hz, 2H, C9H_2).

^{13}C NMR (126 MHz, $(CD_3)_2SO$) δ 164.2 (C7), 138.5 (C4), 132.4 (C5), 131.1 (C6), 123.0 (C3), 82.9 (C1), 81.3 (C2), 67.3 (C8), 58.9 (C9).

HRMS: (ESI$^+$) calculated for $[C_{13}H_{11}O_3]^+$: 215.0703, found $[M+H]^+$: 215.0699.

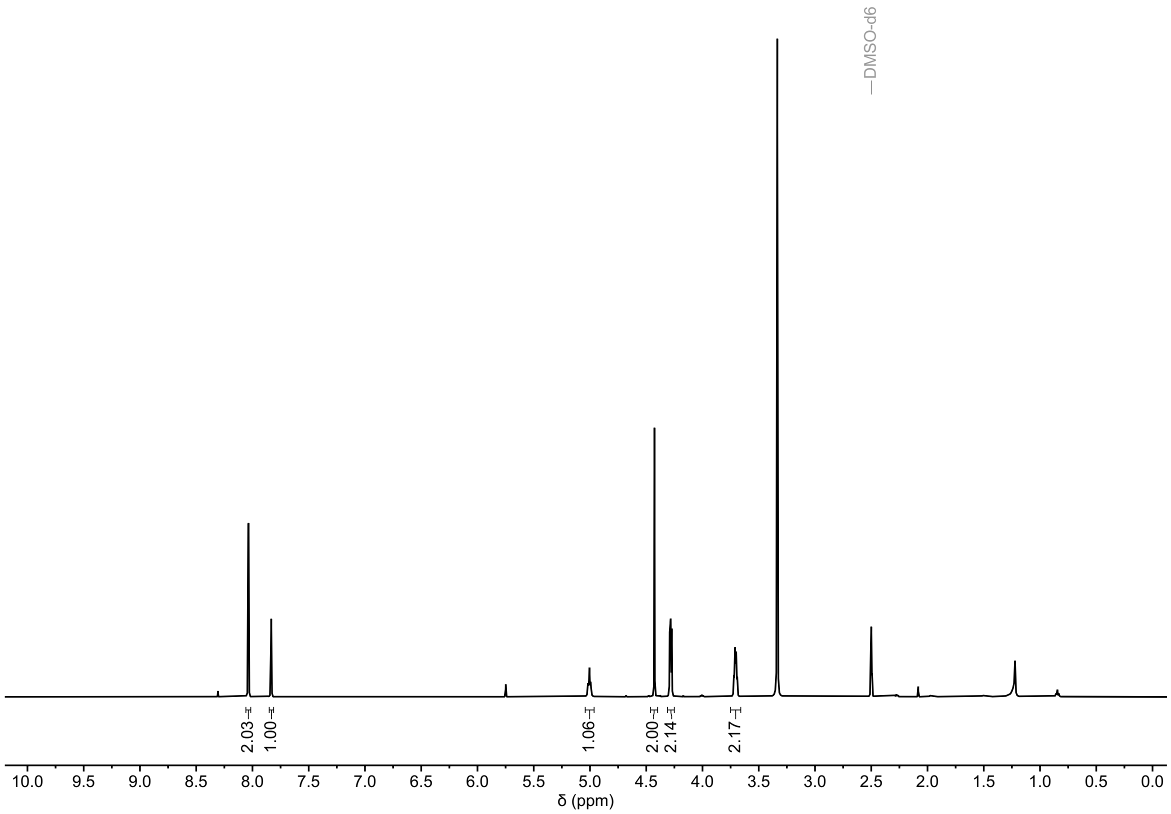

Figure S3. ^{1}H NMR (500 MHz, $(CD_3)_2SO$) of compound **4**.

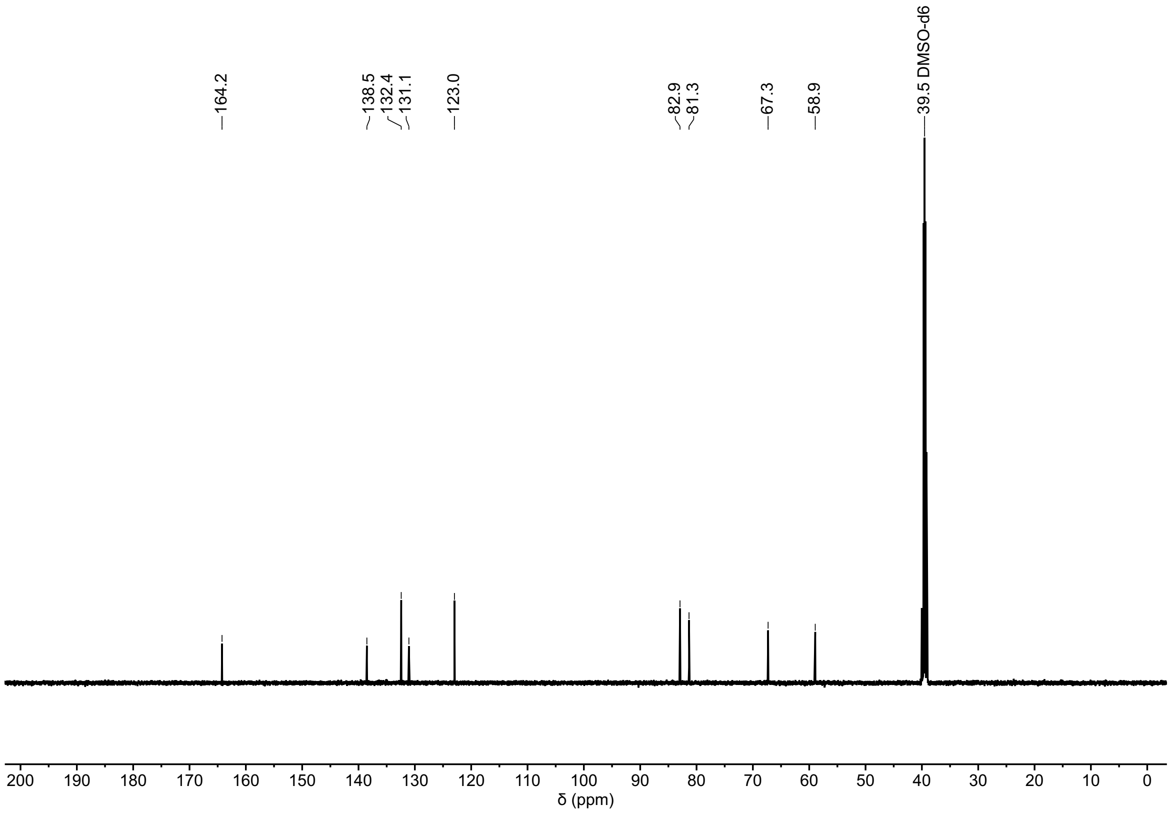

Figure S4. ^{13}C NMR (126 MHz, $(CD_3)_2SO$) of compound **4**.

2.2. Synthesis of compounds 14 and 20

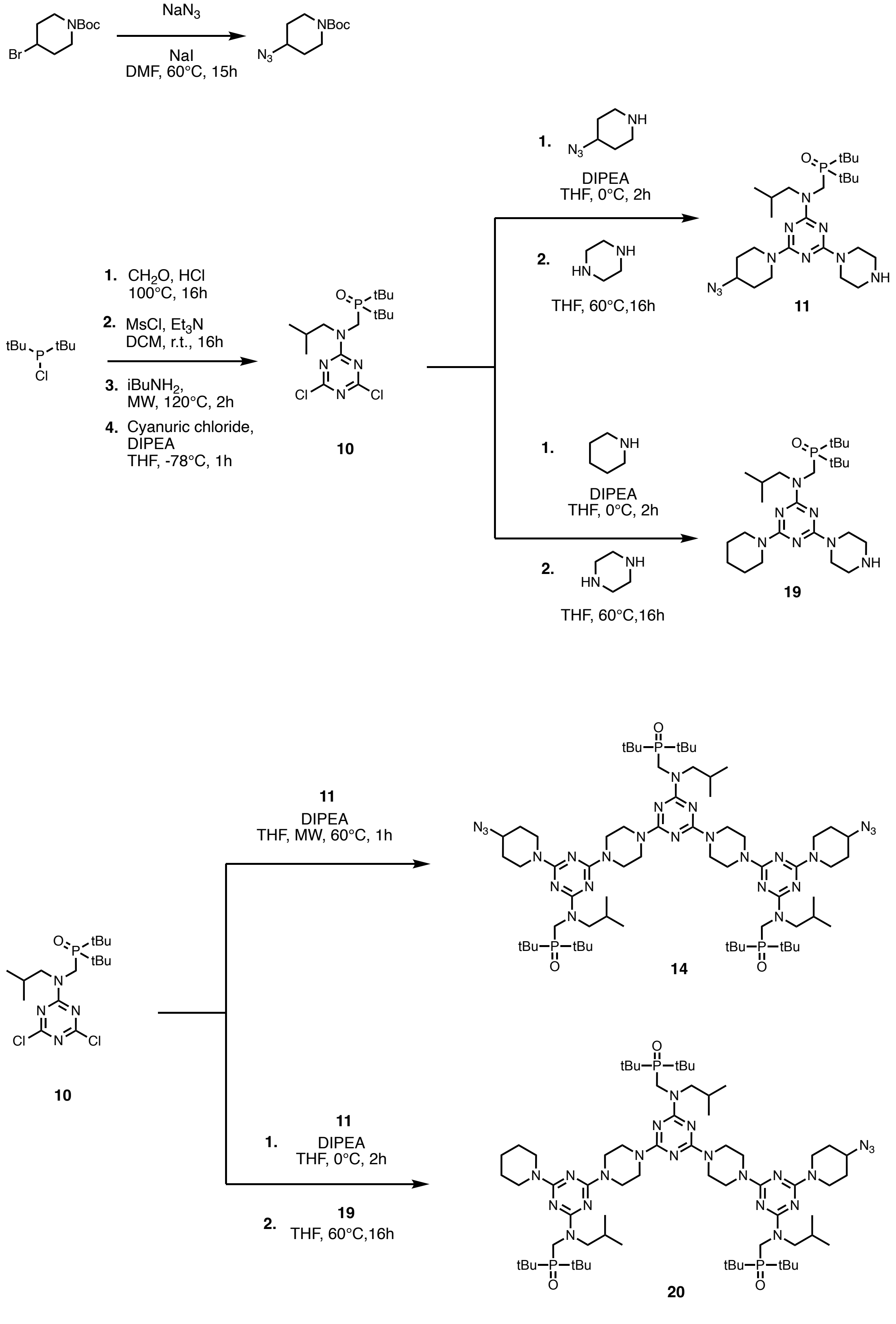

Scheme S2. Synthesis of **zAAAz 14** and **pAAAz 20**.

Synthesis of *tert*-butyl 4-azidopiperidine-1-carboxylate

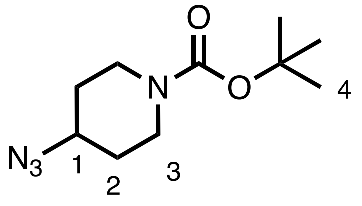

A solution of *tert*-butyl 4-bromopiperidine-1-carboxylate (500 mg, 1.893 mmol), NaN_3 (184 mg, 2.839 mmol) and NaI (28.4 mg, 0.189 mmol) in DMF (10 mL) was stirred at 60°C for 15 hours. Once cooled to room temperature, H_2O (30 mL) was added to the solution and the resulting mixture was extracted with DCM (3 x 30 mL). The combined organic layers were washed with H_2O (3 x 30 mL), LiCl solution (5% in water, 30 mL) and dried with $MgSO_4$. The solvent was removed *in vacuo* to afford the crude product. The obtained residue was purified by flash chromatography (SiO_2, 0-9% EtOAc in petroleum ether 40-60°C) to afford the title compound (412 mg, 1.82 mmol, 95%).

^{1}H NMR (400 MHz, $CDCl_3$) δ 3.82 (m, 2H, C3H), 3.56 (tt, *J* = 8.6, 3.9 Hz, 1H, C1H), 3.08 (ddd, *J* = 13.3, 9.4, 3.4 Hz, 2H, C3′H), 1.91 – 1.80 (m, 2H, C2H), 1.62 – 1.50 (m, 2H, C2′H), 1.45 (s, 9H, C4H_3). *Data in agreement with literature.*[2]

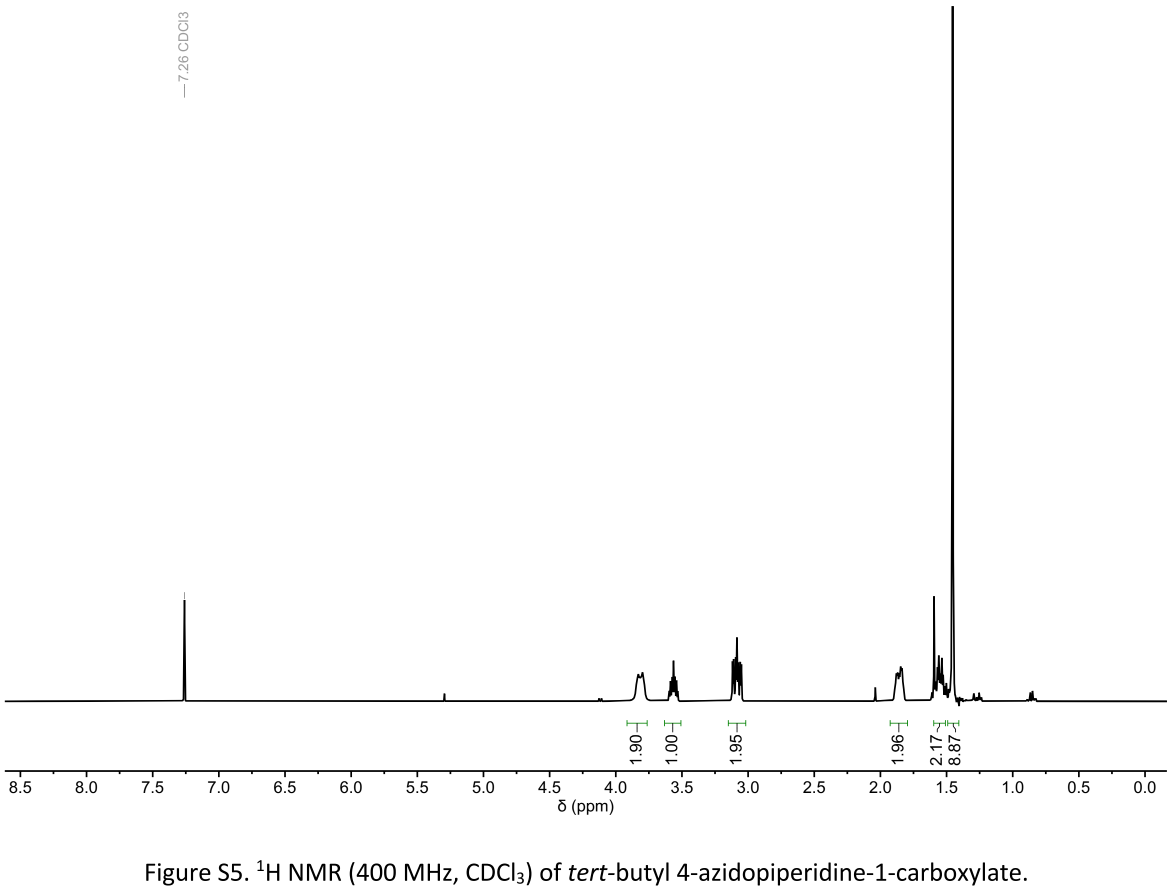

Figure S5. ^{1}H NMR (400 MHz, $CDCl_3$) of *tert*-butyl 4-azidopiperidine-1-carboxylate.

Synthesis of 4-azidopiperidine (TFA salt)

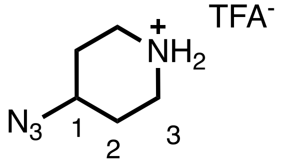

tert-Butyl 4-azidopiperidine-1-carboxylate (600 mg, 2.65 mmol) was dissolved in DCM (15 mL) and cooled to 0°C. TFA (5 mL) was added dropwise, and the solution was stirred at room temperature for 2 hours. The volatiles were removed under a flow of N_2 to get the title compound as an oil (600 mg, quant.).

^{1}H NMR (500 MHz, $CDCl_3$) δ 8.89 – 8.84 (m, 2H, N<u>H</u>$_2$), 3.88 (tt, *J* = 6.4, 3.2 Hz, 1H, C1<u>H</u>), 3.33 (m, 2H, C3<u>H</u>), 3.18 (m, 2H, C3'<u>H</u>), 2.15 (ddd, *J* = 13.8, 9.3, 4.1 Hz, 2H, C2<u>H</u>), 1.94 (m, 2H, C2'<u>H</u>).

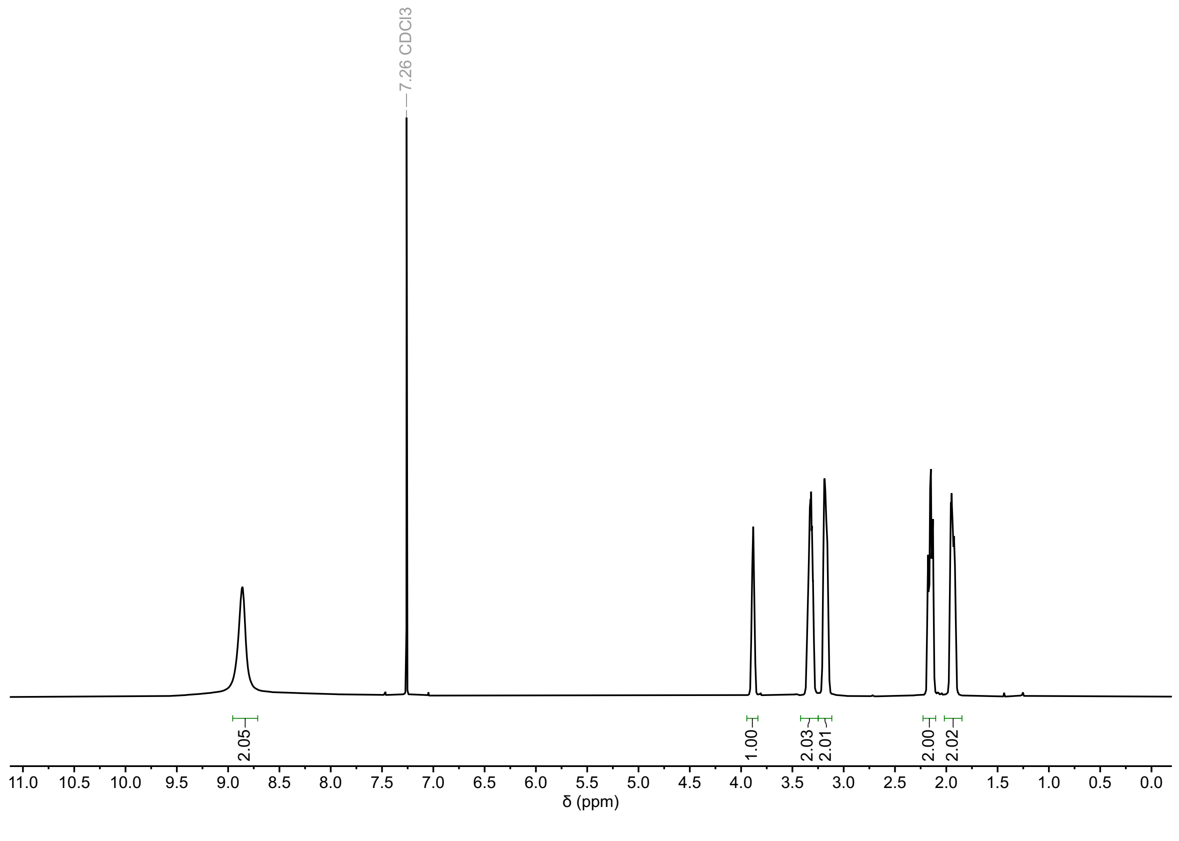

Figure S6. ^{1}H NMR (500 MHz, $CDCl_3$) of compound 4-azidopiperidine (TFA salt).

Synthesis of compound 10

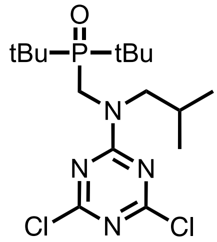

10 was made according to literature procedure.[3]

Synthesis of compound 11

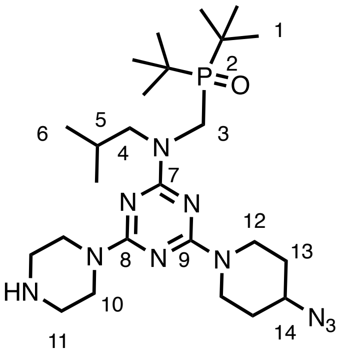

A solution of 4-azidopiperidine (TFA salt) (355 mg, 1.47 mmol) and DIPEA (770 μL, 4.42 mmol) in THF (5 mL) was added dropwise to a solution of **10** in THF (10 mL) at 0°C. The reaction mixture was stirred at 0°C for 2h. A suspension of piperazine (1.3 g, 14.7 mmol) in THF (20 mL) was then added at 0°C. The resulting suspension was warmed to room temperature and then stirred at 60°C for 16 hours. EtOAc (100 mL) was added, and the organic layer was washed with H_2O (3 x 50 mL) and dried with $MgSO_4$. The organic layer was concentrated under reduced pressure to give compound **11** as a white solid (653 mg, 83%).

^{1}H NMR (700 MHz, $CDCl_3$) δ 4.37 (s, 2H, C3$\underline{H}_2$), 4.36-4.23 (m, 2H, C12$\underline{H}$), 3.82 (d, *J* = 7.4 Hz, 2H, C4$\underline{H}_2$), 3.72 (t, *J* = 5.0 Hz, 4H, C10$\underline{H}_2$), 3.66-3.58 (m, 1H, C14$\underline{H}$), 3.31-3.18 (m, 2H, C12′$\underline{H}$), 2.86 (t, *J* = 5.0 Hz, 4H, C11$\underline{H}_2$), 2.21 – 2.15 (m, 1H, C5$\underline{H}$), 1.93-1.87 (m, 2H, C13$\underline{H}$), 1.83 (s, br, 1H, N$\underline{H}$), 1.62-1.50 (m, 2H, C13′$\underline{H}$), 1.26 (d, *J* = 12.8 Hz, 18H, C1$\underline{H}_3$), 0.87 (d, *J* = 6.7 Hz, 6H, C6$\underline{H}_3$).
^{13}C NMR (176 MHz, $CDCl_3$) δ 165.6-165.0, 58.3-58.4 (C14), 53.1 (C4), 46.2 (C11), 44.5 (C10), 41.0 (C12), 38.1 (d, *J* = 59.1 Hz, C3), 35.9 (d, *J* = 55.5 Hz, C2), 30.7 (C13), 26.9 (C1), 26.6 (C5), 20.7 (C6).
^{31}P NMR (203 MHz, $CDCl_3$) δ 58.57.
HRMS: (ESI$^+$) calculated for $[C_{27}H_{47}N_{10}OPNa]^+$: 557.3564, found $[M+Na]^+$: 557.3575.

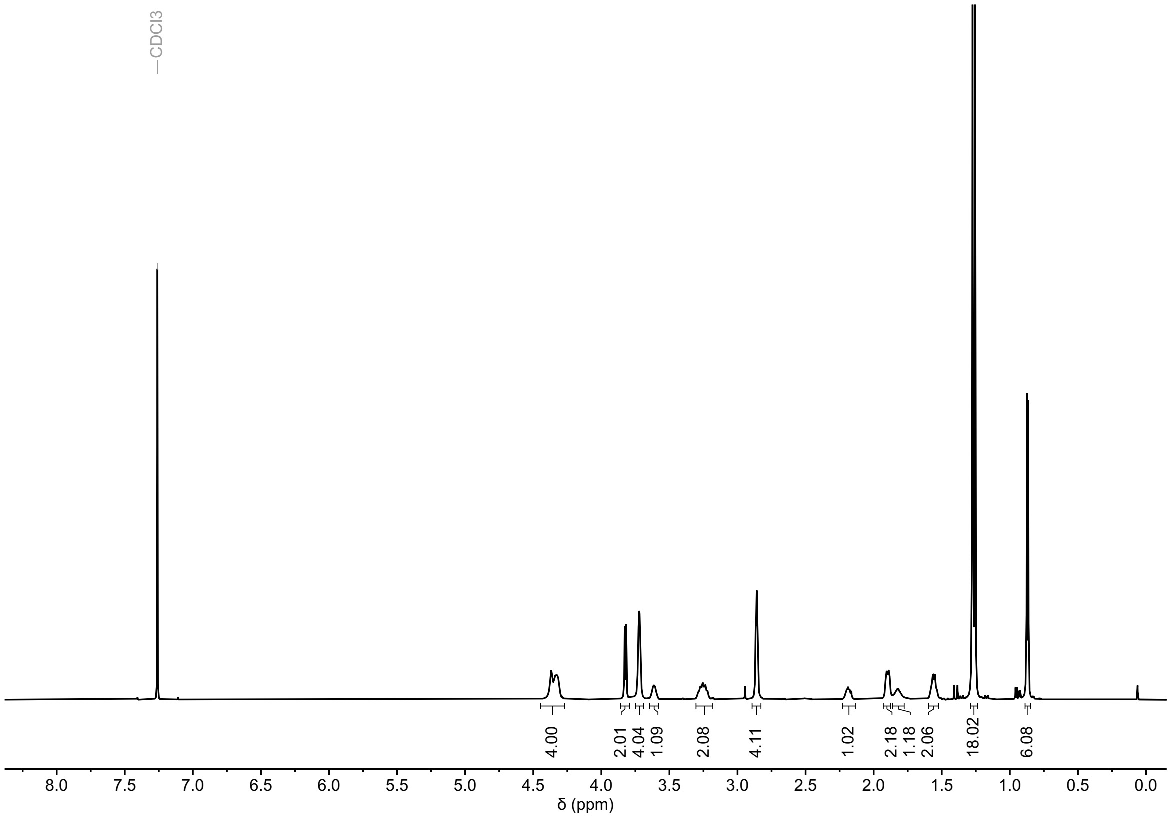

Figure S7. ^{1}H NMR (700 MHz, $CDCl_3$) of compound **11**.

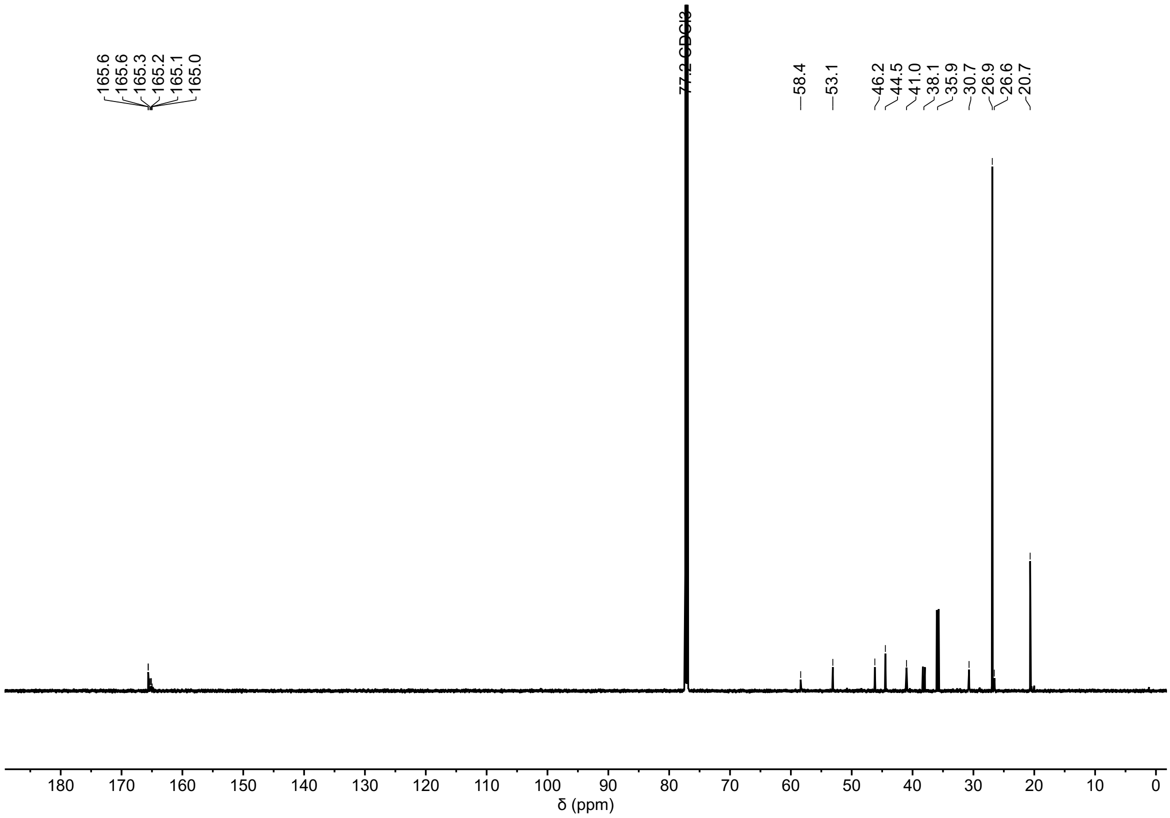

Figure S8. ^{13}C NMR (176 MHz, $CDCl_3$) of compound **11**.

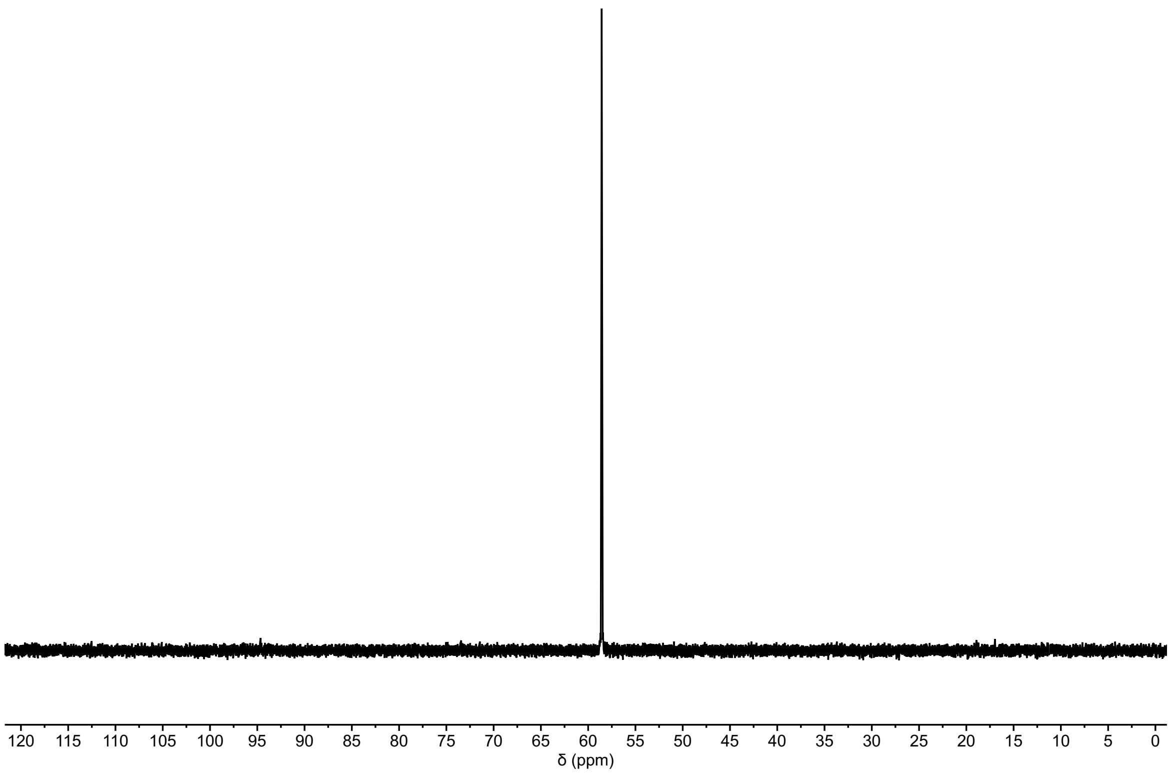

Figure S9. ^{31}P NMR (203 MHz, $CDCl_3$) of compound **11**.

Synthesis of compound 19

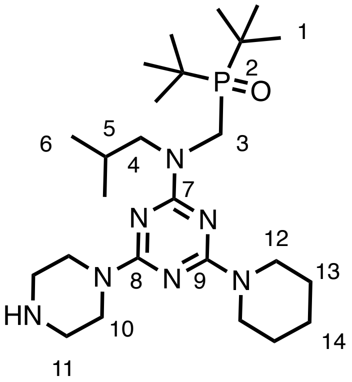

A solution of piperidine (0.172 mL, 1.74 mmol) and DIPEA (0.610 mL, 3.5 mmol) in THF (5 mL) was added dropwise to a solution of **10** (690 mg, 1.74 mmol) in THF (5 mL) at 0°C. The resulting mixture was stirred at 0°C for 2 hours and the formation of the disubstituted compound was monitored by HPLC. A suspension of piperazine (1.6 g, 18.6 mmol) in THF (15 mL) was then added at 0°C. The resulting suspension was warmed to room temperature and then stirred at 60°C for 16 hours. EtOAc (50 mL) was added to the solution and the organic layer was washed with H_2O (3 x 30 mL) and dried with $MgSO_4$. The solvent was removed *in vacuo* to afford the product as a white solid (820 mg, 95%).

^{1}H NMR (500 MHz, $CDCl_3$) δ 4.36 (d, *J* = 2.8 Hz, 2H, C3$\underline{H}_2$), 3.80 (d, *J* = 7.4 Hz, 2H, C4$\underline{H}_2$), 3.73-3.64 (m, 8H, C10$\underline{H}_2$-C12$\underline{H}_2$), 2.84 (t, *J* = 5.1 Hz, 4H, C11$\underline{H}_2$), 2.17 (non, *J* = 6.9 Hz, 1H, C5$\underline{H}$), 2.06 (br, 1H, N$\underline{H}$), 1.65- 1.57 (m, 2H, C14$\underline{H}_2$), 1.55 – 1.47 (m, 4H, C13$\underline{H}_2$), 1.24 (d, *J* = 12.9 Hz, 18H, C1$\underline{H}_3$), 0.85 (d, *J* = 6.7 Hz, 6H, C6$\underline{H}_3$). *Data in agreement with literature.*[3]

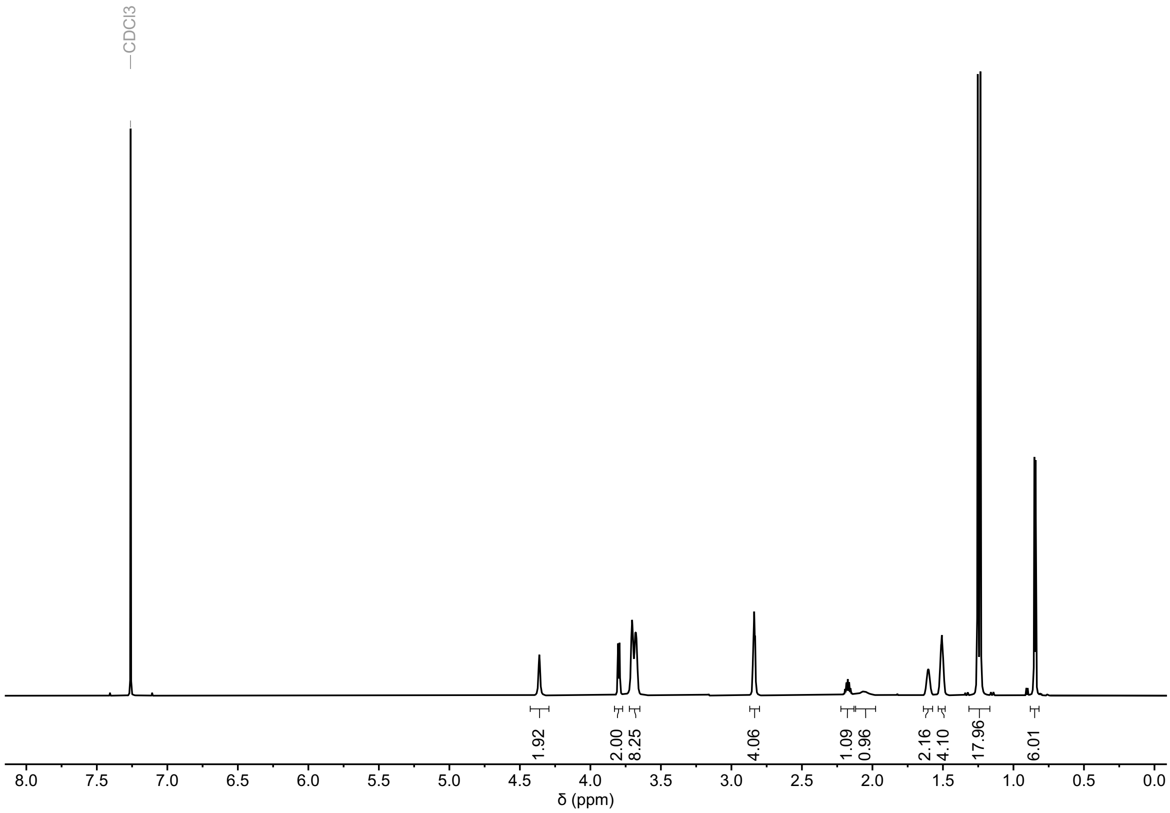

Figure S10. ^{1}H NMR (500 MHz, $CDCl_3$) of compound **19**.

Synthesis of compound 14

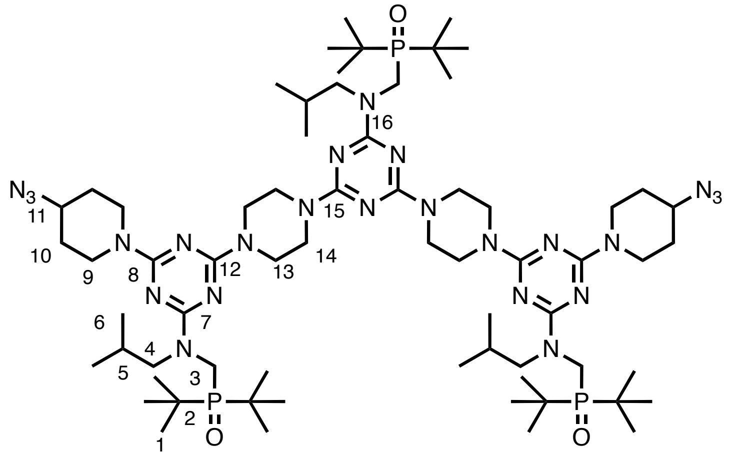

A solution of **10** (0.150 g, 0.37 mmol), **11** (1 g, 1.87 mmol) and DIPEA (110 µL, 0.63 mmol) in THF (3.7 mL) was microwaved at 60°C for 1h. EtOAc (30 mL) was added to the solution and the organic layer was washed with H_2O (3 x 30 mL) and dried with $MgSO_4$. The solvent was removed *in vacuo* to afford the crude product. The obtained residue was purified by flash chromatography (SiO_2, 0-15% MeOH in DCM) to afford the title compound as a white solid (420 mg, 82%).

^{1}H NMR (700 MHz, $CDCl_3$) δ 4.45-4.35 (m, 6H, C3$\underline{H}_2$), 4.35-4.27 (m, 4H, C9$\underline{H}$), 3.90-3.81 (m, 6H, C4$\underline{H}_2$), 3.81-3.71 (m, 16H, C13$\underline{H}_2$-C14$\underline{H}_2$), 3.66-3.57 (m, 2H, C11$\underline{H}$), 3.36-3.19 (m, 4H, C9′$\underline{H}$), 2.23-2.16 (m, 3H, C5$\underline{H}$), 1.96-1.89 (m, 4H, C10$\underline{H}$), 1.62-1.55 (m, 4H, C10′$\underline{H}$), 1.30 (m, 54H, C1$\underline{H}_3$), 0.92-0.87 (m, 18H, C6$\underline{H}_3$).

^{13}C NMR (176 MHz, $CDCl_3$) δ 165.6-164.8 (C=X), 58.2 (C11), 53.2 (C4), 43.2 (C13-C14), 41.0 (C9), 38.1 (d, J = 58.9 Hz, C3), 36.0 (d, J = 55.5 Hz, C2), 30.7 (C10), 26.9 (C1), 26.6 (C5), 20.7 (C6).

^{31}P NMR (203 MHz, $CDCl_3$) δ 58.99, 58.90.

HRMS: (ESI^+) calculated for $[C_{66}H_{122}N_{24}O_3P_3]^+$: 1391.9345, found $[M+H]^+$: 1391.9320.

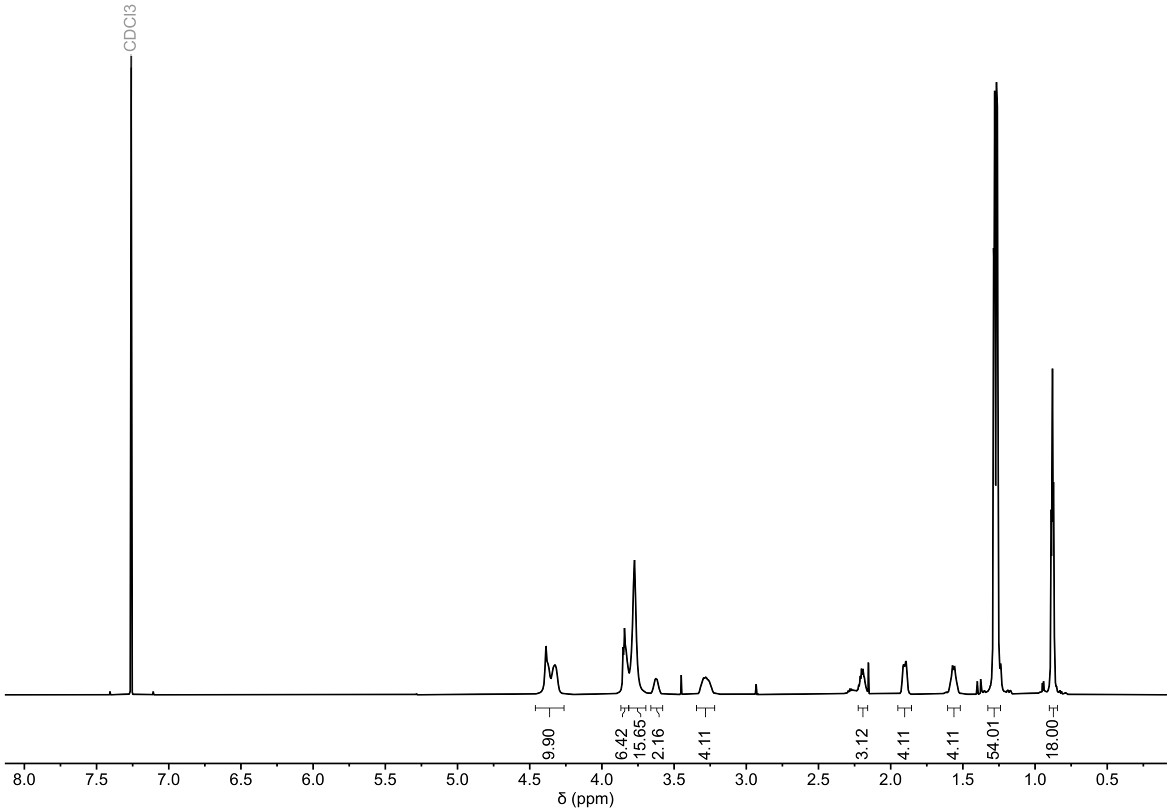

Figure S11. ^{1}H NMR (700 MHz, $CDCl_3$) of compound **14**.

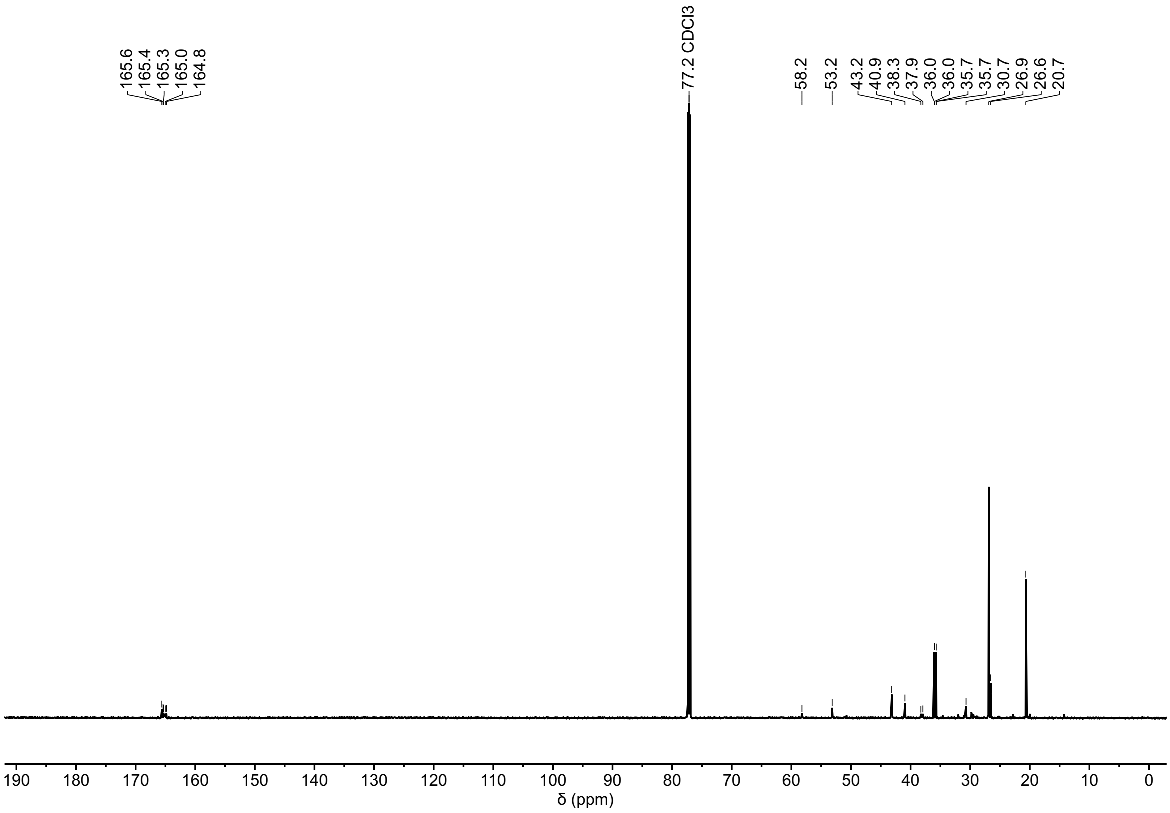

Figure S12. ^{13}C NMR (176 MHz, $CDCl_3$) of compound **14**.

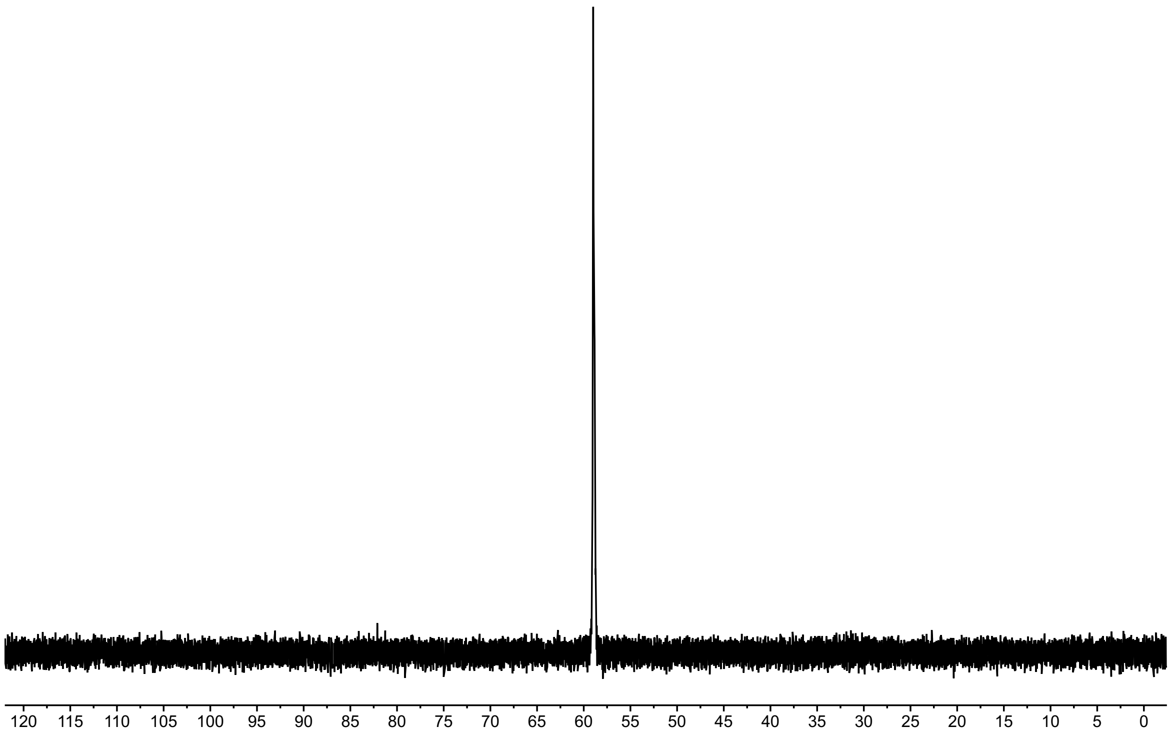

Figure S13. ^{31}P NMR (203 MHz, $CDCl_3$) of compound **14**.

Synthesis of compound 20

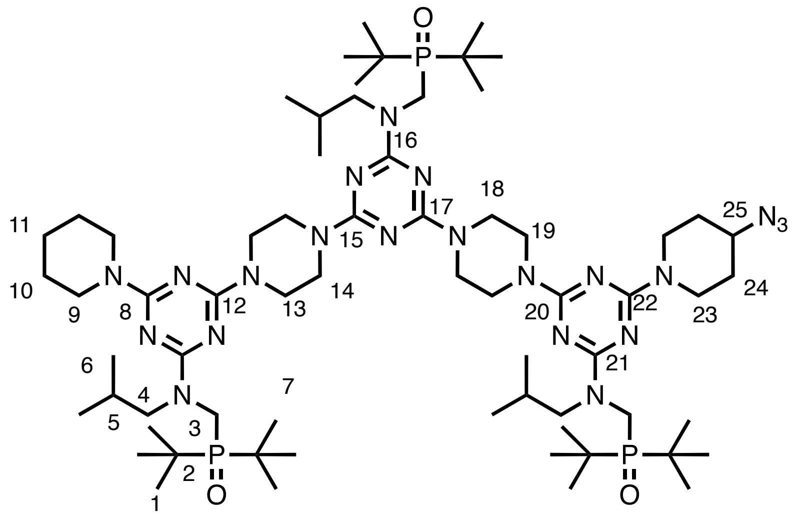

A solution of **11** (40.0 mg, 74.8 μmol) and DIPEA (0.039 mL, 0.224 mmol) in THF (0.25 mL) was added dropwise to a solution of **10** (32.4 mg, 82.2 μmol) in THF (0.25 mL) at 0°C. The resulting solution was stirred at 0°C for 2 hours and monitored by UPLC. A solution of **19** (73.8 mg, 0.1496 mmol) in THF (0.25 mL) was added to the reaction mixture and the resulting suspension was stirred at 60°C for 16h. EtOAc (30 mL) was added to the solution and the organic layer was washed with H_2O (3 x 30 mL) and dried with $MgSO_4$. The solvent was removed *in vacuo* to afford the crude product. The obtained residue was purified by flash chromatography (SiO_2, 0-7 % MeOH in DCM) to afford compound **20** (91.86 mg, 0.068 mmol, 91%).

^{1}H NMR (700 MHz, $CDCl_3$) δ 4.39 (s, 6H, C3$\underline{H}_2$), 4.36-4.28 (m, 2H, C23$\underline{H}$), 3.88-3.82 (m, 6H, C4$\underline{H}_2$), 3.82-3.75 (m, 16H, C13$\underline{H}_2$- C14$\underline{H}_2$- C18$\underline{H}_2$-C19$\underline{H}_2$), 3.75-3.70 (m, 4H, C9$\underline{H}_2$),3.68-3.61 (m, 1H, C25$\underline{H}$), 3.37-3.22 (m, 2H, C23'$\underline{H}$),2.24 – 2.17 (m, 3H, C5$\underline{H}$), 1.94- 1.84 (m, 2H, C24$\underline{H}$), 1.68-1.62 (m, 2H, C11$\underline{H}_2$), 1.59-1.50 (m, 6H, C10$\underline{H}_2$-C24'$\underline{H}$), 1.28 (m, 56H, C1$\underline{H}_3$), 0.89 (m, 18H, C6$\underline{H}_3$).

^{13}C NMR (176 MHz, $CDCl_3$) δ 165.9-164.4 (C=N), 58.3 (C25), 53.2 (C4), 44.3 (C9), 43.2 (C13-C14-C18-C19), 41.0 (C23), 38.1 (d, *J* = 56.7 Hz, C3), 35.9 (d, *J* = 55.5 Hz, C2), 30.7 (C24), 26.9 (C1), 26.6 (C5), 26.0 (C10), 25.1 (C11), 20.7 (C6).

^{31}P NMR (203 MHz, $CDCl_3$) δ 58.98, 58.81.

HRMS: (ESI^+) calculated for $[C_{66}H_{122}N_{21}O_3P_3]^+$: 1350.9331, found $[M+H]^+$: 1350.9373.

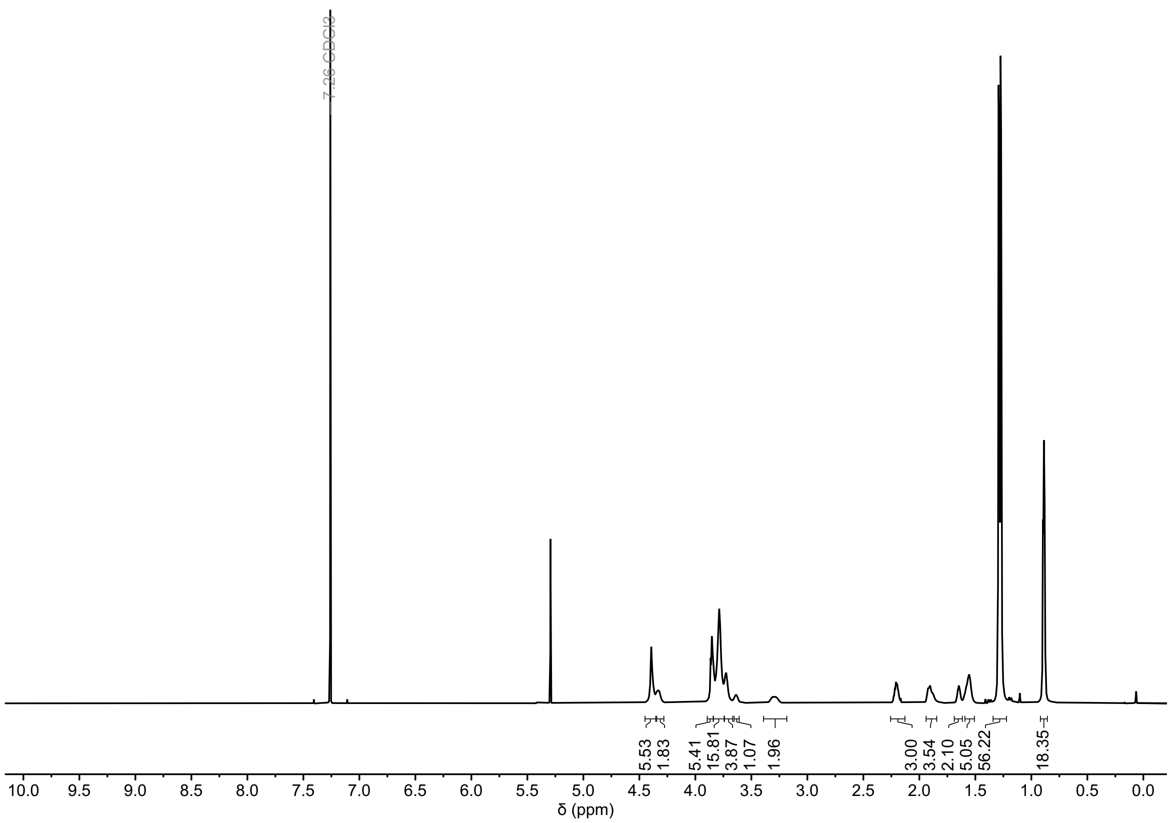

Figure S14. ^{1}H NMR (700 MHz, $CDCl_3$) of compound **20**.

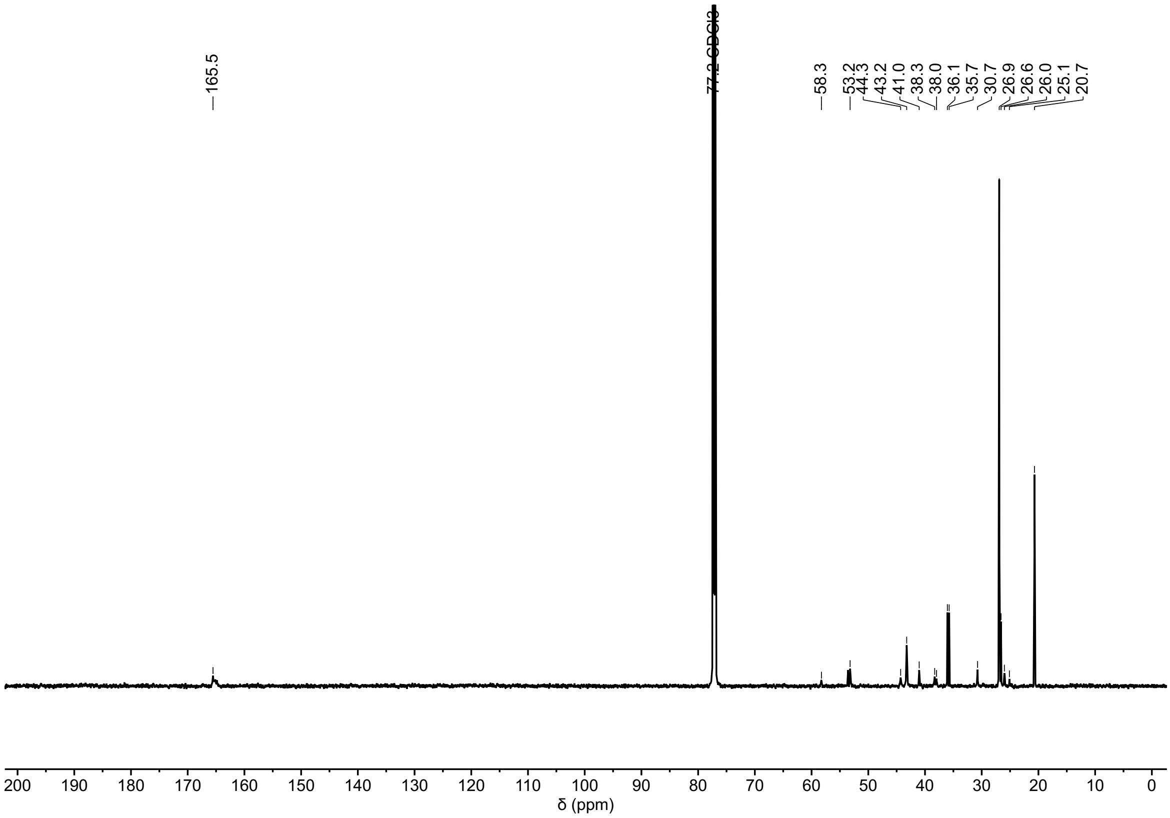

Figure S15. ^{13}C NMR (176 MHz, $CDCl_3$) of compound **20**.

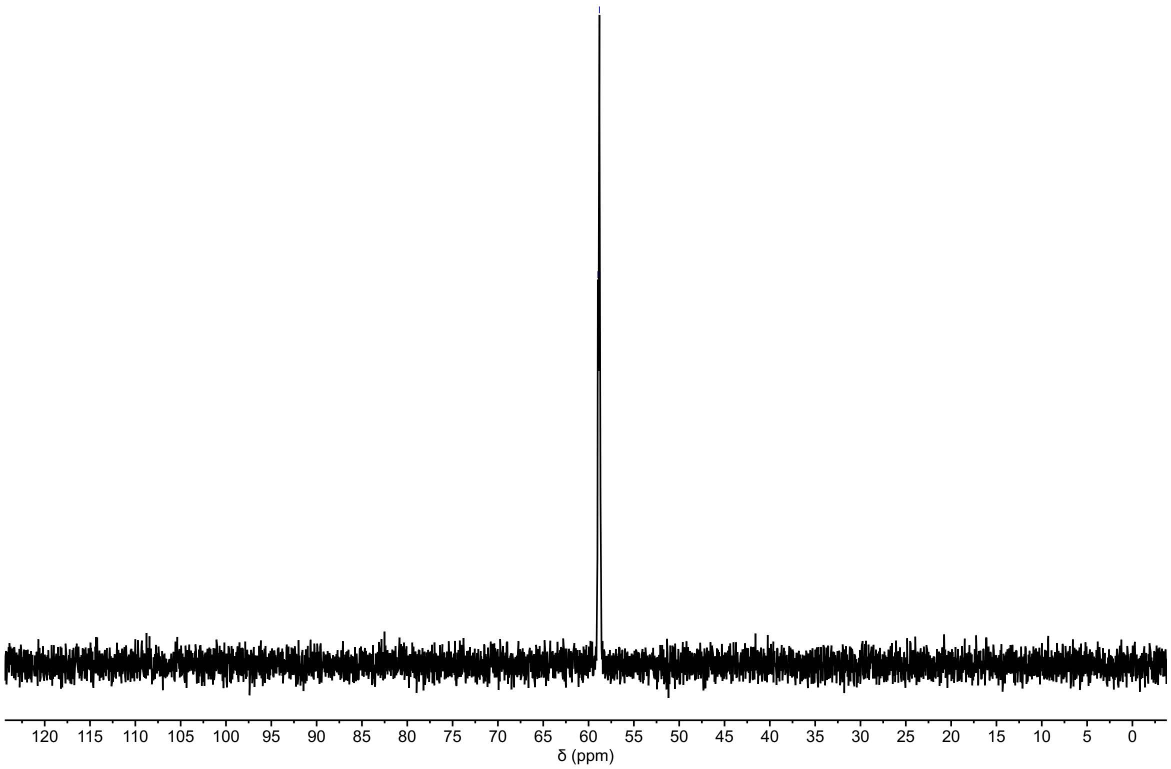

Figure S16. ^{31}P NMR (203 MHz, $CDCl_3$) of compound **20**.

2.3 Synthesis and characterization of the 7-mer template

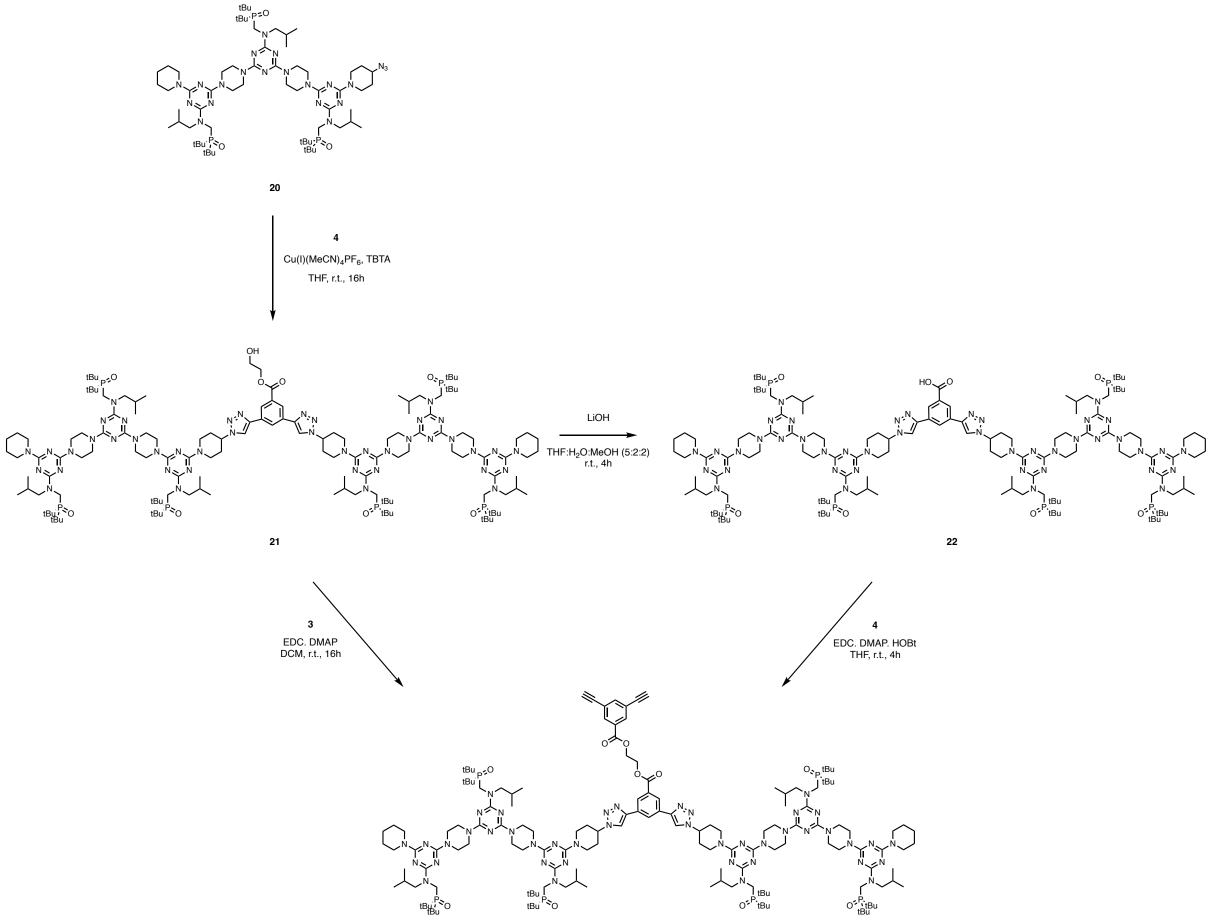

Scheme S3. Synthesis of **pAAA-C-AAAp** template.

Synthesis of compound 21

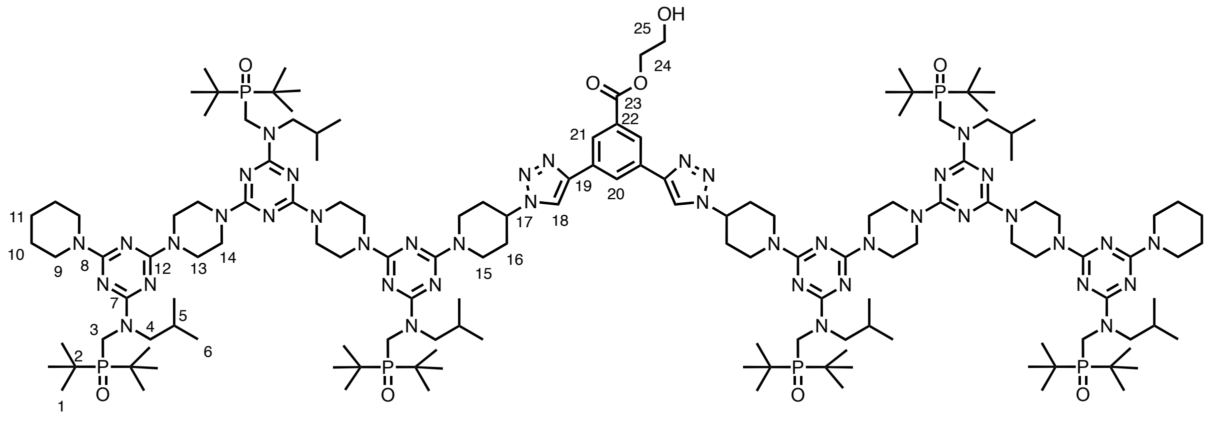

A solution of **4** (3.60 mg, 0.017mmol), **20** (50.00 mg, 0.037 mmol), $[Cu(MeCN)_4]PF_6$ (12.52 mg, 0.034 mmol) and TBTA (17.82 mg, 0.034 mmol) in THF (7 mL) was stirred at room temperature for 16 hours and monitored by HPLC. The reaction mixture was diluted with EtOAc and the organic phase was washed with basic EDTA/NH_4OH(aq) (2 x 10 mL), H_2O (2 x 20 mL), brine (20 mL) and dried with $MgSO_4$. The solvent was removed *in vacuo* and the crude compound was purified by column chromatography (SiO_2, 0-10 % MeOH in DCM) to afford the product as a white solid (24 mg, 48%).

^{1}H NMR (500 MHz, $CDCl_3$) δ 8.56 (s, 1H, C20H), 8.41 (s, 2H, C21H), 7.95 (s, 2H, C18H), 5.02- 4.84 (m, 4H, C15H), 4.81-4.70 (m, 2H, C17H), 4.48 (t, *J* = 4.8 Hz, 2H, C24H_2), 4.38 (s, 12H, C3H_2), 3.96 (t, *J* = 4.9 Hz, 2H, C25H_2), 3.90 – 3.75 (m, 44H, C4H_2-C13H_2-C14H_2), 3.73-3.66 (m, 8H, C9H_2), 3.04 (m, 4H, C15′H), 2.34-2.25 (m, 4H, C16H), 2.25-2.12 (m, 6H, C5H), 2.09-1.94 (m, 4H, C16′H), 1.66-1.57 (m, 4H, C11H_2) 1.57-1.47 (m, 8H, C10H_2), 1.31-1.21 (m, 108H, C1H_3), 0.92-0.83 (m, 36H, C6H_3).
^{13}C NMR (126 MHz, $CDCl_3$) δ 165.6 (C=X), 146.5 (C19), 131.8, 131.5, 126.9 (C20), 126.3 (C21), 118.4 (C18), 67.2 (C24), 60.9 (C25), 59.0 (C17), 53.1 (C4), 44.2 (C9), 43.2 (C13-C14), 42.1 (C15), 38.1 (d, *J* = 57.2 Hz, C3), 35.8 (d, *J* = 55.9 Hz, C2), 32.6 (C16), 26.9 (C1), 26.5 (C5), 26.0 (C10), 25.1 (C11), 20.7 (C6).
HRMS: (ESI^+) calculated for $[C_{145}H_{256}N_{42}O_9P_6]^{2+}$: 1457.9640, found $[M+2H]^{2+}$: 1457.9649.

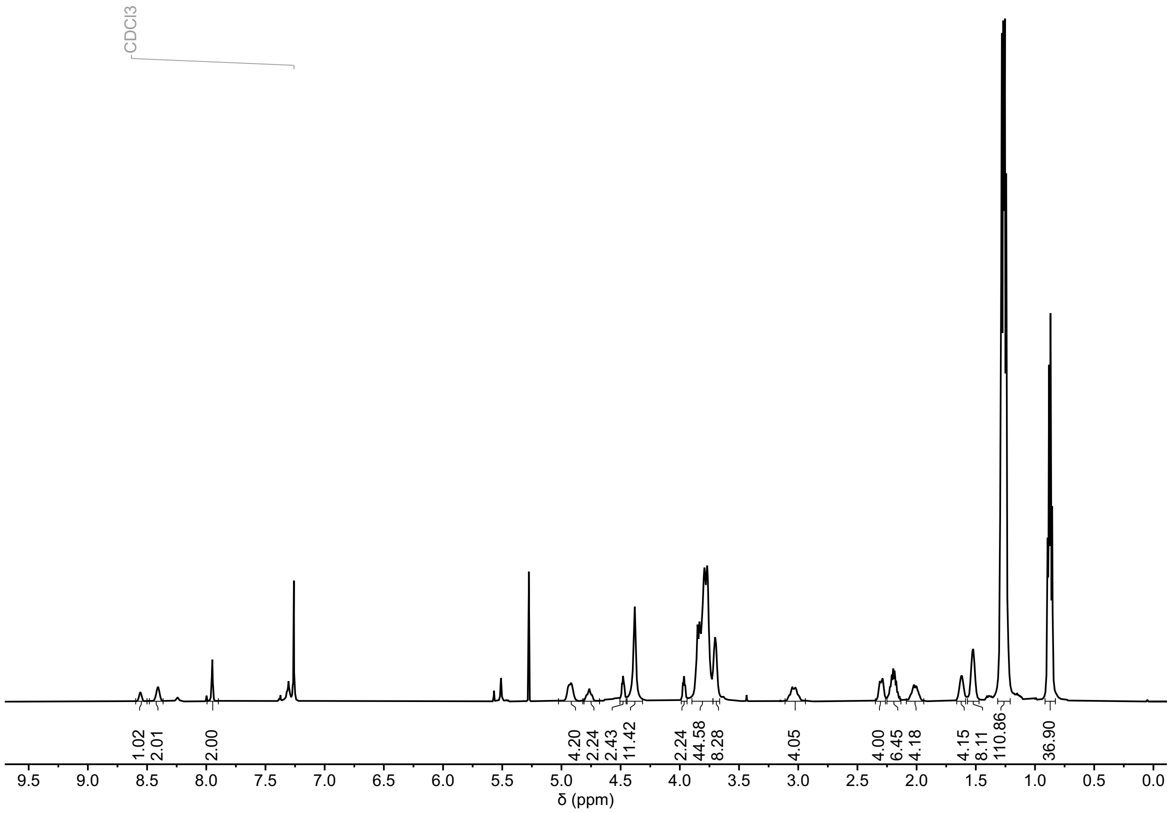

Figure S17. ^{1}H NMR (700 MHz, $CDCl_3$) of compound **21**.

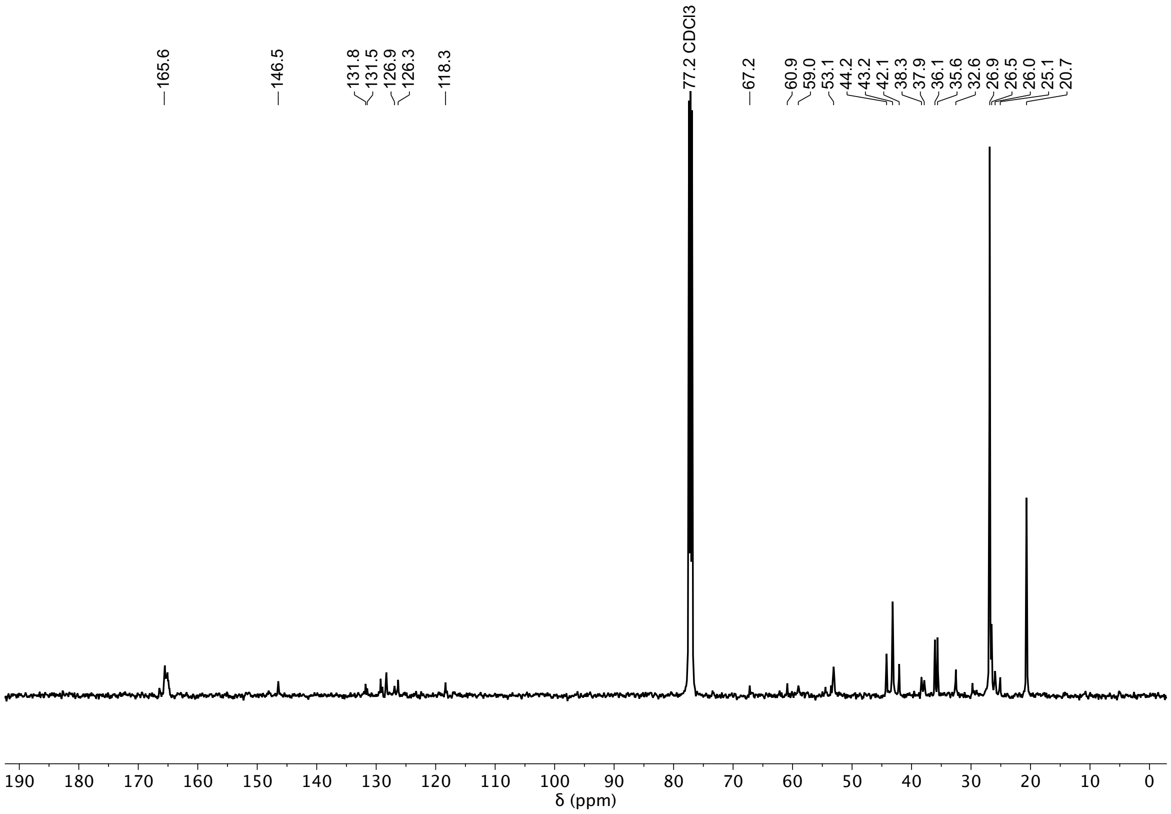

Figure S18. ^{13}C NMR (176 MHz, $CDCl_3$) of compound **21**.

Synthesis of the 7-mer template

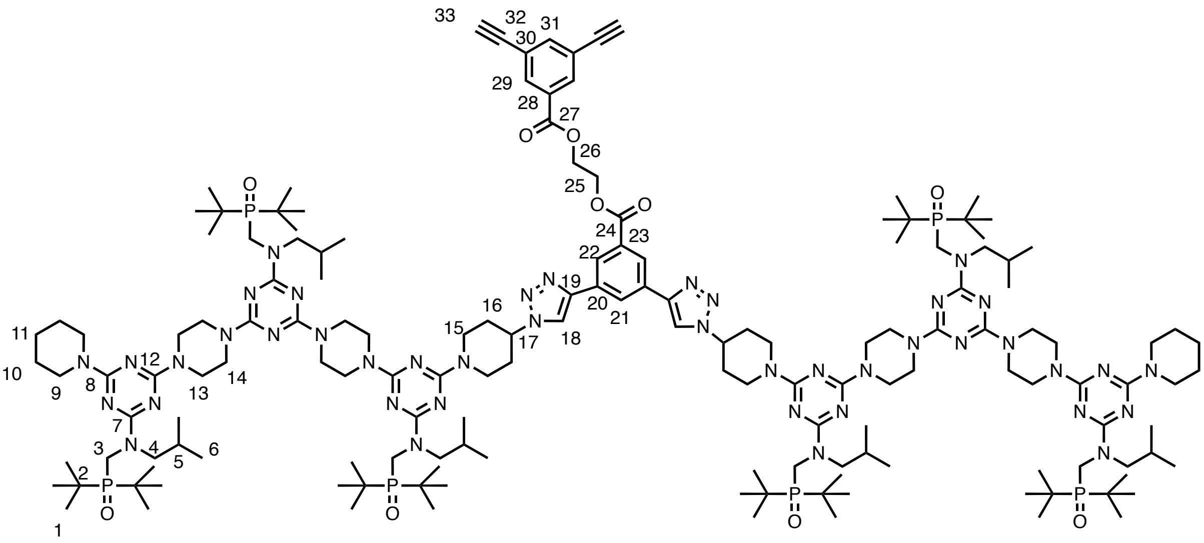

Compound **3** (2.92 mg, 0.017 mmol) was added to a solution of compound **21** (45.00 mg, 0.015 mmol), EDC (3.60 mg, 0.0188 mmol) and DMAP (3.14 mg, 0.025 mmol) in anhydrous DCM (5 mL) and the reaction mixture was stirred at room temperature for 16 hours. EtOAc was added (50 mL) and the organic phase was washed with HCl (1M, 20 mL), H_2O (20 mL), brine (20 mL) and dried with $MgSO_4$. The solvent was removed under reduced pressure to afford the crude mixture as a white solid. The crude was purified by HPLC (C8, 5% CH_3CN in H_2O: THF 45:55) to afford the 7-mer template.

^{1}H NMR (700 MHz, $CDCl_3$) δ 8.61 (s, 1H, C21H), 8.42 (s, 2H, C22H), 8.10 (d, *J* = 1.6 Hz, 2H, C29H), 7.95 (s, 2H, C18H), 7.73 (d, *J* = 1.9 Hz, 1H, C31H), 4.99-4.88 (m, 4H, C15H), 4.81-4.74 (m, 2H, C17H), 4.71 (s, 4H, C25H_2-C26H_2), 4.40 (s, 12H, C3H_2), 3.88 – 3.67 (m, 52H, C4H_2-C9H_2-C13H_2-C14H_2), 3.12 (s, 2H, C33H), 3.11-2.99 (m, 4H, C15′H), 2.33 – 2.29 (m, 4H, C16H_2), 2.25-2.14 (m, 6H, C5H), 2.07-2.00 (m, 4H, C16H_2), 1.66-1.60 (m, 4H, C11H_2), 1.57-1.52 (m, 8H, C10H_2), 0.92 – 0.85 (m, 108H, C1H_3), 0.85 – 0.79 (m, 36H, C6H_3).

^{13}C NMR (176 MHz, $CDCl_3$) δ 166.2-164.6 (C=N), 139.6 (C31), 133.5 (C29), 130.66, 127.2 (C21), 126.4 (C22), 123.3, 118.4 (C18), 108.0, 81.6, 79.3 (C33), 63.3/63.1 (C25-C26), 59.0 (C17), 53.2 (C4), 44.2 (C9), 43.2 (C13-C14), 42.1 (C15), 38.2 (d, *J* = 58.4 Hz, C3), 36.0 (dm, *J* = 55.4 Hz, C2), 35.7, 32.6 (C16), 31.4, 29.3, 26.9 (C1), 26.6 (C5), 26.0 (C10), 25.1 (C11), 24.0, 20.7 (C6).

^{31}P NMR (203 MHz, $CDCl_3$) δ 59.49, 59.24.

HRMS: (ESI$^+$) calculated for $[C_{156}H_{260}N_{42}O_{10}P_6]^{2+}$: 1533.9771, found $[M+2H]^{2+}$: 1533.9834.

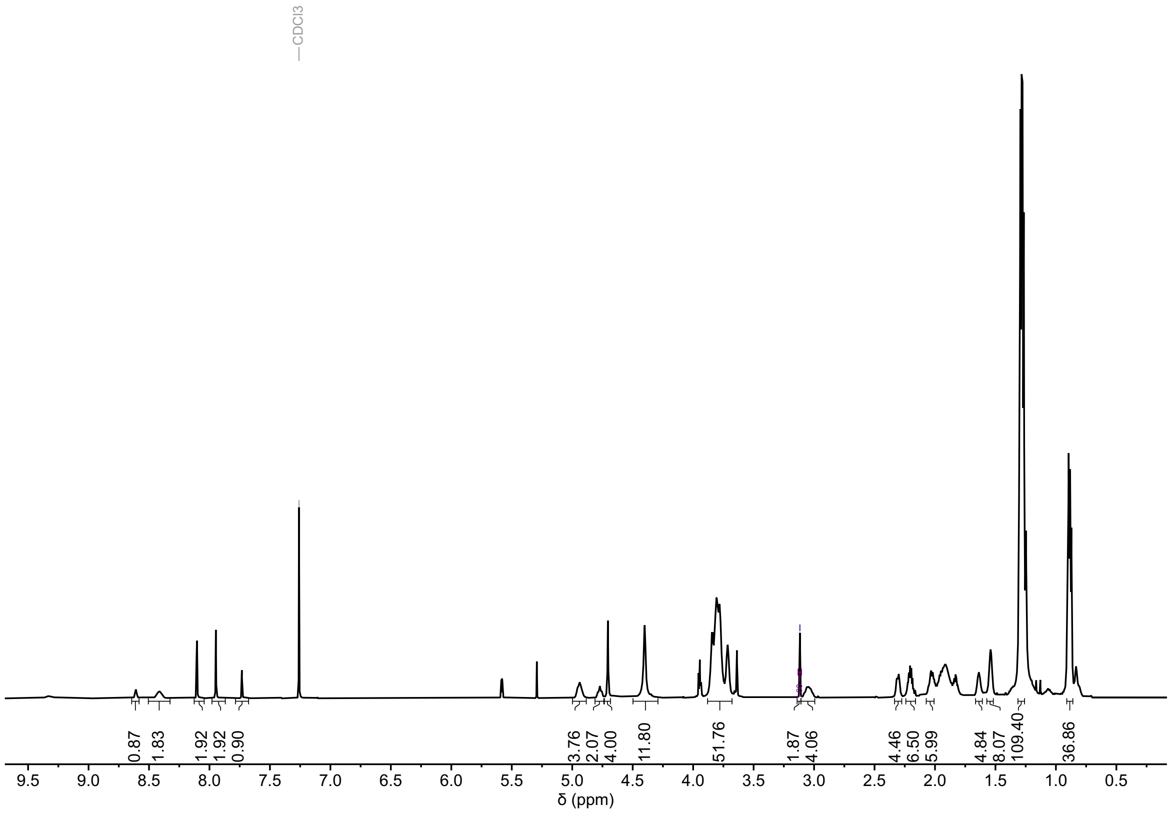

Figure S19. ^1H NMR (700 MHz, $CDCl_3$) of the 7-mer template.

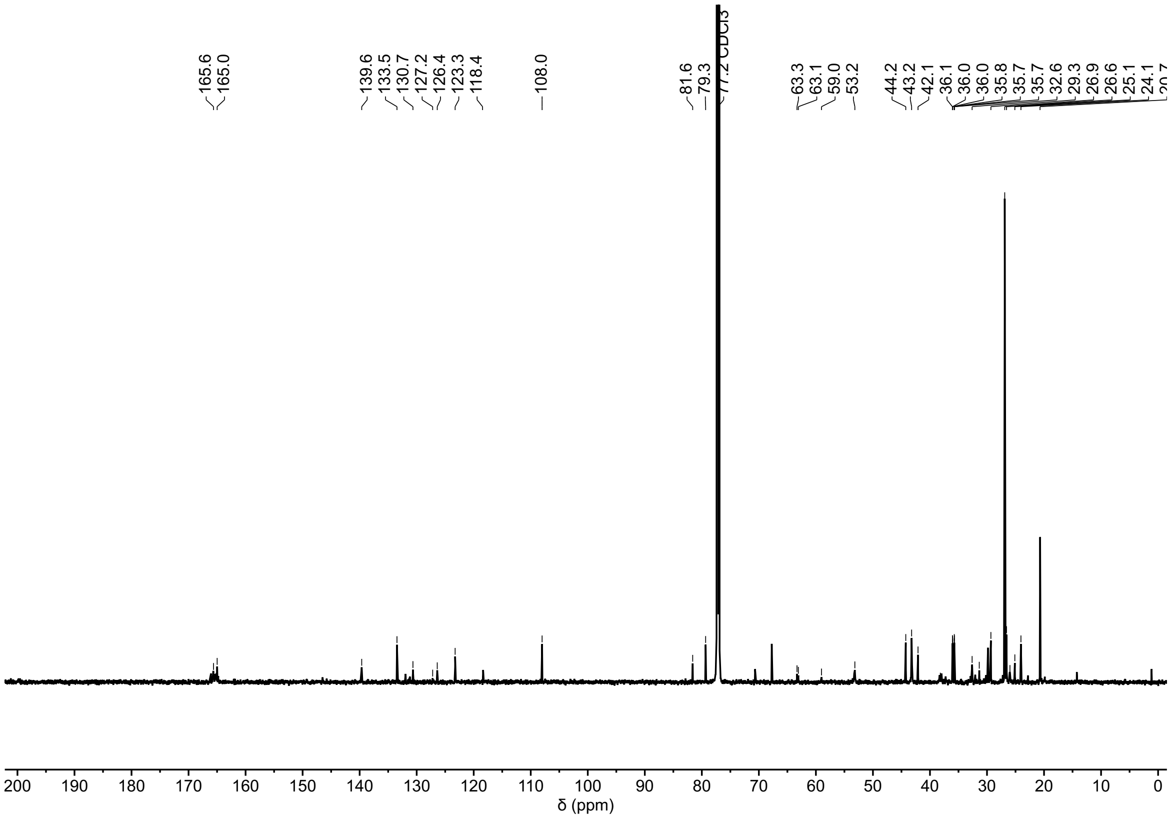

Figure S20. ^{13}C NMR (176 MHz, $CDCl_3$) of the 7-mer template.

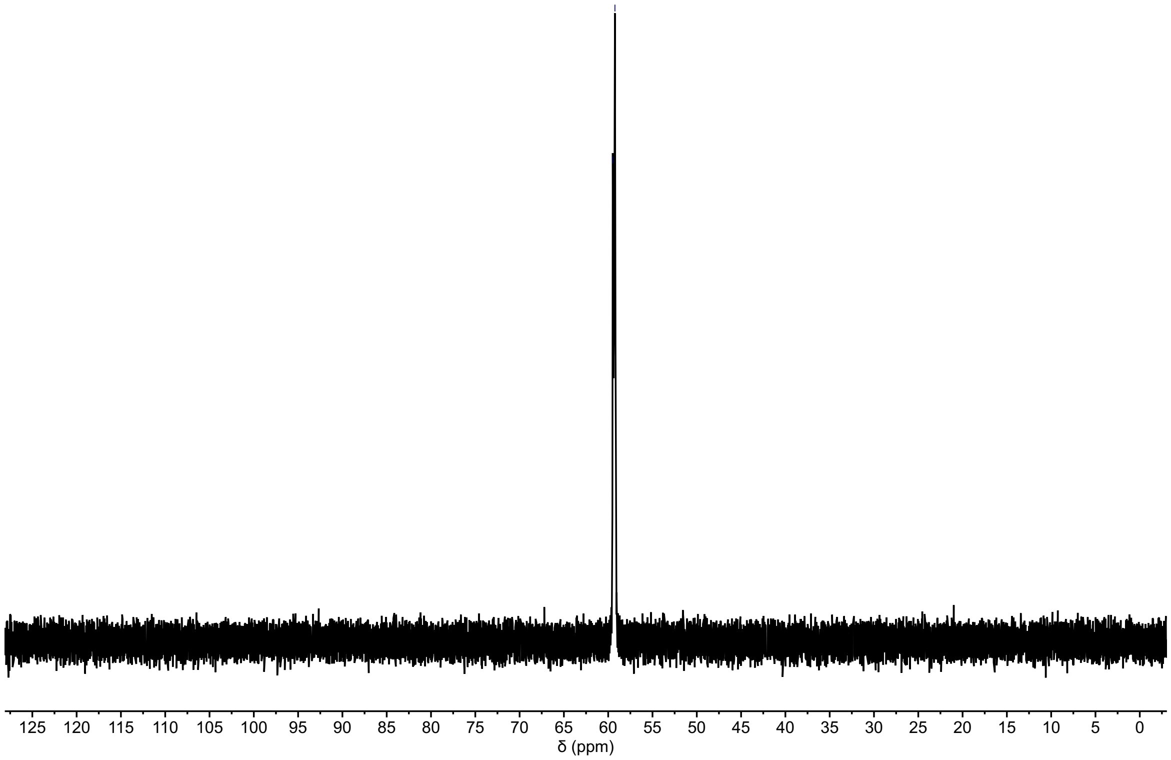

Figure S21. ^{31}P NMR (203 MHz, $CDCl_3$) of the 7-mer template.

2.4. Synthesis and characterization of the 11-mer template

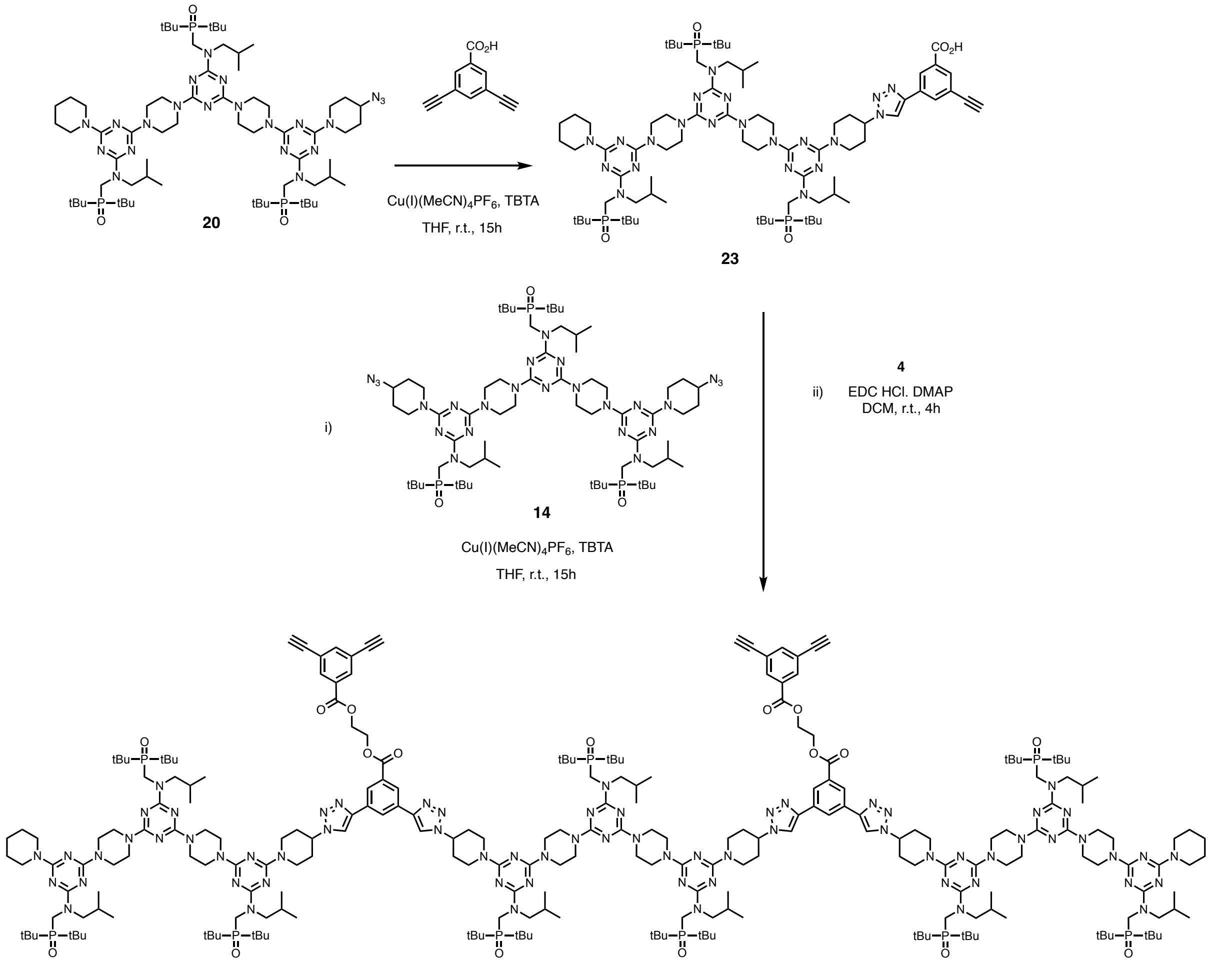

Scheme S4. Synthesis of the 11-mer template.

Synthesis of compound 23

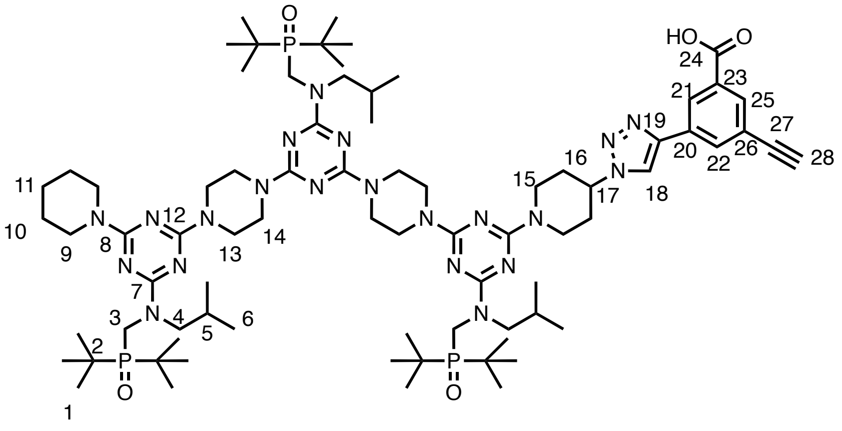

A solution of **20** (150 mg, 0.11 mmol), **3** (75 mg, 0.44 mmol), $[Cu(MeCN)_4]PF_6$ (82 mg, 0.22 mmol) and TBTA (115 mg, 0.22 mmol) in THF (44 mL) was stirred at room temperature for 24 hours and monitored by HPLC. The reaction mixture was diluted by adding EtOAc and the organic phase was washed with basic EDTA/NH_4OH (aq, 0.01 M) (2 x 20 ml), H_2O (2x 20 mL), brine (20 mL) and dried with $MgSO_4$. The solvent was removed under reduced pressure and the crude residue was purified by column chromatography (SiO_2, 0-15 % MeOH in DCM) to afford the product as a white solid (54 mg, 32%).

^{1}H NMR (400 MHz, THF-d_8) δ 8.57-8.44 (m, 2H, C18H-ArH), 8.20 (s, 1H, ArH), 8.02 (s, 1H, ArH), 5.02-4.89 (m, 2H, C15H), 4.88-4.77 (m, 1H, C17H), 4.55-4.35 (s, 6H, C3H_2), 3.95-3.69 (m, 26H, C4H_2-C9H_2-C13H_2-C14H_2), 3.67 (s, 1H, C28H), 3.18-2.97 (m, 2H, C15′H), 2.30-2.19 (m, 5H, C5H-C16H), 2.13-1.92 (m, 2H, C16′H), 1.67-1.59 (m, 2H, C11H_2), 1.53-1.49 (m, 4H, C10H_2), 1.25-1.33 (d, *J* = 12.2 Hz, 54H, C1H_3), 0.92-0-86 (m, 18H, C6H_3).

^{13}C NMR (176 MHz, THF-d_8) δ 167.3-165.5 (C=X), 146.5, 143.3, 133.8, 133.4, 132.9 (ArH), 127.6 (ArH), 124.2, 120.1 (C18), 83.6 (C28), 80.0, 79.7, 79.5, 79.3, 59.5 (C17), 53.8 (C4), 45.0 (C9), 44.1 (C13-C14), 43.0 (C15), 38.7 (d, J = 54.4 Hz, C3) 36.6 (d, J = 55.6 Hz, C2), 33.4 (C16), 27.5 (C5), 27.2 (C1), 26.9 (C10), 26.1 (C11), 21.1 (C6).

^{31}P NMR (203 MHz, THF-d_8) δ 57.57.

HRMS: (ESI$^+$) calculated for $[C_{77}H_{129}N_{21}O_5P_3]^+$: 1520.9698, found $[M+H]^+$: 1520.9733.

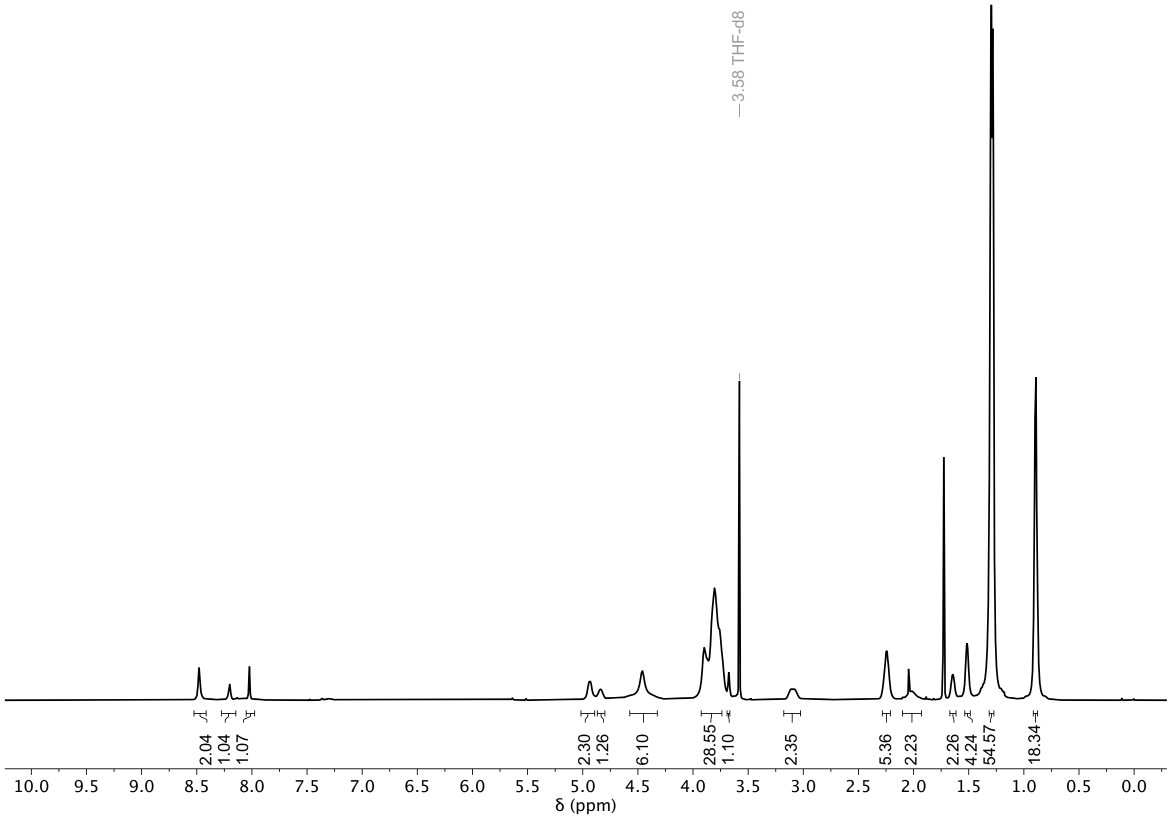

Figure S22. ^{1}H NMR (400 MHz, THF-d_8) of compound **23**.

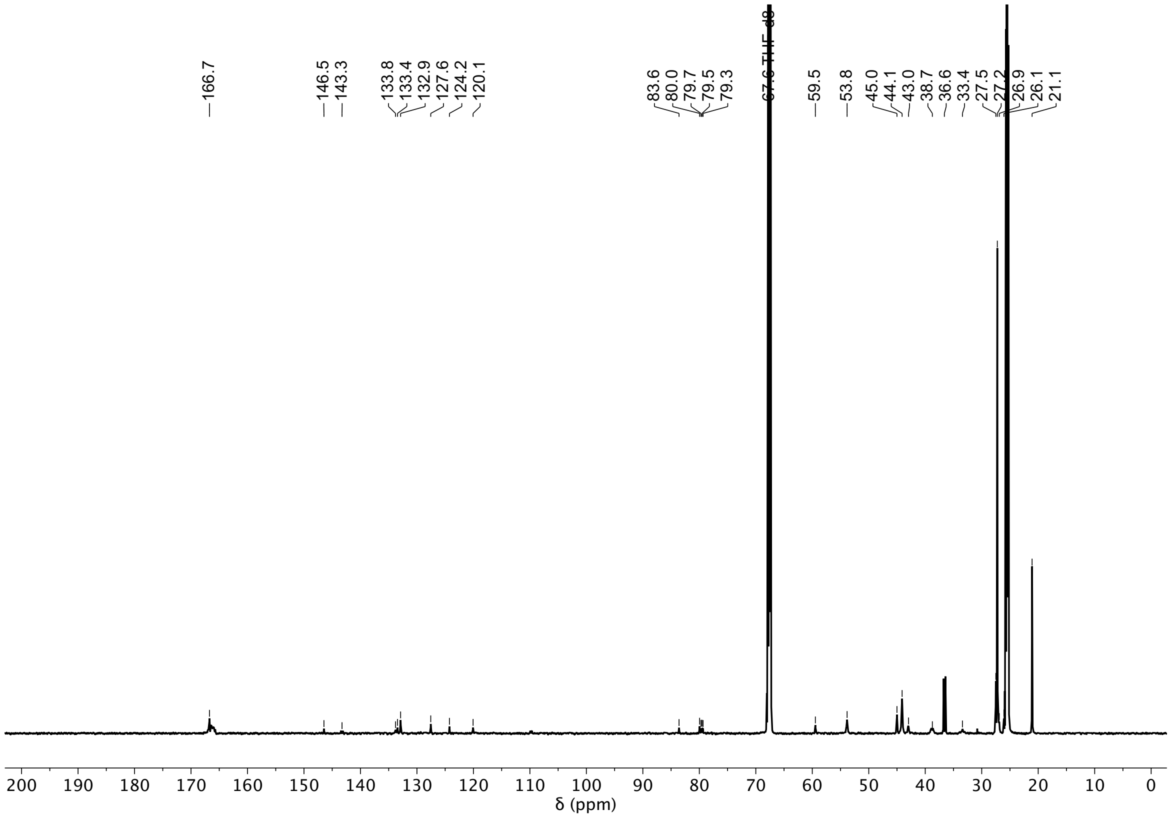

Figure S23. ^{13}C NMR (176 MHz, THF-d_8) of compound **23**.

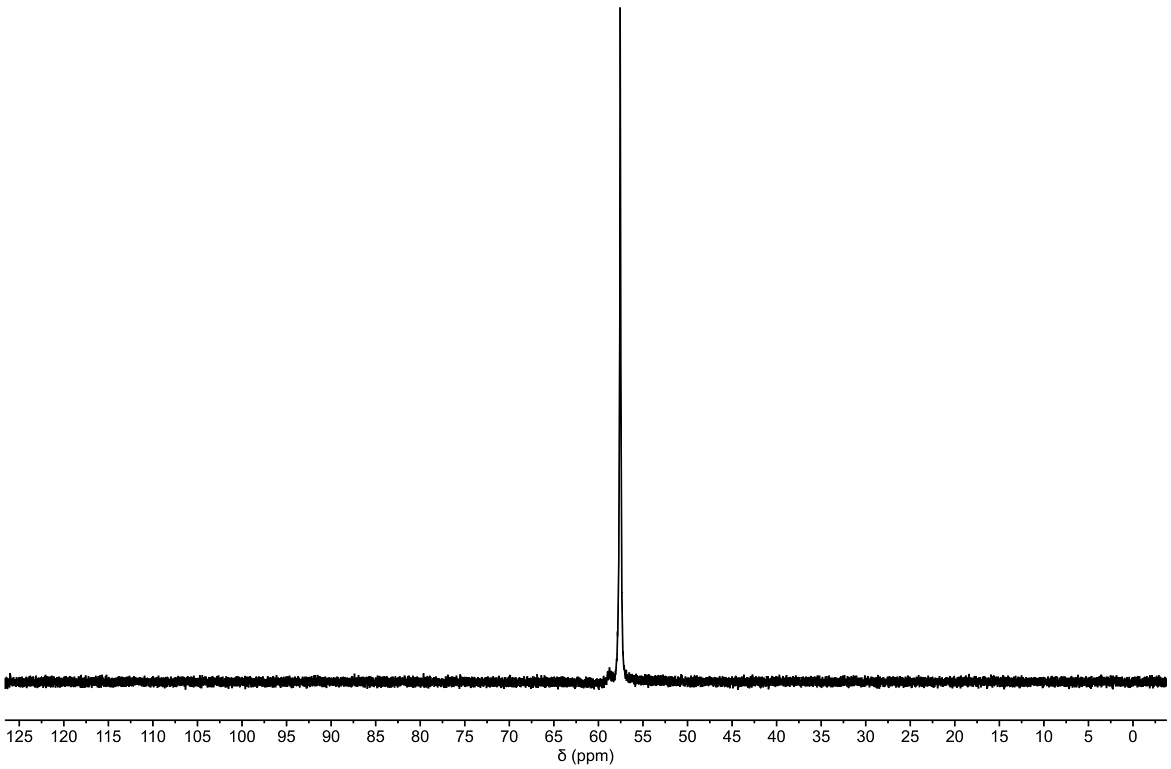

Figure S24. ^{31}P NMR (203 MHz, THF-d_8) of compound **23**.

Synthesis of the 11-mer template

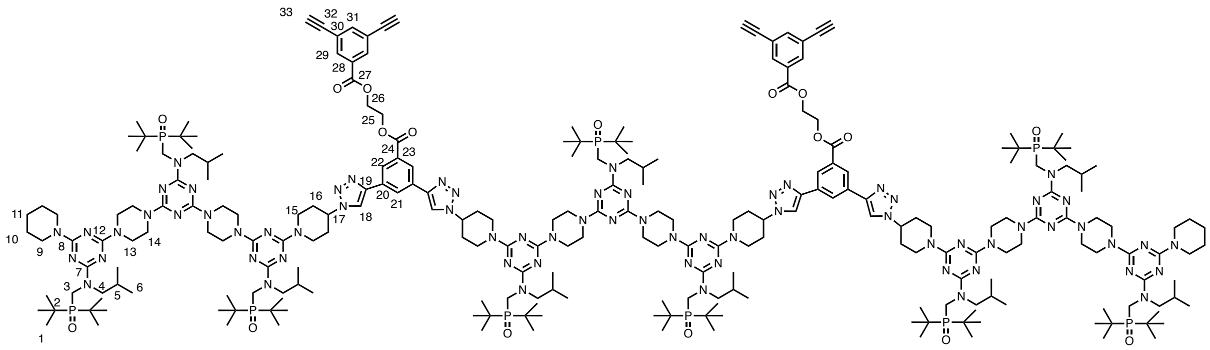

A solution of **23** (50 mg, 0.0329 mmol), **14** (23 mg, 0.016 mmol), $[Cu(MeCN)_4]PF_6$ (12 mg, 0.032 mmol) and TBTA (17 mg, 0.032 mmol) in THF (6.4 mL) was stirred at room temperature for 24 hours and monitored by HPLC. The reaction mixture was diluted by adding EtOAc and the organic phase was washed with basic EDTA/NH_4OH (aq, 0.01 M, 20 ml), H_2O (2 x 20 mL), HCl (0.1 M, 20 mL) and dried with $MgSO_4$. The solvent was removed under reduced pressure and the residue was purified by column chromatography (SiO_2, 0-15 % MeOH in DCM) and used without further purification.

Under an inert N_2 atmosphere, compound **24** (9 mg, 0.073 mmol), EDC (14 mg, 0.073 mg), DMAP (9 mg, 0.0733 mmol) were dissolved in anhydrous DCM (1.5 mL). A solution of compound **4** (65 mg, 0.0147 mmol) in anhydrous DCM (0.5 mL) was added. The reaction mixture was stirred at room temperature for 4h. DCM (10 mL) was added, and the organic phase was washed with EDTA/NH_4OH (aq, 0.01 M) (20 mL), H_2O (2 x 20 mL), brine (20 mL) and dried with $MgSO_4$. The solvent was removed under reduced pressure and the crude compound was purified by preparative HPLC (C8, 5% CH_3CN in H_2O: THF 40:60) to afford the title compound as a white solid (26 mg, 37%).

^{1}H NMR (700 MHz,$CDCl_3$) δ 8.61 (s, 2H, C21H), 8.42 (s, 4H, C22H), 8.10 (s, 4H, C29H), 7.94 (s, 4H, C18H), 7.73 (s, 2H, C31H), 5.00-4.93 (m, 8H, C15H), 4.84-4.73 (m, 4H, C17H), 4.70 (s, 8H, C25H_2-C26H_2), 4.43 (s, 18H, C3H_2), 3.95-3.66 (m, 18H, C4H_2; m, 48H, C13H_2-C14H_2; m, 8H, C9H_2), 3.12 (s, 4H, C33H), 3.11-3.02 (m, 8H, C15′H), 2.36-2.30 (m, 8H, C16H), 2.23-2.18 (m, 9H, C5H), 2.12-1.99 (m, 8H, C16′H), 1.71-1.64 (m, 4H, C11H_2), 1.61-1.52 (m, 8H, C10H_2), 1.33-1.26 (m, 162H, C1H_3), 0.93-0.85 (m, 54H, C6H_3);
^{13}C NMR (176 MHz, $CDCl_3$) δ 166.1 (C=X), 165.7(C=X), 165.4 (C=X), 165.1 (C=X), 165.0 (C=X), 146.5 (C19), 139.6 (C31), 133.5 (C2), 132.0 (ArC), 131.2 (ArC), 130.7 (ArC), 129.8 (ArC), 127.2 (C21), 126.4 (C22), 123.3, 118.3 (C18), 81.6 (C32), 79.3 (C33), 63.3 (C25 or C26), 63.1 (C25 or C26), 59.1 (C17), 53.2 (C4), 44.3 (C9), 43.2 (C13-C14), 42.1 (C15), 38.1 (d, *J* = 56.0 Hz, C3), 35.9 (dm, *J* = 55.2 Hz, C2), 32.6 (C16), 26.9 (C1), 26.6 (C5), 26.0 (C10), 25.1 (C11), 20.7 (C6).
^{31}P NMR (202 MHz, $CDCl_3$) δ 58.9.
HRMS: (ESI^+) calculated for $[C_{246}H_{395}N_{66}O_{17}P_9]^{2+}$: 2413.4884, found $[M+2H]^{2+}$: 2413.4916.

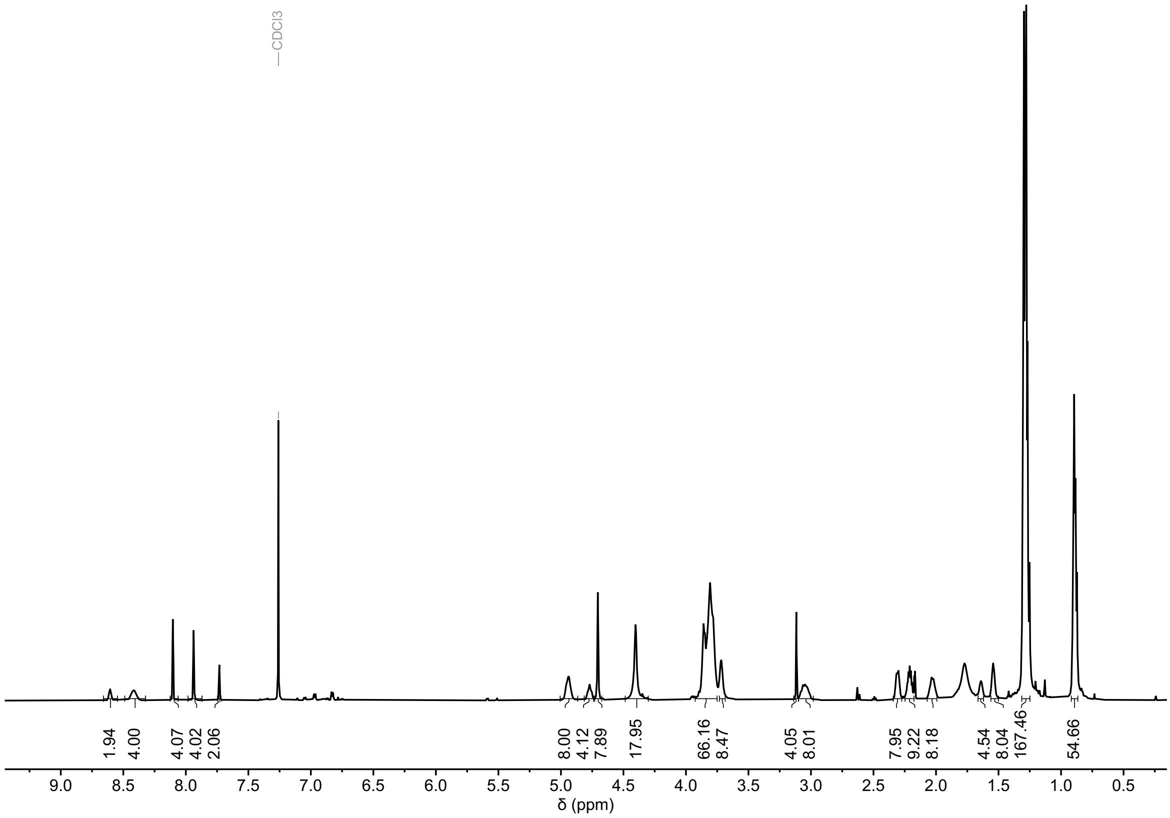

Figure S25. ^{1}H NMR (700 MHz, $CDCl_3$) of the 11-mer template.

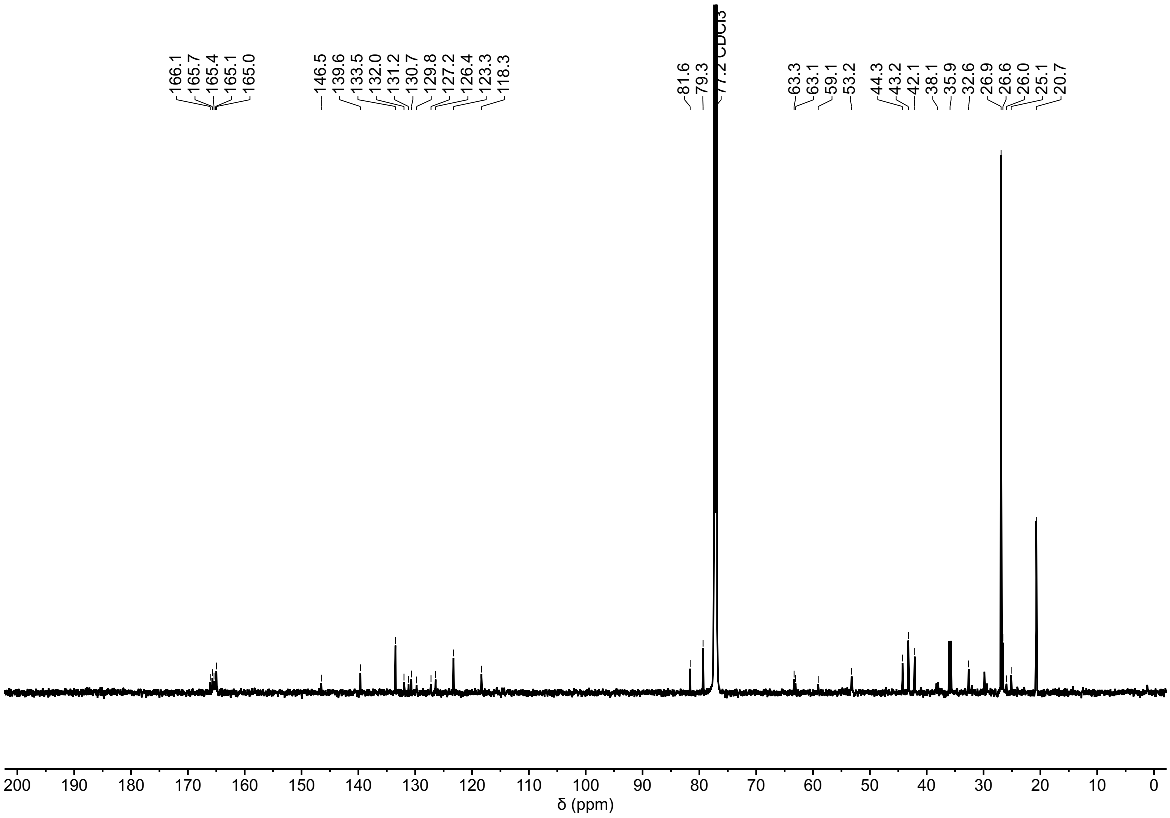

Figure S26. ^{13}C NMR (176 MHz, $CDCl_3$) of the 11-mer template.

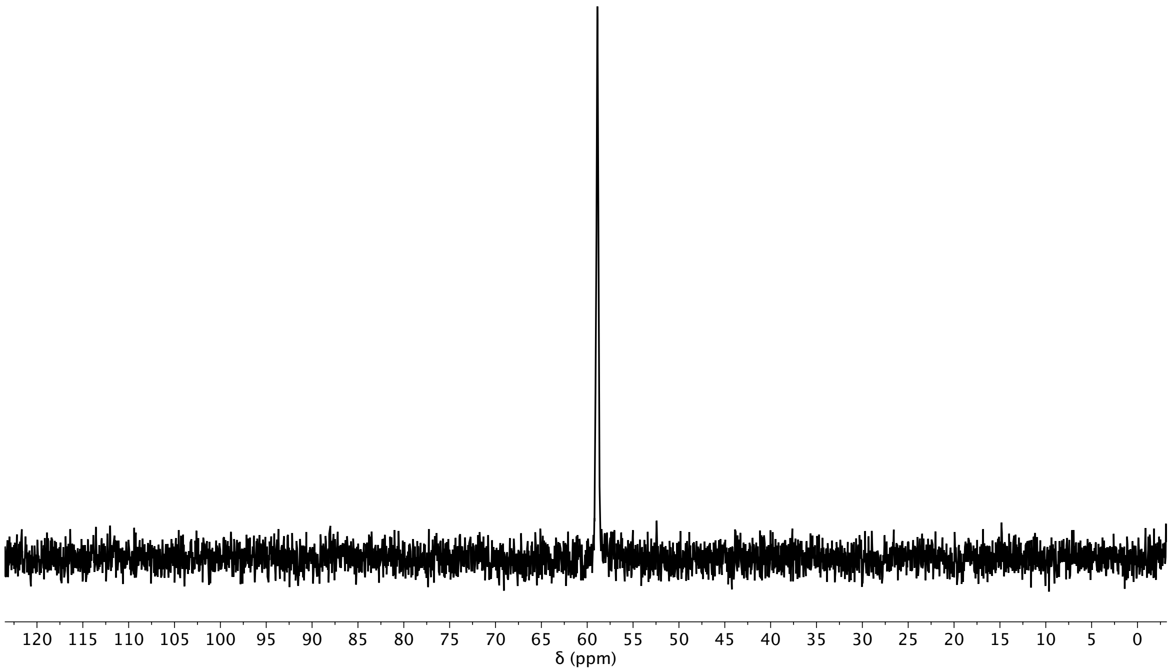

Figure S27. ^{31}P NMR (203 MHz, $CDCl_3$) of the 11-mer template.

2.5. Synthesis and characterization of 4-nitrophenol 3-mer 17

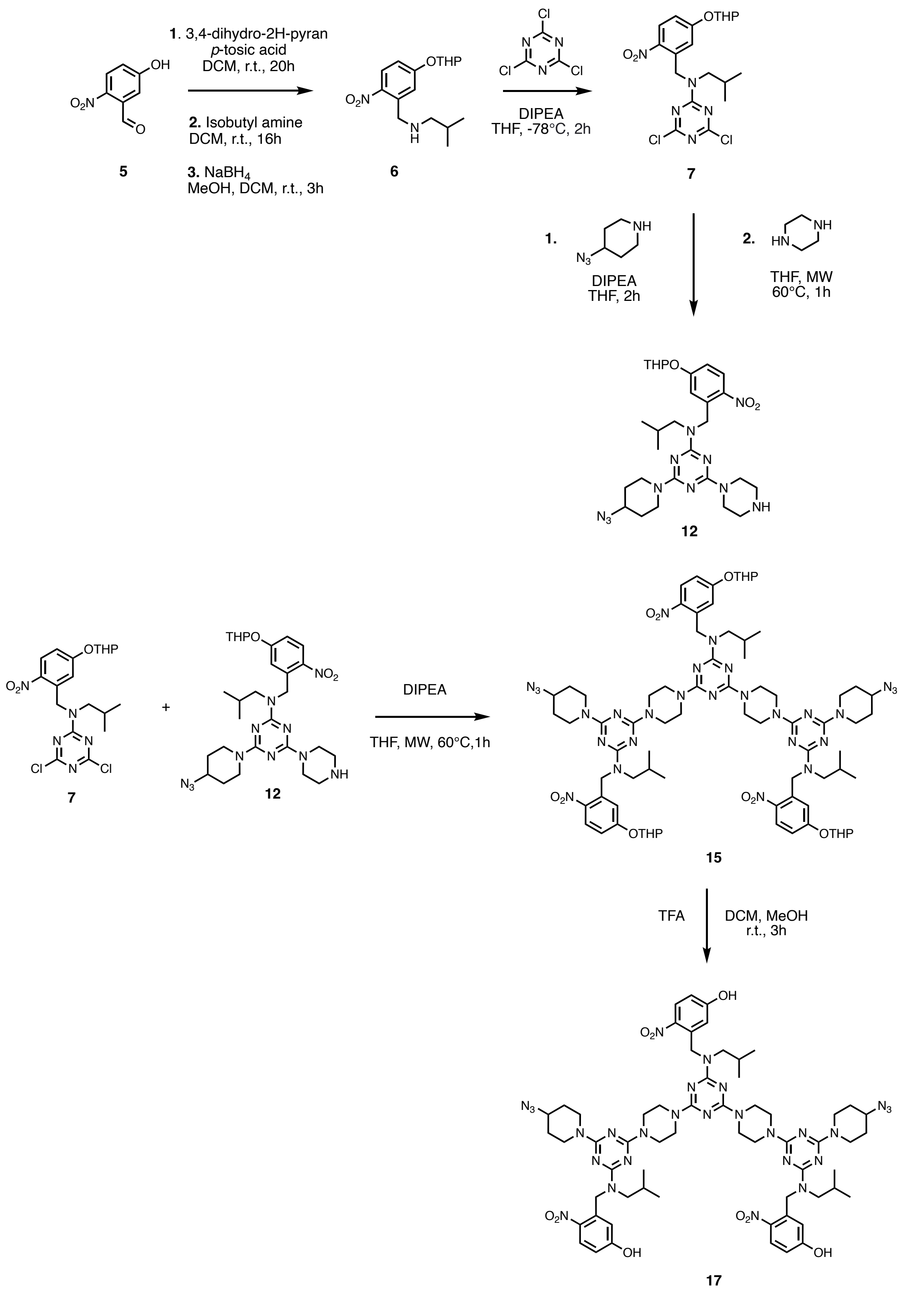

Scheme S5. Synthesis of **zDDDz 17**.

Synthesis of compound 6

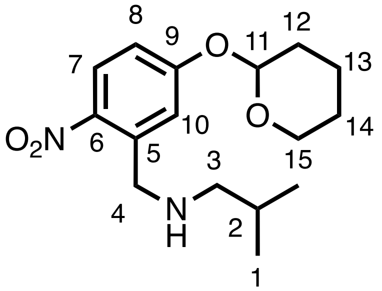

THP protection

Under an inert N_2 atmosphere, 5-hydroxy-2-nitrobenzaldehyde (10.00 g, 59.84 mmol) and *p*-toluenesulfonic acid monohydrate (90 mg, 0.48 mmol) were dissolved in DCM (100 mL). 3,4-dihydro-2H-pyran (27.30 mL, 299.19 mmol) was added dropwise and the resulting reaction mixture was stirred at room temperature for 20 hours. The solution was found to change colour from yellow to pink over 1 hour. If this colour change was not observed, a drop of pyridine was added to help the *p*-toluenesulfonic acid monohydrate dissolve. The volatiles were removed *in vacuo* and the residue was dissolved in DCM (90 mL) and washed with an aqueous K_2CO_3 solution (1M, 50 mL) and brine (2 x 50 mL). The resulting organic phase was dried over $MgSO_4$, and the solvent was removed *in vacuo*. The crude was used for the next step without further purification.

Reductive amination

The aldehyde was dissolved in DCM (100 mL) and isobutyl amine (8.0 mL, 77.79 mmol) and molecular sieves (4 Å) were added. The solution was stirred overnight and monitored by ^{1}H NMR. The molecular sieves were removed by filtration and the solvent was removed *in vacuo* to obtain a yellow oil.
MeOH (40 mL) was added, and the solution was cooled to 0°C. $NaBH_4$ (4.53 g, 119.67 mmol) was added in small portions with vigorous stirring. Once the addition was complete, the reaction mixture was stirred for 3 hours and monitored ^{1}H NMR. Upon completion, the solvent was removed *in vacuo* and the residue was dissolved in aqueous NaOH (1M, 100 mL), extracted with DCM (3 x 50 mL) and dried with $MgSO_4$. The solvent was removed *in vacuo* to afford the crude product. The obtained residue was purified by flash chromatography (SiO_2, 0–10% MeOH in DCM) to yield the product as a yellow oil (13.83 g, 44.88 mmol, 75%).

^{1}H NMR (500 MHz, $CDCl_3$) δ 8.05 (d, *J* = 9.0 Hz, 1H, C7H), 7.30 (d, *J* = 2.7 Hz, 1H, C10H), 7.01 (dd, *J* = 9.1, 2.7 Hz, 1H, C8H), 5.54 (t, *J* = 3.1 Hz, 1H, C11H), 4.06 (ABq, J_{AB} = 15.0 Hz, $\Delta\delta_{AB}$ = 0.02, 2H, C4H_2), 3.82 (ddd, *J* = 11.4, 10.1, 3.0 Hz, 1H, C15H), 3.63 (dtd, *J* = 11.4, 4.0, 1.4 Hz, 1H, C15′H), 2.46 (dd, *J* = 6.8, 2.0 Hz, 2H, C3H_2), 2.05 – 1.94 (m, 1H, C13H), 1.92 – 1.85 (m, 2H, C12H_2), 1.84 – 1.73 (m, 1H, C2H), 1.75 – 1.66 (m, 2H, C13′H-C14H), 1.65 – 1.57 (m, 1H, C14′H), 0.93 (d, *J* = 6.7 Hz, 6H, C1H_3).

^{13}C NMR (126 MHz, $CDCl_3$) δ 161.2 (C9), 142.4 (C5), 139.3 (C6), 127.7 (C7), 118.3 (C10), 114.8 (C8), 96.4 (C11), 62.2 (C15), 57.7 (C3), 51.7 (C4), 30.1 (C12), 28.6 (C2), 25.1 (C14), 20.8 (C1), 18.4 (C13).

HRMS: (ESI^+) calculated for $[C_{16}H_{25}N_2O_4]^+$: 309.1814, found $[M+H]^+$: 309.1817.

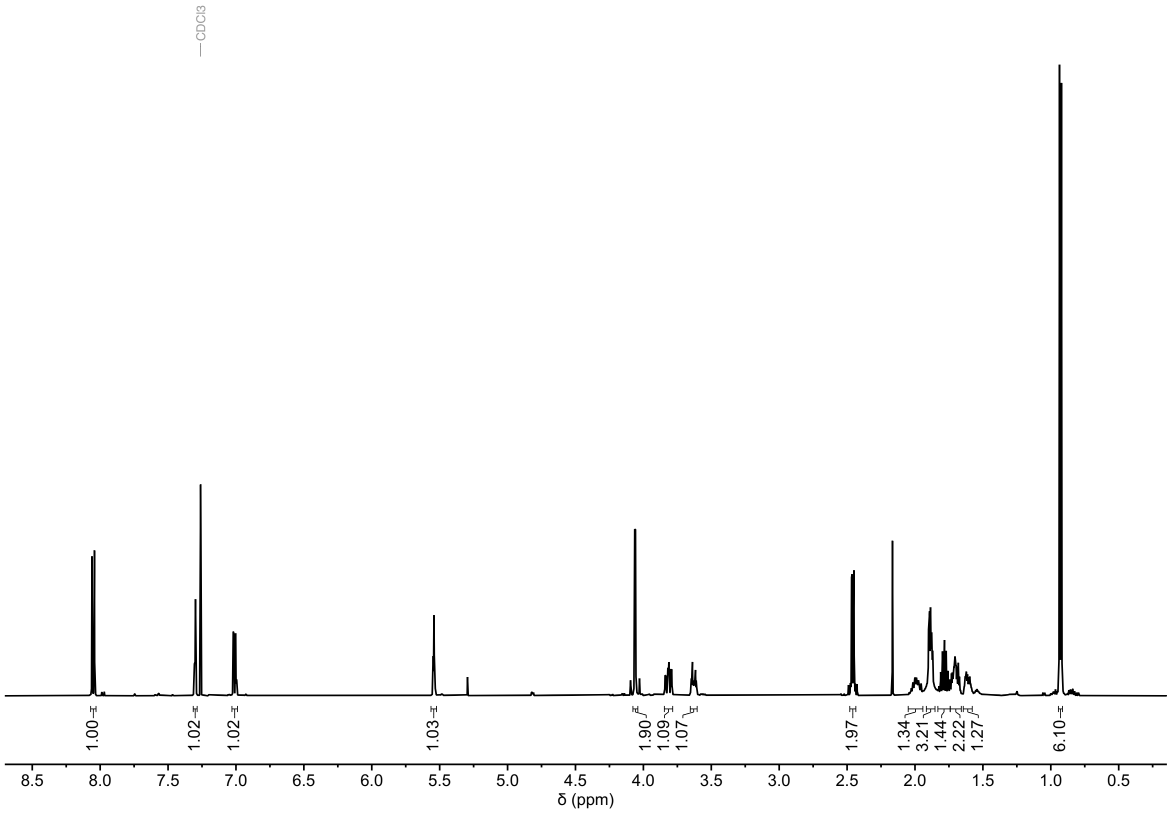

Figure S28. ^{1}H NMR (500 MHz, $CDCl_3$) of compound **6**.

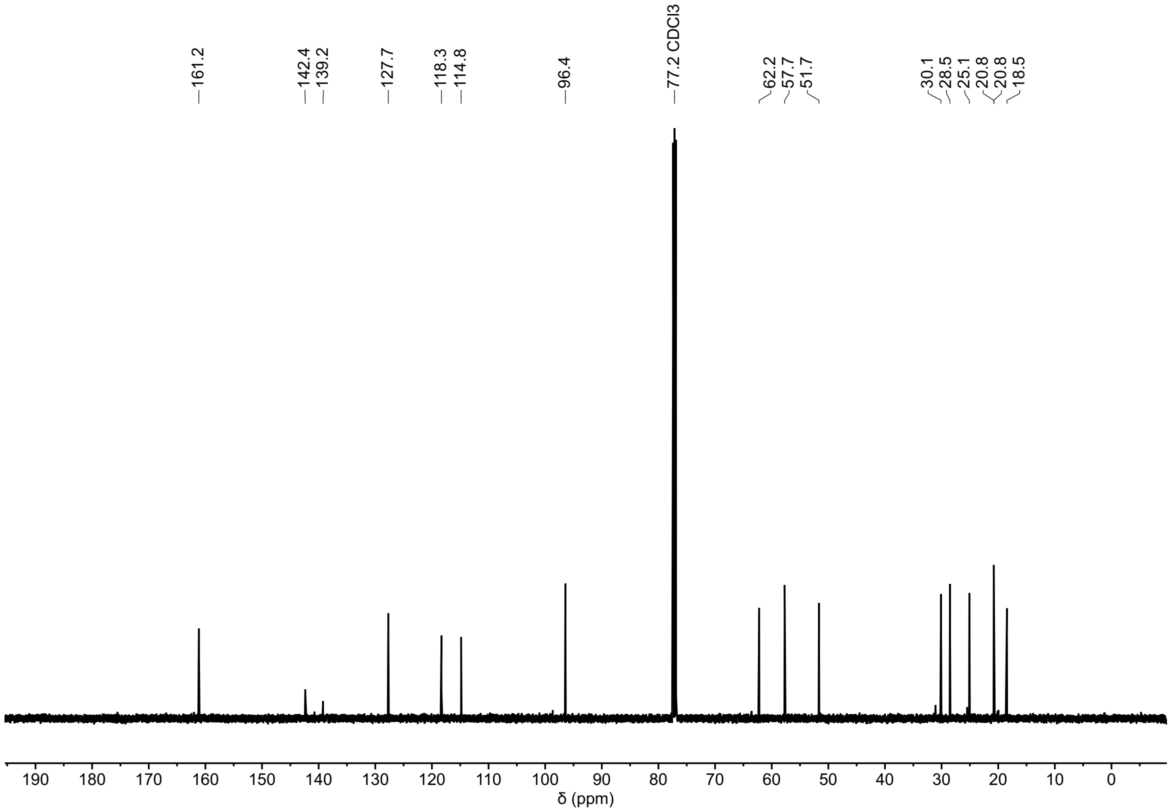

Figure S29. ^{13}C NMR (126 MHz, $CDCl_3$) of compound **6**.

Synthesis of compound 7

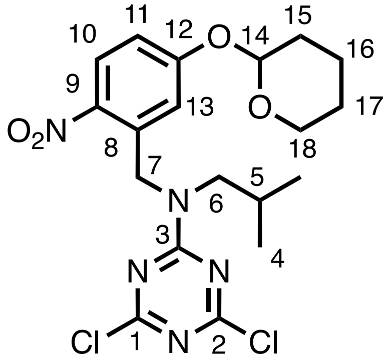

A solution of **6** (1.687 g, 5.47 mmol) and DIPEA (1.91 mL, 1.413 g, 10.93 mmol) in THF (5 mL) was added to a solution of cyanuric chloride (1.000 g, 5.47 mmol) in THF (5 mL) at -78°C. The resulting solution was stirred at -78°C for 2 hours and the reaction progress was monitored by HPLC. The solution was diluted with EtOAc (30 mL), washed with H_2O (3 x 30 mL) and dried with $MgSO_4$. The solvent was removed *in vacuo* to afford the crude product. The obtained residue was purified by flash chromatography (SiO_2, 0-8% EtOAc in petroleum ether 40-60) to afford compound **7** as white needle like crystals (2.1327 g, 4.686 mmol, 86%).

^{1}H NMR (500 MHz, $CDCl_3$) δ 8.21 (d, *J* = 9.1 Hz, 1H, C10H), 7.08 (dd, *J* = 9.1, 2.6 Hz, 1H, C11H), 6.65 (d, *J* = 2.6 Hz, 1H, C13H), 5.42 (t, *J* = 3.2 Hz, 1H, C14H), 5.27 (ABq, J_{AB} = 18.0 Hz, $\Delta\delta_{AB}$ = 0.02, 2H, C7H_2), 3.75 (ddd, *J* = 11.3, 9.8, 3.1 Hz, 1H, C18H), 3.56 (dtd, *J* = 11.4, 4.0, 1.4 Hz, 1H, C18′H), 3.51 (m, 2H, C6H_2), 2.23 – 2.11 (m, 1H, C5H), 2.02-1.93 (m, 1H, C16H), 1.87 (ddd, *J* = 7.6, 4.6, 3.2 Hz, 2H, C15H_2), 1.74-1.64 (m, 2H, C16′H-C17H), 1.64 – 1.58 (m, 1H, C17′H), 1.00-0.92 (m, 6H, C4H_3).

^{13}C NMR (126 MHz, $CDCl_3$) δ 170.6 (C1 or C2), 170.4 (C1 or C2), 166.13 (C3), 161.9 (C12), 141.5 (C8), 134.8 (C9), 128.7 (C10), 115.1 (C13), 115.0 (C11), 97.0 (C14), 62.4 (C18), 55.4 (C6), 49.6 (C7), 30.0 (C15), 27.1 (C5), 24.9 (C17), 20.2 (C4), 20.2 (C4′), 18.5 (C16).

HRMS: (ESI^+) calculated for $[C_{19}H_{23}Cl_2N_5O_4Na]^+$: 516.355, found $[M+Na]^+$: 516.3557.

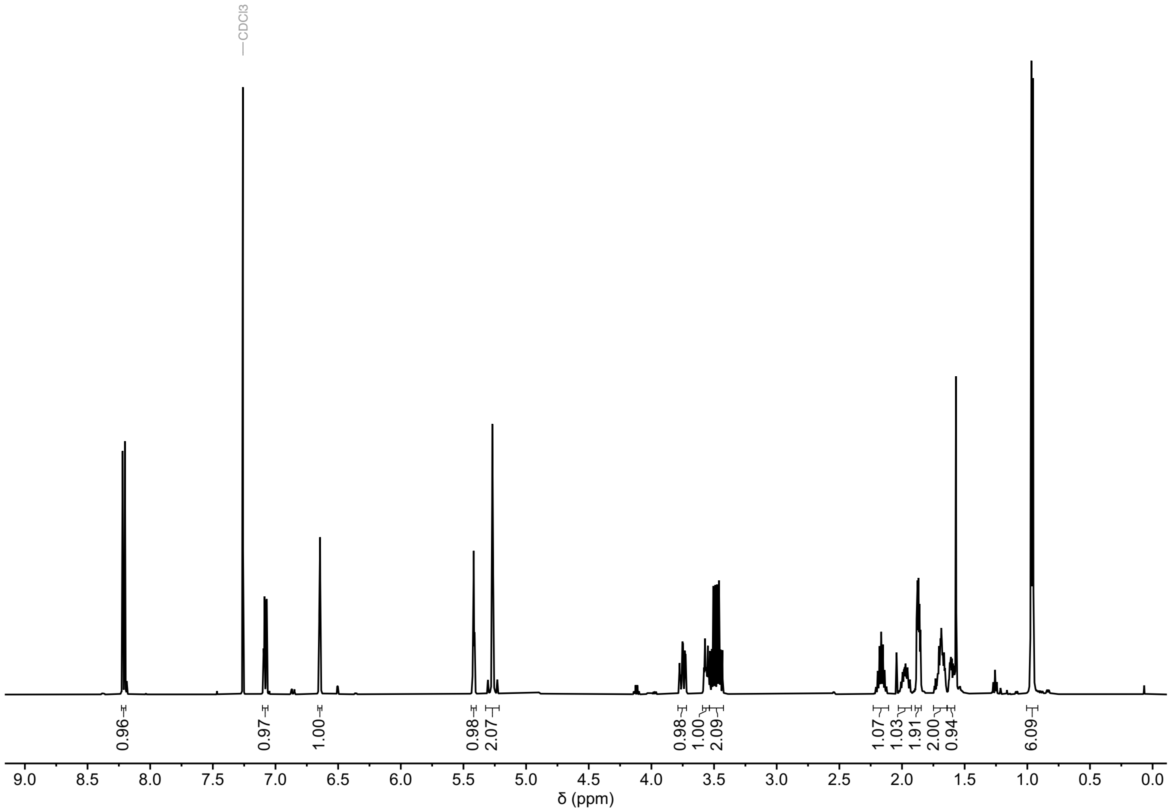

Figure S30. ^{1}H NMR (500 MHz, $CDCl_3$) of compound **7**.

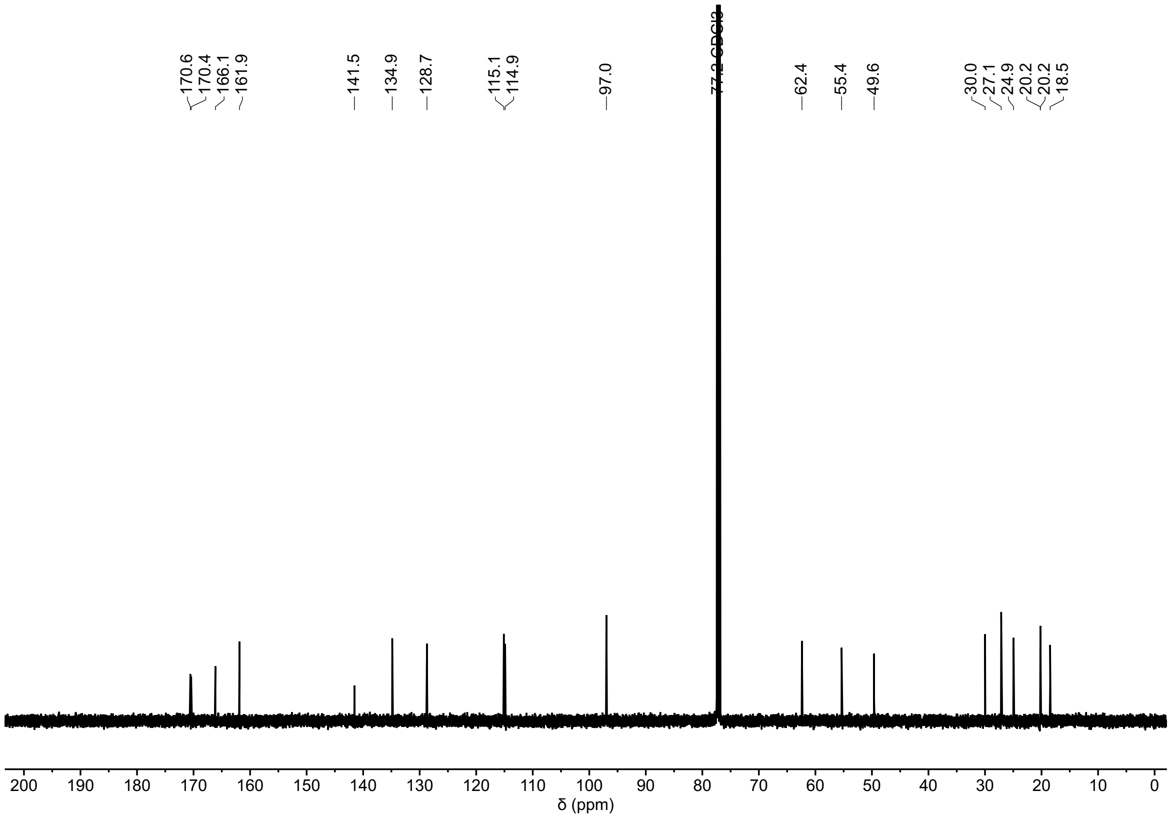

Figure S31. ${}^{13}C$ NMR (126 MHz, $CDCl_3$) of compound **7**.

Synthesis of compound 12

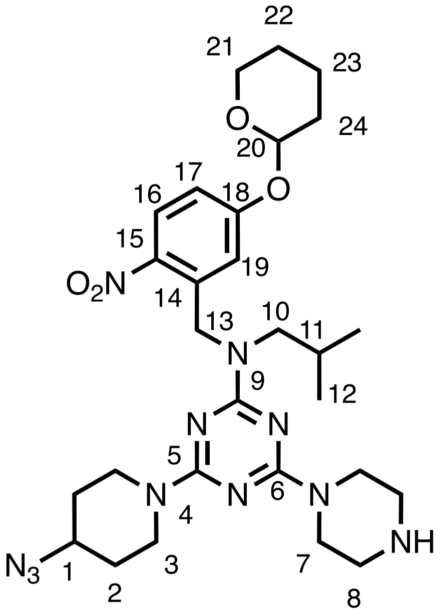

DIPEA (0.350 mL, 1.99 mmol) was added to a solution of 4-azidopiperidine (TFA salt) (0.66 mmol) in THF (3 mL). The resulting solution was added to a solution of **7** (301.9 mg, 0.66 mmol) in THF (3 mL) at 0°C. The resulting mixture was stirred at room temperature for 2 hours and monitored by HPLC. The crude reaction mixture was used in the next step without further purification.

A saturated solution of piperazine (0.8092 g, 9.31 mmols) in THF (5 mL) was sonicated for 10 minutes. From this stock, the supernatant was transferred into the reaction mixture at 0°C. The resulting solution was then stirred in a microwave at 60°C for 1 hour. EtOAc (30 mL) was added to the solution and the organic layer was washed with H_2O (3 x 30 mL) and dried with $MgSO_4$. The solvent was removed *in vacuo* to afford the crude product. The obtained residue was purified by flash chromatography (SiO_2, 0-10 % MeOH in DCM) to afford compound **12** (167.9 mg, 0.290 mmols, 44%) as an orange oil. The NMR spectra are consistent with the presence of slowly exchanging rotamers in solution.

^{1}H NMR (500 MHz, $CDCl_3$) δ 8.08 (d, *J* = 9.3 Hz, 1H, C16H), 7.02 – 6.92 (m, 1H, C17H), 6.82 (d, *J* = 2.7 Hz, 1H, C19H), 5.37 (t, *J* = 3.3 Hz, 1H, C20H), 5.10 (d, *J* = 9.2 Hz, 2H, C13H_2), 4.41-4.33 (m, 1H, C3H), 4.25-4.02 (m, 1H, C3′H), 3.81-3.71 (m, 2H, C21H), 3.67 – 3.60 (m, 1H, C1H), 3.60 – 3.45 (m, 3H, C21′H-C1H), 3.45-3.37 (m, 2H, C10H_2), 3.32-3.24 (m, 1H, C3H), 3.04 (br, 1H, C3′H), 2.90 (m, 2H, C8H), 2.75 (m, 2H, C8′H), 2.12 (m, 1H, C12H), 2.02 – 1.87 (m, 2H, C23H-C2H), 1.87-1.81 (m, 2H, C22H_2), 1.80-1.74 (br, 1H, C2′H), 1.70-1.61 (m, 2H, C23H′-C24H), 1.62 – 1.53 (m, 2H, C24H′-C2H), 1.38 (br, 1H, C2H) 0.92 (d, *J* = 6.7 Hz, 6H, C12H_3).

^{13}C NMR (126 MHz, $CDCl_3$) δ 166.3 (C=N), 165.6 (C=N), 161.5 (C18), 141.9 (C14), 139.1 (C15), 127.6 (C16), 116.1 (C19), 113.8 (C17), 96.8 (C20), 62.5 (C21), 58.3 (C1), 54.9 (C10), 48.9 (C13), 46.1 (C8), 46.0 (C8), 44.4 (C7), 44.3 (C7), 41.0 (C3), 30.7 (C2), 30.6 (C2), 30.1 (C22), 27.8 (C11), 25.0 (C24), 20.7 (C12), 18.8 (C23).

HRMS: (ESI^+) calculated for $[C_{28}H_{42}N_{11}O_4]^+$: 596.3434, found $[M+H]^+$: 596.3421.

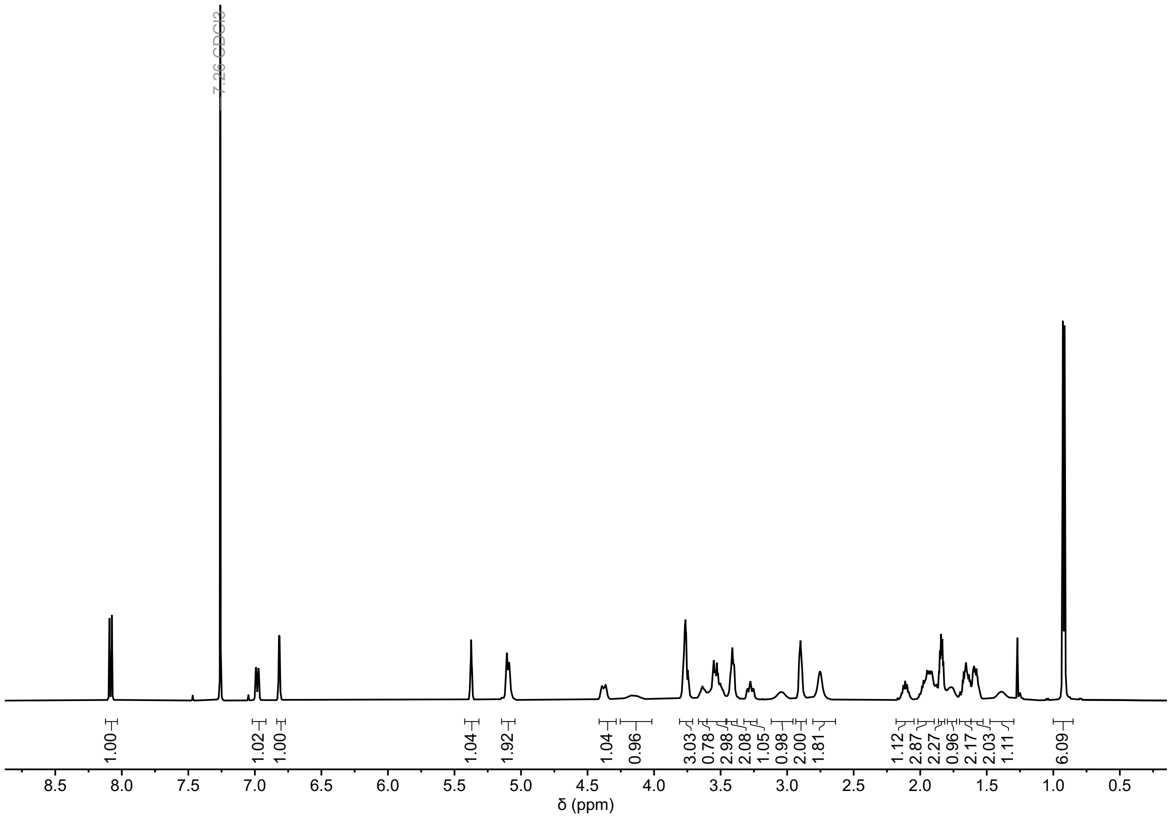

Figure S32. ^{1}H NMR (500 MHz, $CDCl_3$) of compound **12**.

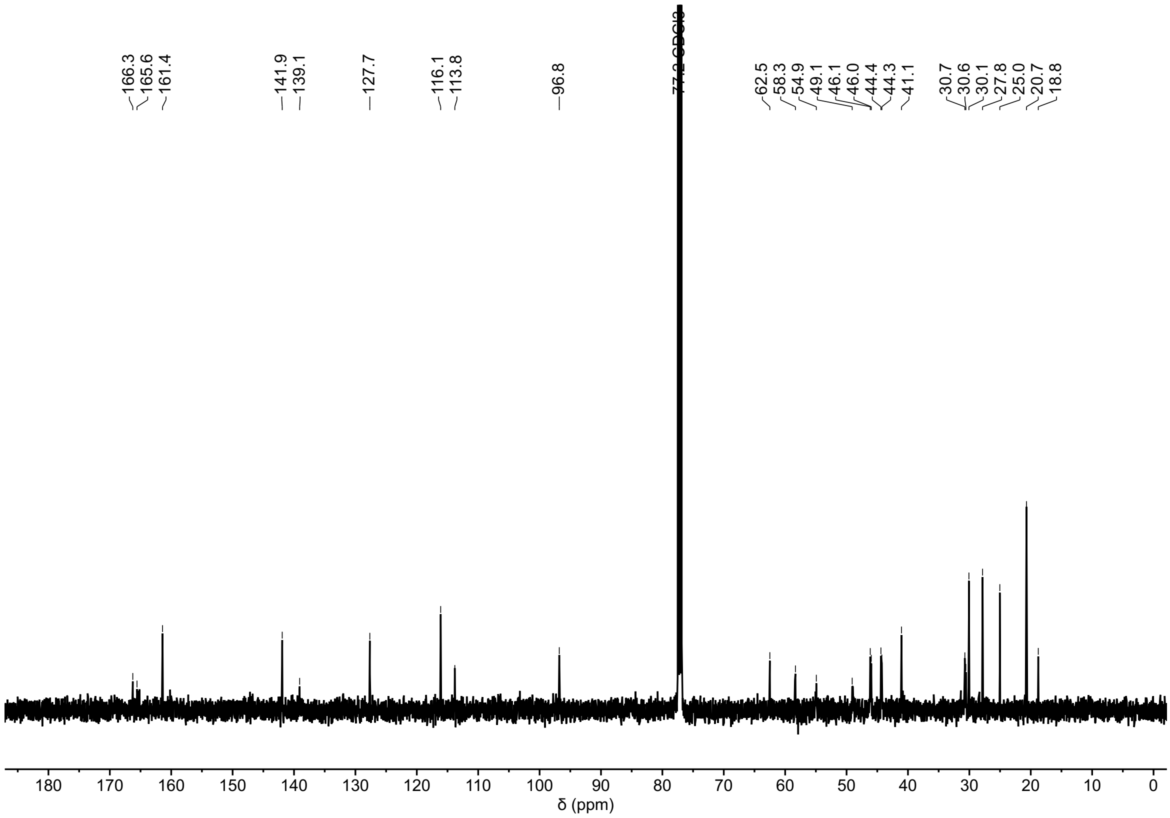

Figure S33. ^{13}C NMR (126 MHz, $CDCl_3$) of compound **12**.

Synthesis of compound 15

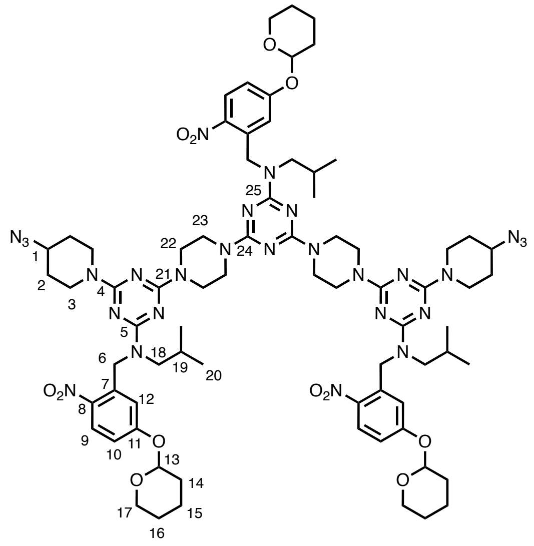

A solution of **7** (33.8 mg, 74.3 µmol), **12** (194.5 mg, 326.8 µmol) and DIPEA (25.9 µL, 19.2 mg, 148.5 µmol) in THF (0.75 mL) was microwaved at 60°C for 1 hour. EtOAc (30 mL) was added to the solution and the organic layer was washed with H_2O (3 x30 mL) and dried with $MgSO_4$. The solvent was removed *in vacuo* to afford the crude product. The obtained residue was purified by flash chromatography (SiO_2, 0-10 % MeOH in DCM) to afford compound **15** (9.4 mg, 6.0 µmol, 8%) as an orange foam. The NMR spectra are consistent with the presence of slowly exchanging rotamers in solution.

^{1}H NMR (400 MHz, $CDCl_3$) δ 8.17-8.02 (m, 3H, C9H), 7.04-6.93 (m, 3H, C10H), 6.87 – 6.74 (m, 3H, C12H), 5.43-5.31 (m, 3H, C13H), 5.22-5.04 (m, 6H, C6H_2), 4.45-4.33 (m, 2H, C3H), 4.28 – 3.99 (m, 2H, C3H), 3.84 (m, 4H, C17H-C22H_2 or C23H_2), 3.79 – 3.70 (m, 6H, C1H-C22H_2 or C23H_2), 3.71 – 3.59 (m, 6H, C22H_2 or C23H_2), 3.58-3.48 (m, 3H, C17H-C1H), 3.46-3.36 (m, 6H, C18H_2), 3.45-3.22 (m, 2H, C3H), 3.16 -2.99 (m, 2H, C3H), 2.15 – 2.04 (m, 3H, C19H), 2.00 – 1.88 (m, 5H, C15H-C2H), 1.87-1.76 (m, 6H, C14H_2), 1.70 – 1.47 (m, 12H, C15′H-C16H_2-C2′H), 0.99-0.86 (m, 18H, C20H_3).

^{13}C NMR (101 MHz, $CDCl_3$) δ 164.7 (C=N), 143.0 (C7), 139.0 (C8), 127.7 (C9), 116.8 (C12), 112.6 (C10), 96.8 (C13), 62.5 (C17), 58.3 (C1), 55.0 (C18), 48.9 (C6), 43.8 (C22 or C23), 41.5 (C3), 30.7 (C2), 30.1 (C14), 27.8 (C19), 25.0 (C16), 20.7 (C20), 19.4 (C15).

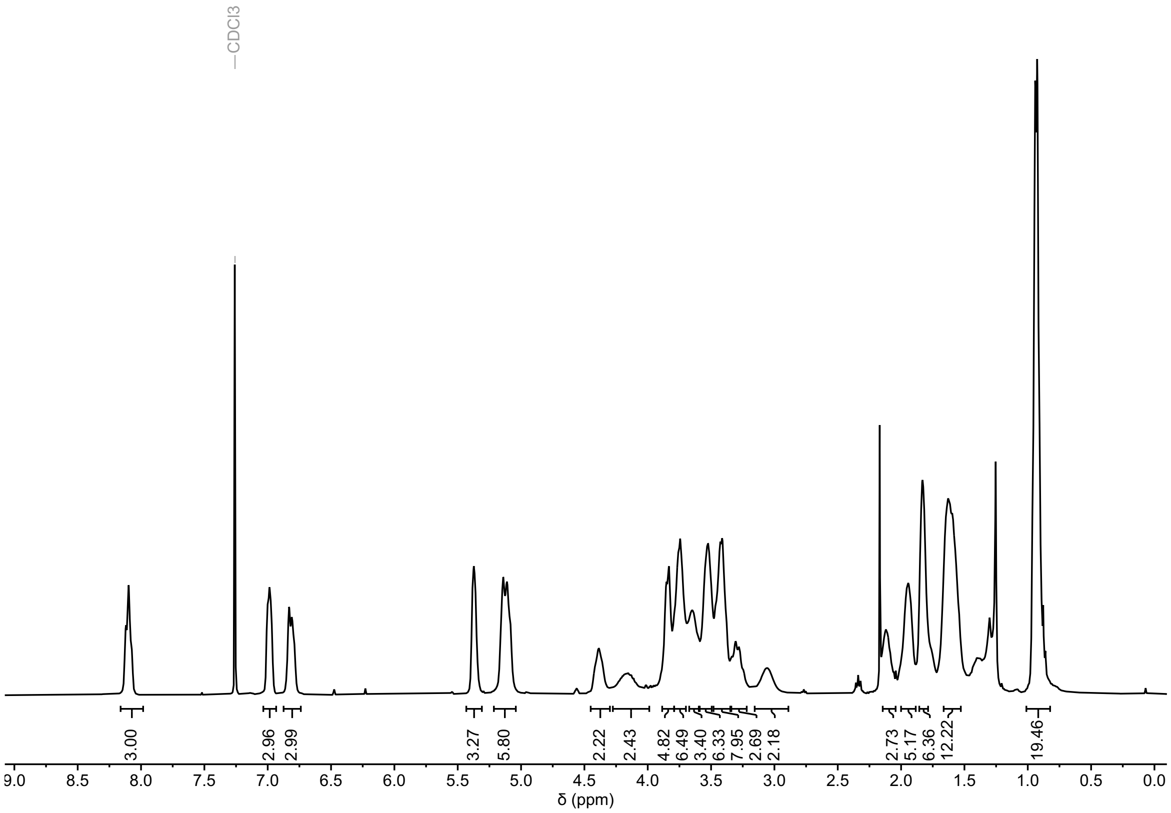

Figure S34. ^{1}H NMR (400 MHz, $CDCl_3$) of compound **15.**

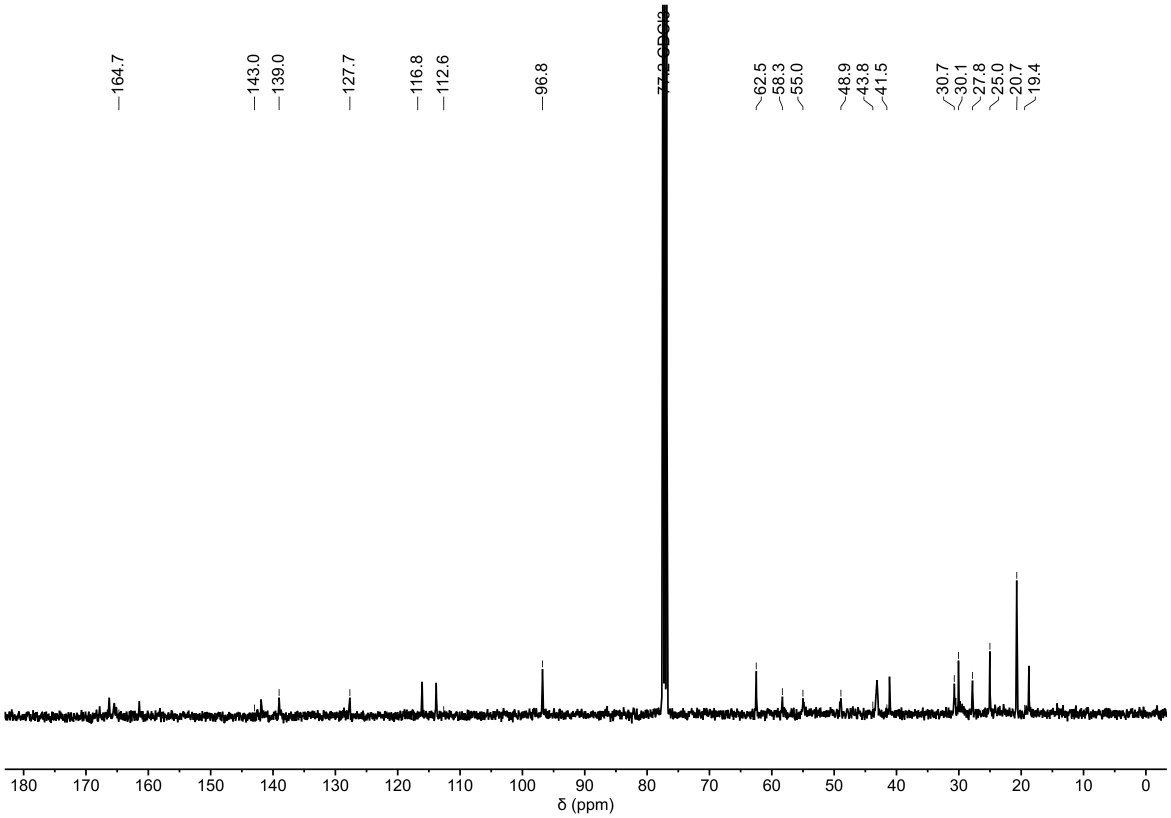

Figure S35. ^{13}C NMR (101 MHz, $CDCl_3$) of compound **15.**

Synthesis of compound 17

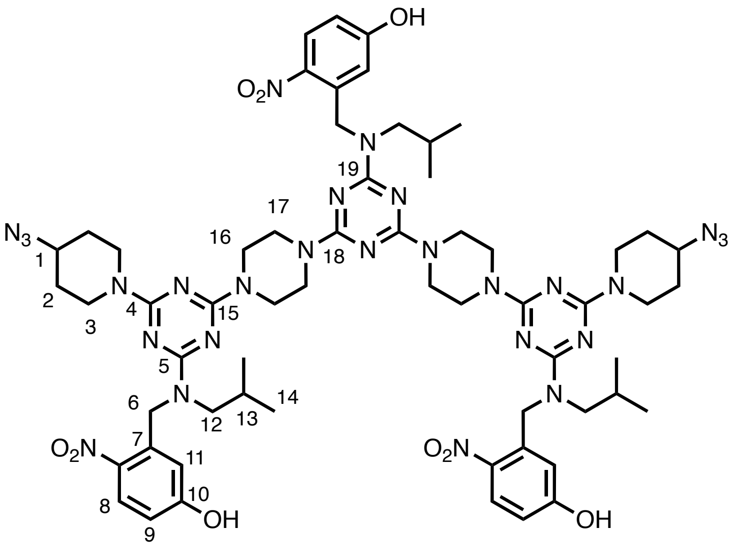

TFA (0.543 mL, 7.10 mmol) was added to a solution of **15** (530 mg, 0.355 mmol) in DCM (0.1 mL) and MeOH (0.1 mL). The solution was stirred for 3 hours and monitored by HPLC. The solvent was removed in vacuo and the obtained residue was purified by flash chromatography (SiO_2, 0-20 % EtOAc in DCM) to afford compound **17** (305 mg, 65%) as a yellow solid. The NMR spectra are consistent with the presence of slowly exchanging rotamers in solution.

^{1}H NMR (700 MHz, $CDCl_3$) δ 8.15-7.96 (m, 3H, C8<u>H</u>), 6.73-6.28 (m, 6H, C9<u>H</u>-C11<u>H</u>), 5.20-5.01 (m, 6H, C6<u>H</u>$_2$), 4.42-4.26 (m, 2H, C3<u>H</u>), 4.22-3.96 (m, 2H, C3<u>H</u>), 3.92-3.22 (m, 24H, C12<u>H</u>$_2$-C3<u>H</u>-C1<u>H</u>-C16<u>H</u>-C17<u>H</u>), 3.17-2.99 (m, 2H, C3<u>H</u>), 2.17-2.05 (m, 3H, C13<u>H</u>), 1.99-1.88 (m, 2H, C2<u>H</u>), 1.84-1.72 (m, 2H, C2<u>H</u>), 1.64-1.49 (m, 2H, C2<u>H</u>), 1.44-1.32 (m, 2H, C2<u>H</u>), 0.98-0.87 (m, 18H, C14<u>H</u>$_3$).

^{13}C NMR (176 MHz, $CDCl_3$) δ 166.4-164.9 (C=N), 161.3, 141.0, 139.6, 128.4 (C8), 114.7 (C9 or C11), 113.9 (C9 or C11), 58.2 (C1), 55.0 (C12), 49.1 (C6), 43.1 (C16-C17), 41.1 (C3), 30.7 (C2), 30.5 (C2), 27.8 (C13), 20.7 (C14)

HRMS: (ESI$^+$) calculated for $[C_{60}H_{80}N_{27}O_9]^+$: 1322.6627, found $[M+H]^+$: 1322.6674.

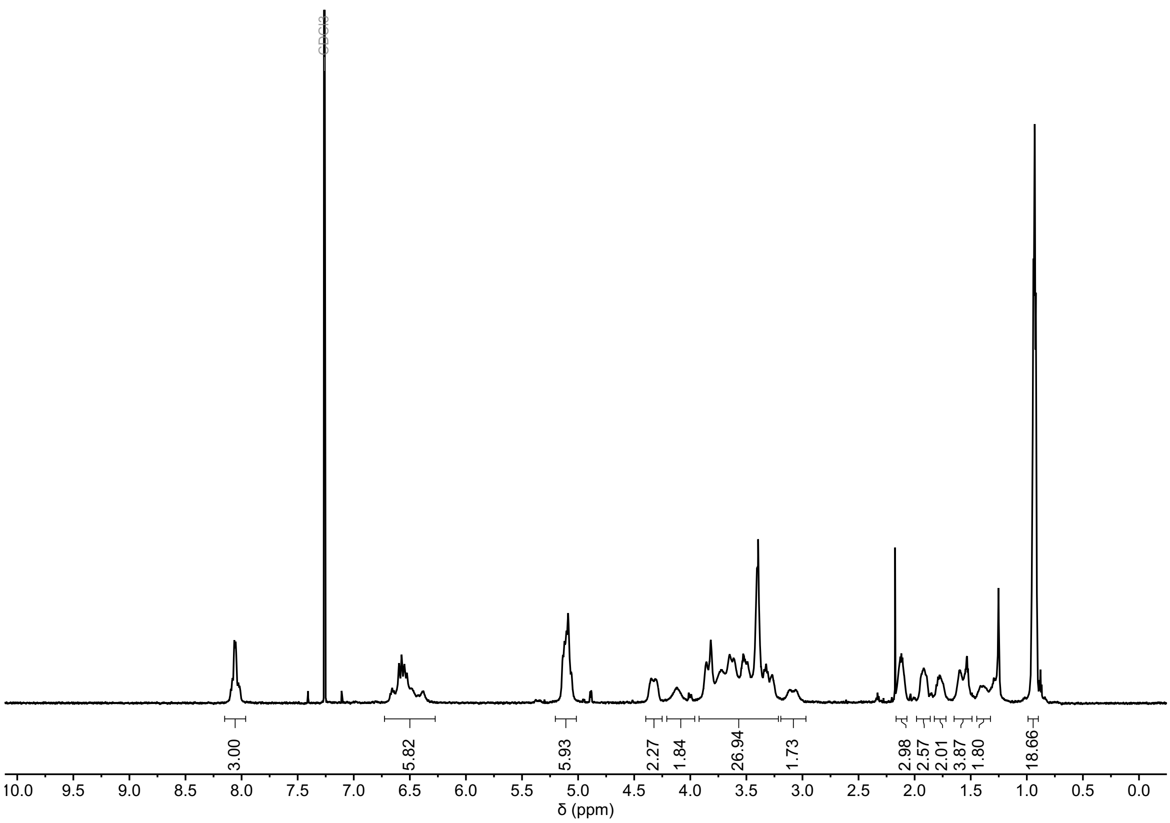

Figure S36. ^{1}H NMR (700 MHz, $CDCl_3$) of compound **17**.

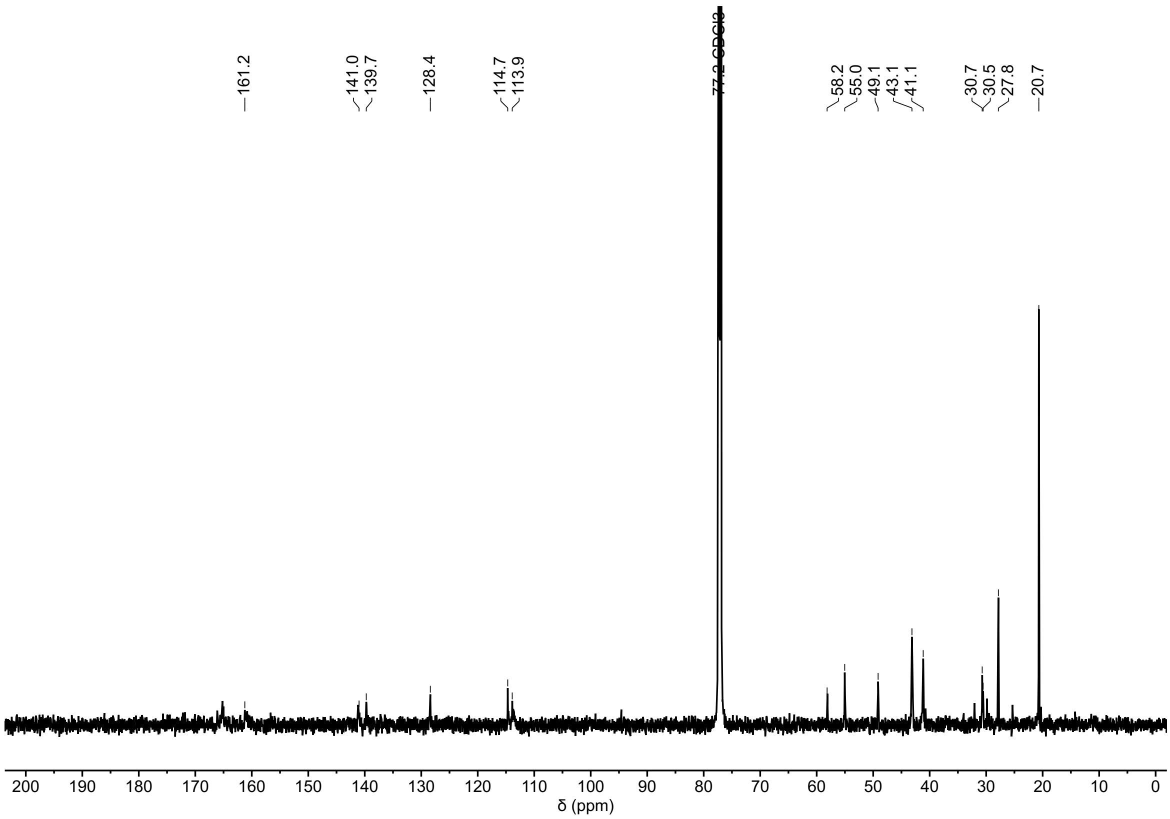

Figure S37. ^{13}C NMR (176 MHz, $CDCl_3$) of compound **17**.

2.6 Synthesis and characterization of 4-nitropehnol 3-mer 18

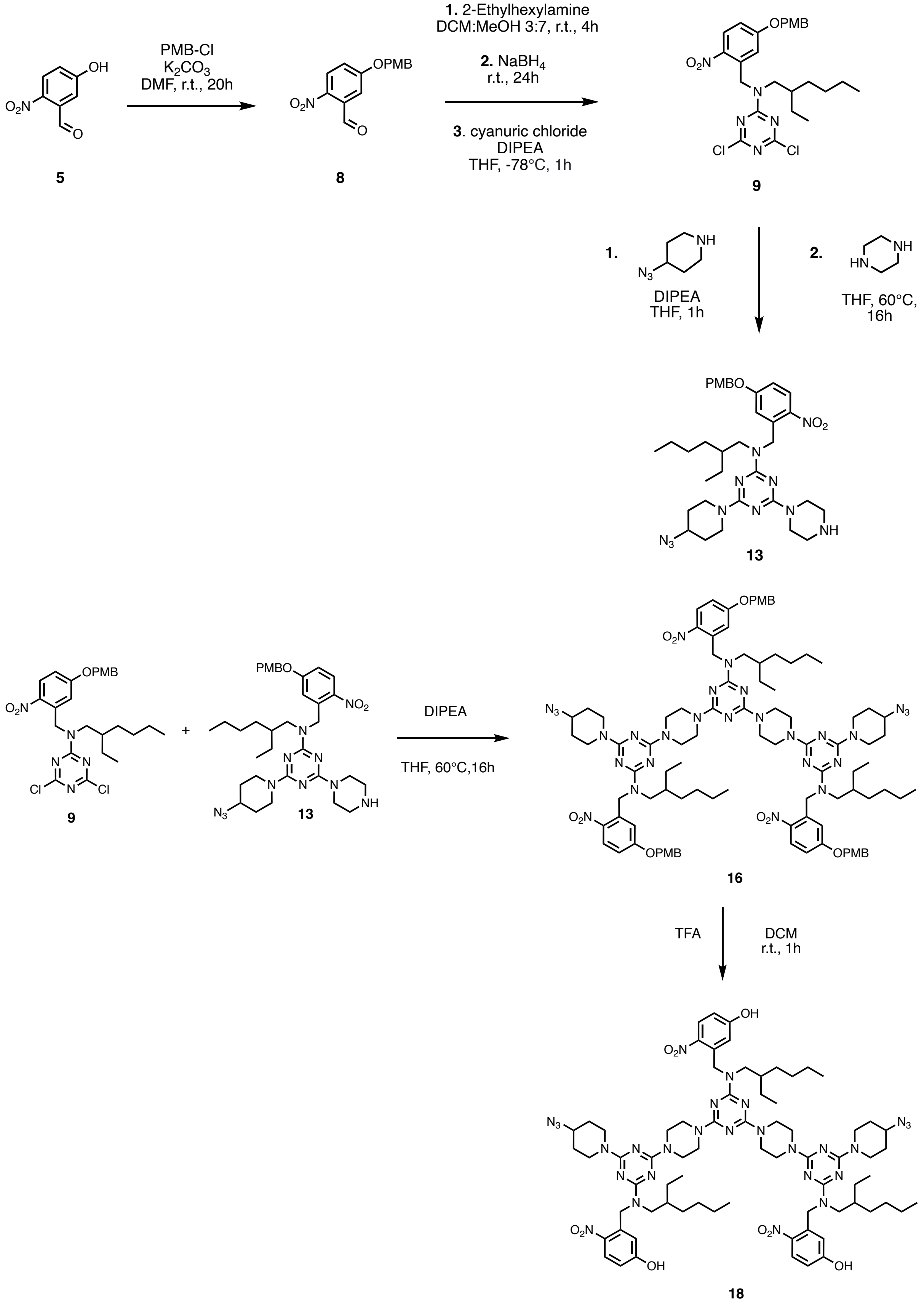

Scheme S6. Synthesis of compound **18**.

Synthesis of compound 8

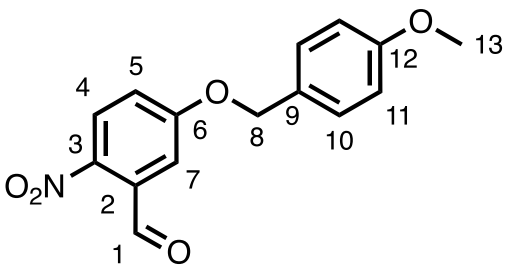

Under an inert N_2 atomosphere, 5-Hydroxy-2-nitrobenzaldehyde (2.75 g, 16.5 mmol) and K_2CO_3 (4.48 g, 32.4 mmol) were dissolved in anhydrous DMF (20 mL). PMB-Cl (4.30 mL, 33.7 mmol) was added dropwise, and the resulting orange slurry was stirred at room temperature for 20h. After that, the crude was diluted with water (150 mL) and extracted with EtOAc (3 × 20 mL). The combined organic layers were washed with brine (20 mL), dried ($MgSO_4$), filtered and concentrated, obtaining an orange/yellow solid. The crude was purified by recrystallization from EtOAc affording the product as a yellow solid (4.05 g, 85%).

^{1}H NMR (500 MHz, $CDCl_3$) δ 10.47 (s, 1H, C1H), 8.14 (d, *J* = 9.0 Hz, 1H, C4H), 7.40 (d, *J* = 2.9 Hz, 1H, C7H), 7.38 – 7.32 (m, 2H, C10H), 7.19 (dd, *J* = 9.0, 2.9 Hz, 1H, C5H), 6.96 – 6.91 (m, 2H, C11H), 5.13 (s, 2H, C8H_2), 3.82 (s, 3H, C13H_3).

^{13}C NMR (126 MHz, $CDCl_3$) δ 188.6 (C1), 163.3 (C6), 160.1 (C12), 142.4 (C3), 134.4 (C2), 129.6 (C10), 127.4 (C4), 127.0 (C9), 119.5 (C5), 114.4 (C11), 114.3 (C7), 71.1 (C8), 55.4 (C13).

HRMS: (ESI^-) calculated for $[C_{15}H_{12}NO_5]^-$: 286.0721, found $[M-H]^-$: 286.0724.

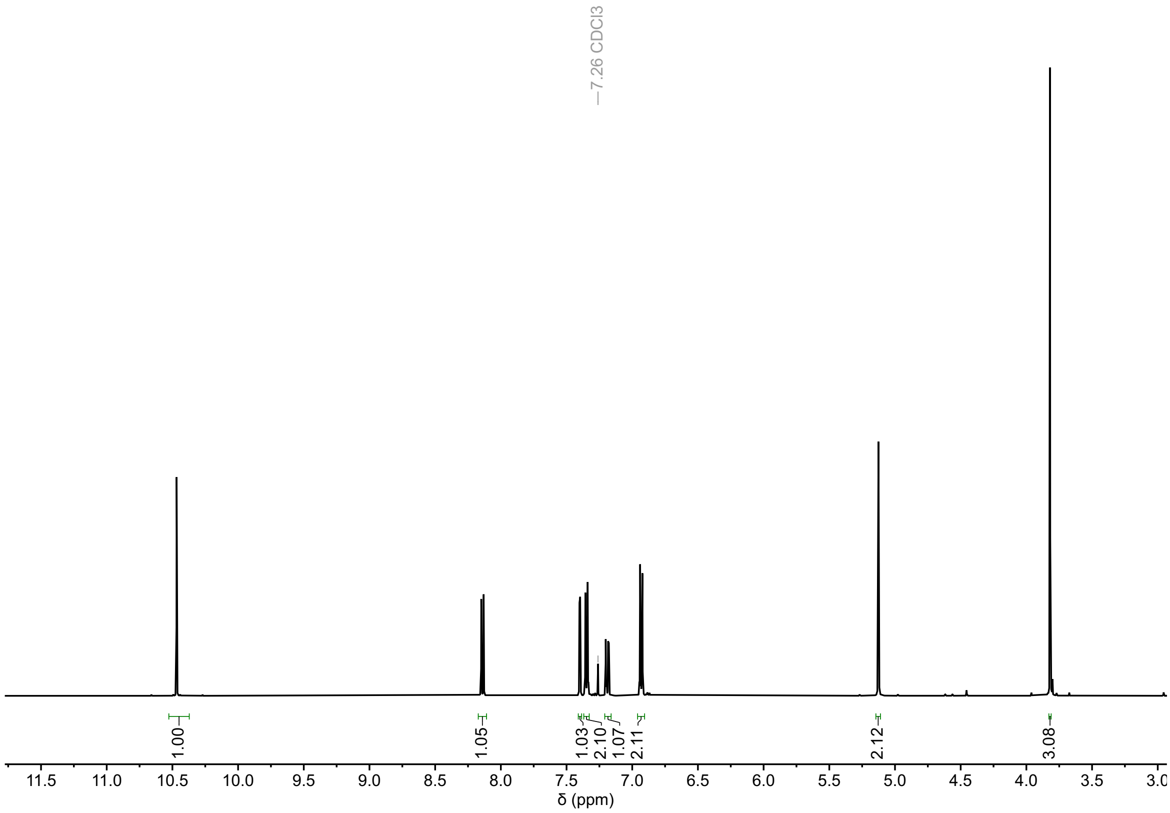

Figure S38. ^{1}H NMR (500 MHz, $CDCl_3$) of compound **8**.

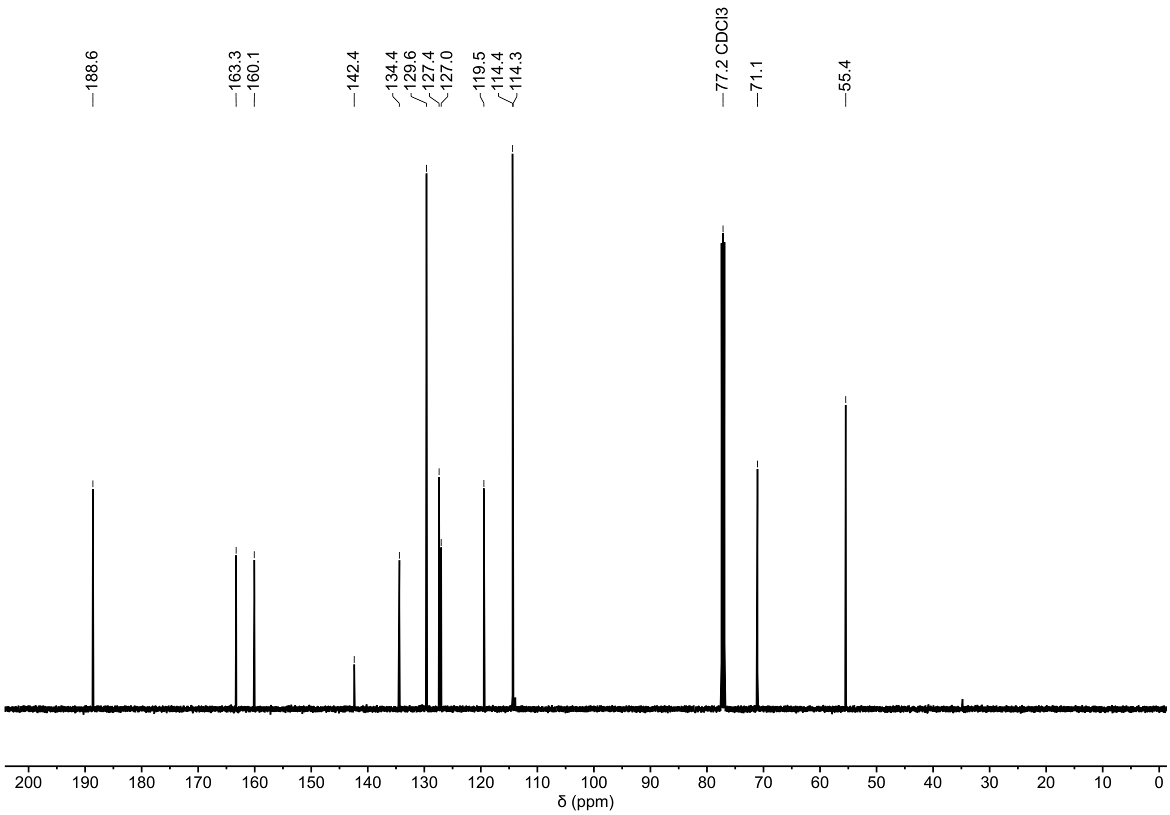

Figure S39. ^{13}C NMR (126 MHz, $CDCl_3$) of compound **8**.

Synthesis of compound 9

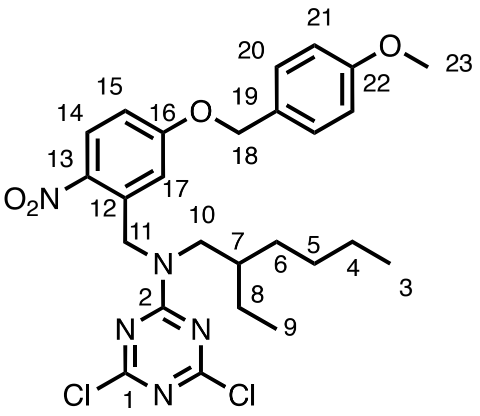

Under an inert N_2 atmosphere, compound **8** (4.04 g, 14.0 mmol) was dissolved in 7:3 MeOH:CH_2Cl_2 (50 mL) in the presence of molecular sieves (4 Å). 2-Ethylhexylamine (4.10 mL, 25.0 mmol) was added, and the reaction was stirred at room temperature for 4h. $NaBH_4$ (0.84 g, 22.0 mmol) was added slowly, and the reaction mixture was stirred at room temperature for 24h. The reaction was then quenched by addition of aq. NaOH (1M, 100 mL). The crude was extracted with EtOAc (3 x 50 mL), and the combined organic layers were washed with aq. LiCl (100 mL), dried ($MgSO_4$), filtered and concentrated under reduced pressure, obtaining a yellow oil. The obtained oil was dissolved in anhydrous THF (15 mL) and the resulting solution was added dropwise to a solution of cyanuric chloride (4.2 g, 23.0 mmol) in anhydrous THF (45 mL) at -78°C. DIPEA (6.30 mL, 30.0 mmol) was then added dropwise. The reaction mixture was stirred at -78°C for 1h. After that, the crude was diluted with EtOAc (40 mL) and the organic layer was washed with water (2 × 80 mL) and aq. LiCl (40 mL). The crude was further extracted with EtOAc (2 × 40 mL). The combined organic layers were dried over $MgSO_4$, filtered and concentrated under reduced pressure. The crude residue was purified by flash column chromatography (silica gel, 0-30% EtOAc in petroleum ether 40-60°C) affording the product as a pale yellow crystalline solid (5.81 g, 10.6 mmol, 76% yield over three steps).

^{1}H NMR (500 MHz, $CDCl_3$) δ 8.23 (d, *J* = 9.00 Hz, 1H, C14$\underline{H}$), 7.23 (dm, *J* = 8.3 Hz, 2H, C20$\underline{H}$), 6.97 (dd, *J* = 9.1 Hz, 2.7 Hz, 1H, C15$\underline{H}$), 6.89 (m, 2H, C21$\underline{H}$), 6.50 (d, *J* = 2.7 Hz, 1H, C17$\underline{H}$), 5.22 (m, 2H, C11$\underline{H}_2$), 5.04 (ABq, J_{AB} = 17.6 Hz, $\Delta\delta_{AB}$ = 0.04, 2H, C18$\underline{H}_2$), 3.82 (s, 3H, C23$\underline{H}_3$), 3.44 (d, *J* = 7.4 Hz, 2H, C10$\underline{H}_2$), 1.79 (sept, *J* = 6.3 Hz, 1H, C7$\underline{H}$), 1.35-1.16 (m, 8H, C4$\underline{H}_2$-C5$\underline{H}_2$-C6$\underline{H}_2$-C8$\underline{H}_2$), 0.91-0.82 (m, 6H, C3$\underline{H}_3$-C9$\underline{H}_3$).
^{13}C NMR (176 MHz, $CDCl_3$) δ 170.5 (C1), 170.3 (C1'), 165.97 (C2), 163.3 (C16), 160.1 (C22), 141.1 (C13), 135.1 (C12), 129.1 (C20), 128.9 (C14), 127.2 (C19), 114.4 (C21), 113.8 (C17), 113.5 (C15), 70.7 (C18), 55.4 (C23), 51.7 (C10), 49.3 (C11), 37.5 (C7), 30.3 (C6 or C8), 28.4 (C6 or C8), 23.7 (C5), 23.1 (C4), 14.1 (C3 or C9), 10.6 (C3 or C9).
HRMS: (ESI^+) calculated for $[C_{26}H_{32}Cl_2N_5O_4]^+$: 548.1826, found $[M+H]^+$: 548.1828.

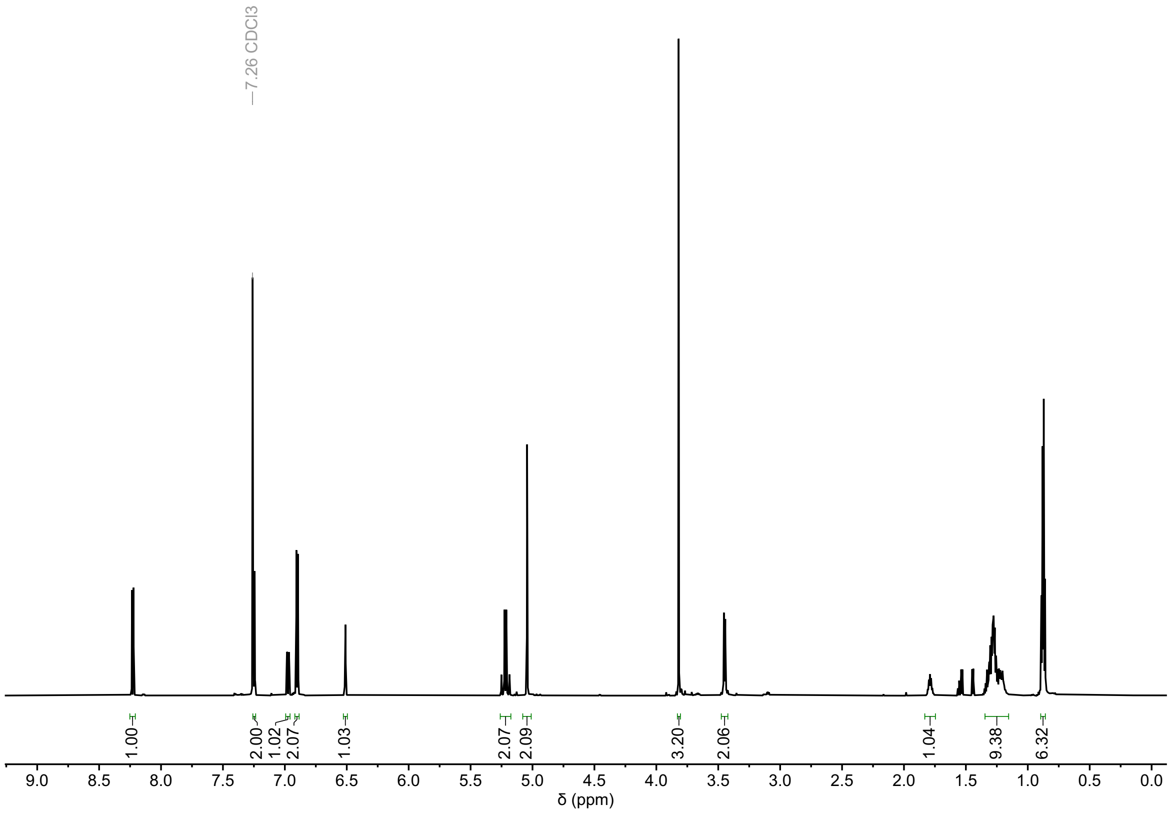

Figure S40. ^{1}H NMR (500 MHz, $CDCl_3$) of compound **9**.

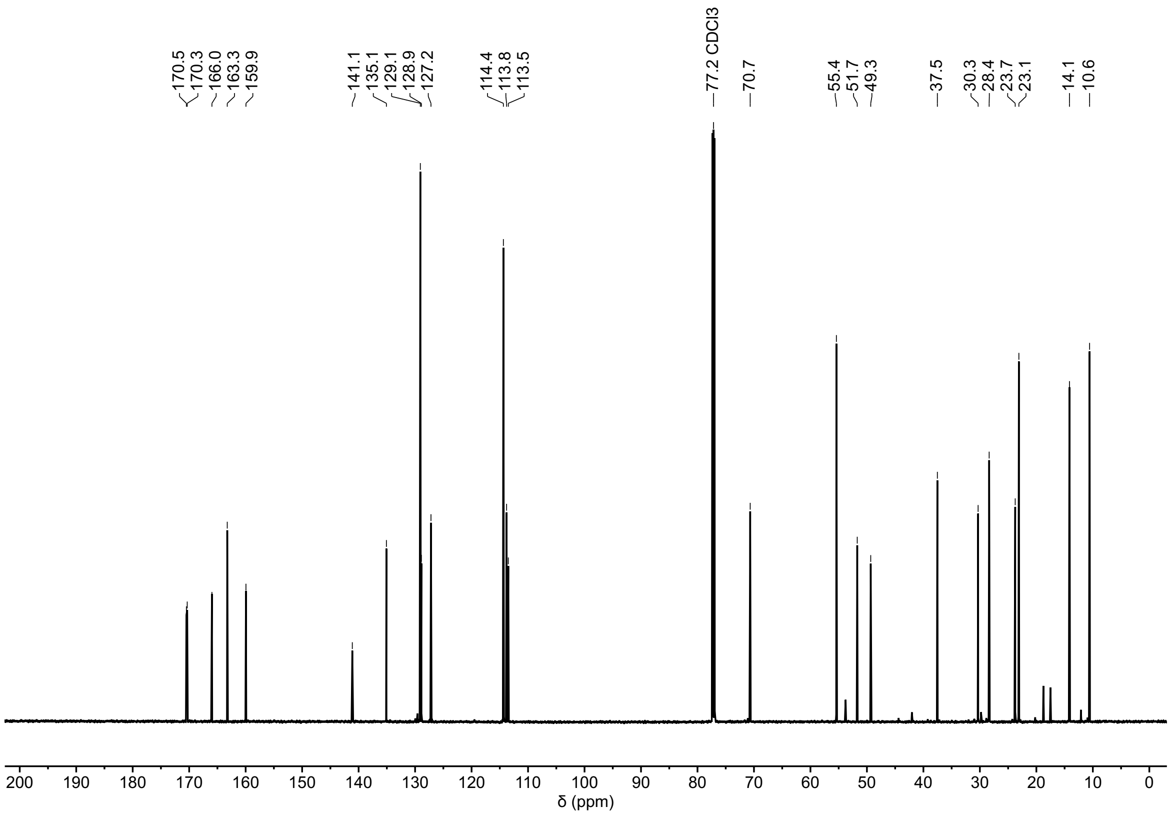

Figure S41. ^{13}C NMR (176 MHz, $CDCl_3$) of compound **9**.

Synthesis of compound 13

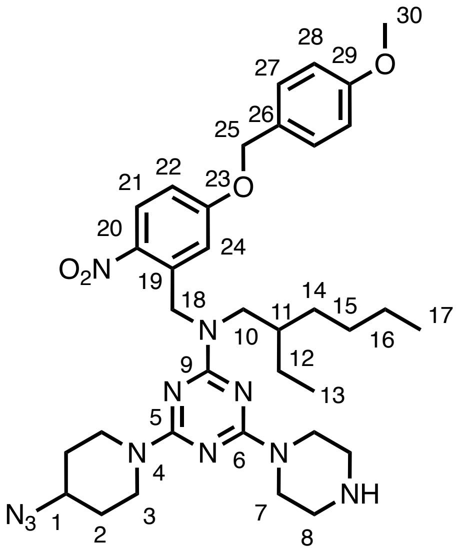

A solution of 4-azidopiperidine (TFA salt) (430 mg, 1.8 mmol) in THF (0.8 mL) was added dropwise to a solution of **9** (1 g, 1.8 mmol) in THF (10 mL) at 0°C. This was followed by addition of DIPEA (0.940 mL, 5.4 mmol) at 0°C. The solution was stirred for 1h at room temperature and monitored by HPLC. A suspension of piperazine (1.56 g, 18 mmol) in THF (15 mL) was added to the reaction mixture at 0°C. The resulting suspension was warmed to room temperature and stirred at 60°C for 16h. EtOAc (60 mL) was added to the solution and the organic layer was washed with H_2O (3 x 40 mL) and dried over $MgSO_4$. The solvent was removed under reduced pressure to afford compound **13** (1.05 g, 85%). The NMR spectra are consistent with the presence of slowly exchanging rotamers in solution.

^{1}H NMR (700 MHz, $CDCl_3$) δ 8.10 (d, *J* = 9.1 Hz, 1H, C21H), 7.30 – 7.26 (m, 2H, C27H), 6.91 – 6.83 (m, 2H, C28H), 6.86 (dd, *J* = 9.2, 2.8 Hz, 1H, C22H), 6.75 (d, *J* = 2.8 Hz, 1H, C24H), 5.12 – 5.02 (m, 2H, C18H_2), 4.97 (s, 2H, C25H_2), 4.38 (s, 1H, C3H),4.32-3.95 (br, 1H, C3H), 3.80 (s, 3H, C30H_3), 3.75-3.71 (m, 2H, CH_2-N piperidine), 3.66-3.60 (m, 1H, C1H), 3.53-3.46 (m, 1H, C1H), 3.46-3.35 (m, 2H, C10H_2), 3.29 (m, 1H, C3H), 3.15-2.95 (br, 1H, C3H) 2.89 (m, 2H, CH_2-N piperazine), 2.74 (m, 2H, CH_2-N piperazine), 1.96-1.89 (m, 1H, C2H), 1.82-1.67 (m, 1H, C11H), 1.63-1.53 (m, 1H, C2H), 1.42 – 1.19 (m, 10H, C16H_2-C15H_2-C14H_2-C12H_2- C2H), 0.86 (m, 6H, C13H_3-C17H_3).

^{13}C NMR (176 MHz, $CDCl_3$) δ 166.2 (C=N), 166.2 (C=N), 165.5 (C=N), 165.3 (C=N), 165.2 (C=N), 165.1 (C=N), 163.0 (C23), 159.9 (C29), 141.4 (C20), 141.4, 139.4 (C19), 139.4, 129.6 (C27), 127.8 (C21), 127.6 (C26), 114.4 (C24), 114.3 (C28), 112.6 (C22), 112.6, 70.4 (C25), 58.4 (C1), 58.3 (C1), 55.3 (C30), 51.2 (C10), 51.1 (C10), 49.0 (C18), 48.9 (C18), 46.2 (C7 or C8), 46.0 (C7 or C8), 44.5 (C7 or C8), 44.3 (C7 or C8, 41.0 (C3), 38.3 (C11), 30.8-30.7 (C2), 30.5 (C14 or C12), 28.9 (C14 or C12), 24.0 (C15), 23.3 (C16), 14.2 (C13 or C17), 10.8 (C13 or C17).

HRMS: (ESI^+) calculated for $[C_{35}H_{50}N_{11}O_4]^+$: 689.4125, found $[M+H]^+$: 689.4106.

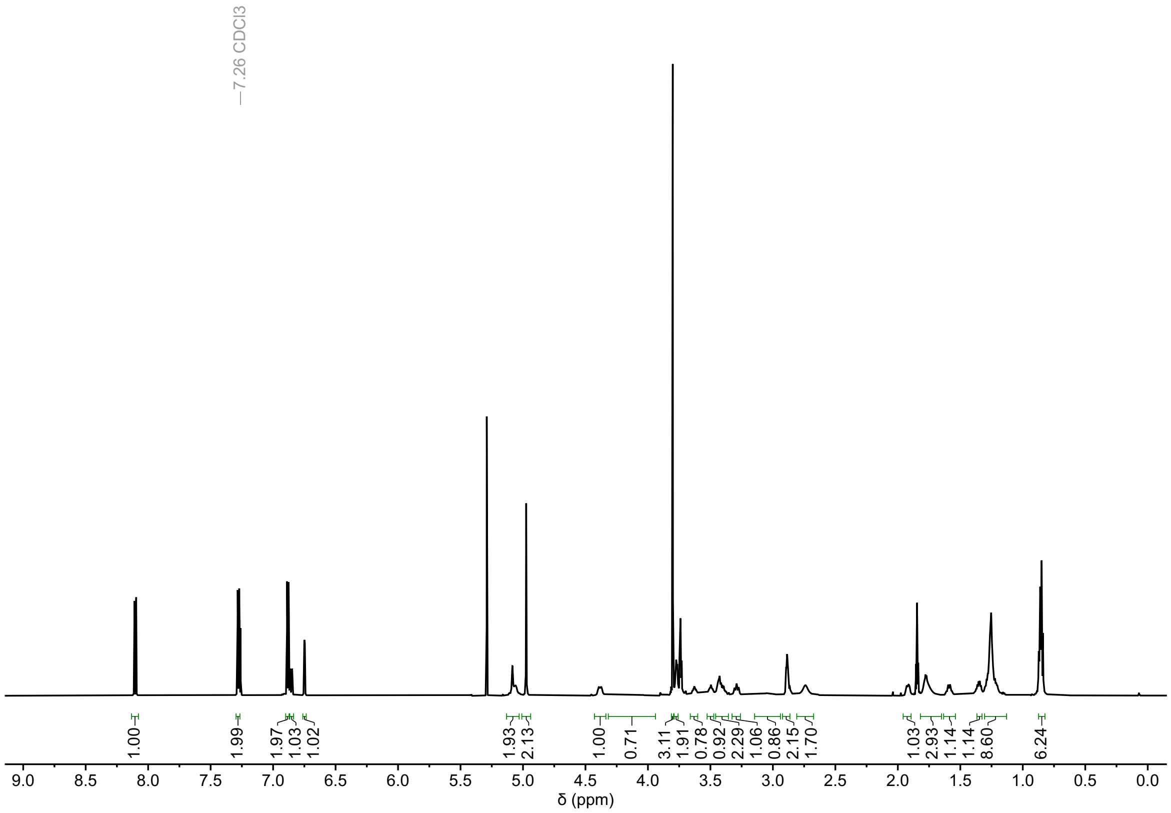

Figure S42. ^{1}H NMR (700 MHz, $CDCl_3$) of compound **13**.

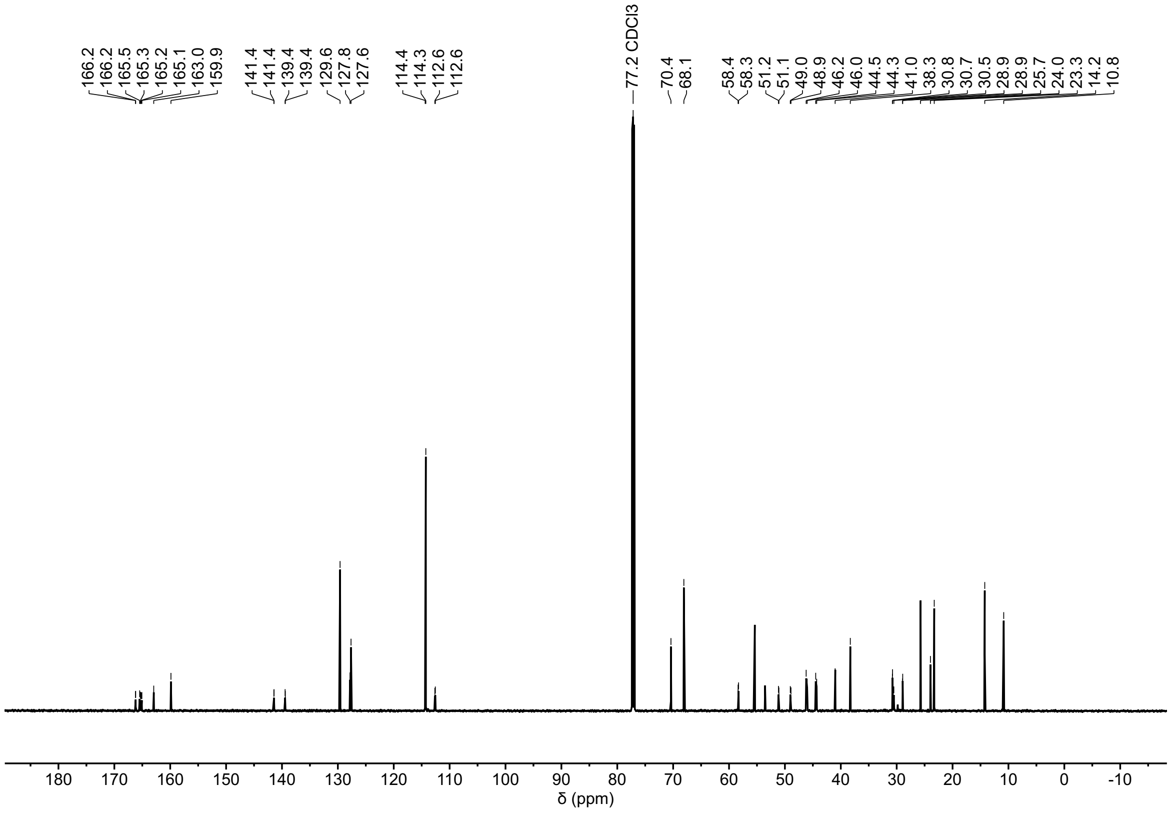

Figure S43. ^{13}C NMR (176 MHz, $CDCl_3$) of compound **13**.

Synthesis of compound 16

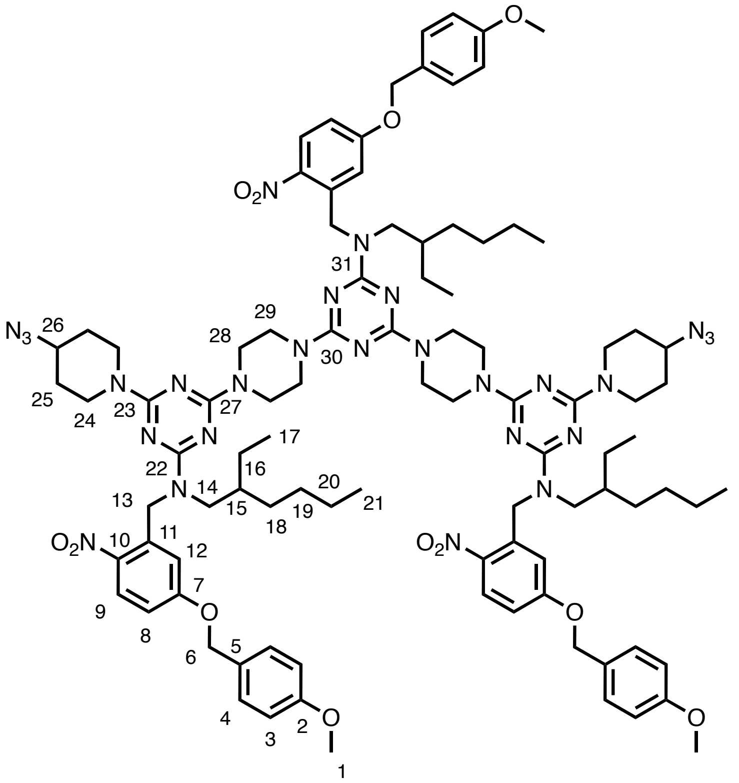

DIPEA (28 μL, 159 μmmol) was added to a solution of **9** (87 mg, 159 μmmol) and **13** (500 mg, 730 μmmol) in THF. The solution was stirred at 60°C for 16h. EtOAc (30 mL) was added to the reaction mixture and the organic layer was washed with H_2O (3 x 20 mL) and dried with $MgSO_4$. The crude compound was purified by column chromatography (0% to 100% EtOAc in petroleum ether 40-60 and then 0% to 10% MeOH in EtOAc to recover the excess of starting material) and the solvents were removed under reduced pressure to afford compound **16** (170 mg, 58%). The NMR spectra are consistent with the presence of slowly exchanging rotamers in solution.

^{1}H NMR (700 MHz, $CDCl_3$) δ 8.19-8.09 (m, 3H, C9H), 7.29-7.22 (under solvent peak C4H), 6.90-6.82 (m, 9H, C3H-C8H), 6.74 (m, 3H, C12H), 5.16-5.03 (m, 6H, C13H_2), 5.03-4.88 (m, 6H, C6H_2), 4.46-4.33 (m, 2H, C24H), 3.91-3.81 (m, 4H, CH_2 piperidine), 3.81-3.73 (m, 12H, C1H_3-CH piperidine), 3.69-3.57 (m, 4H, C26H-CH piperidine), 3.54-3.35 (m, 10H, C26H-C14H_2-CH piperidine), 3.35-3.25 (m, 2H, C24H), 1.97-1.88 (m, 2H, C25H), 1.84-1.71 (m, 5H, C15H-C25H), 1.66-1.55 (m, 2H, C25H), 1.42-1.14 (m, 26H, C25H-C16H_2-C18H_2-C19H_2-C20H_2), 0.94-0.77 (m, 18H, C17H_3-C21H_3).

^{13}C NMR (176 MHz, $CDCl_3$) δ 166.2 (C=N), 165.4 (C=N), 163.02(C7), 163.0 (C7), 159.9 (C2), 141.4 (C10), 139.3 (C11), 129.6 (C4), 128.0 (C5), 127.9 (C9), 127.6, 114.4 (C12), 114.3 (C3), 112.6 (C8), 70.4 (C6), 58.4 (C26), 58.3 (C26), 55.4 (C1), 51.1 (C14), 49.0 (C13), 43.3 (C-N), 43.1 (C-N), 43.0 (C-N), 41.1 (C24), 38.3 (C15), 30.8 (C25), 30.6 (C16), 29.0 (C18), 24.0 (C19), 23.3 (C20), 14.3 (C17 or C21), 10.9 (C17 or C21).

HRMS: (ESI^+) calculated for $[C_{96}H_{128}N_{27}O_{12}]^+$: 1852.0314, found $[M+H]^+$: 1852.0350.

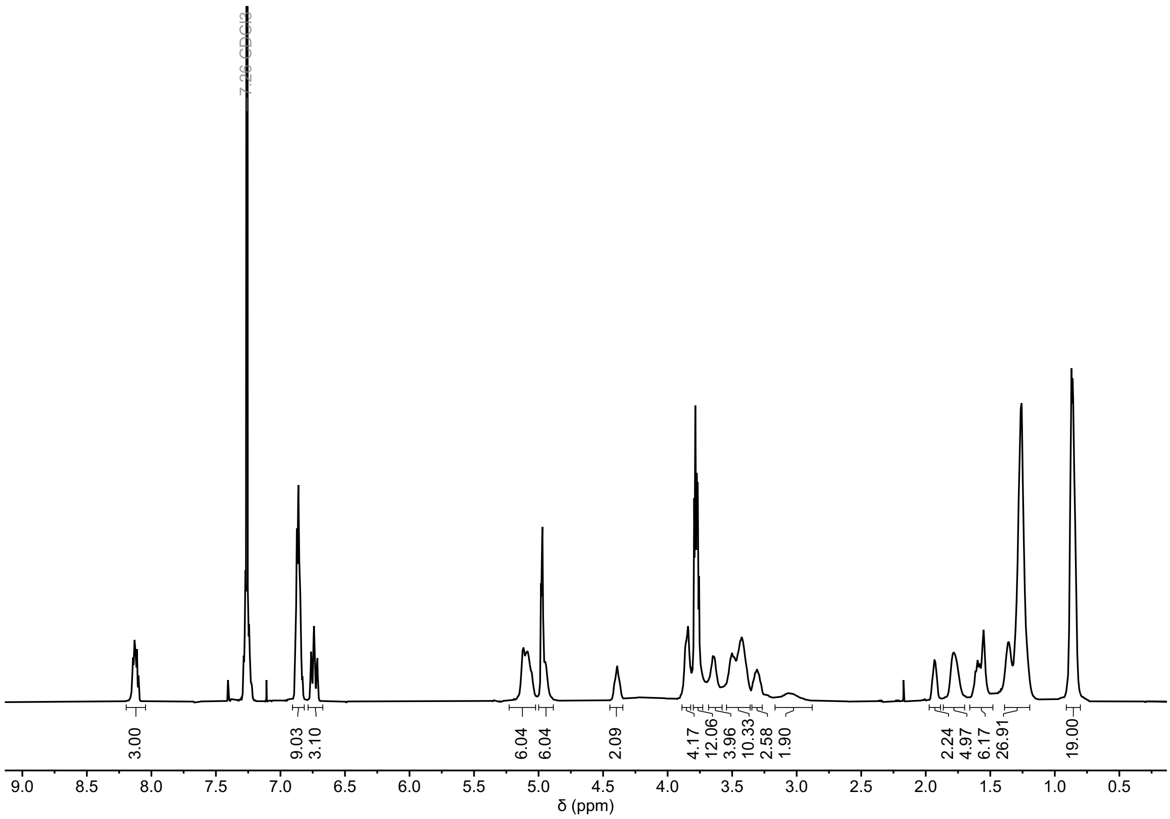

Figure S44. ^1H NMR (700 MHz, $CDCl_3$) of compound **16**.

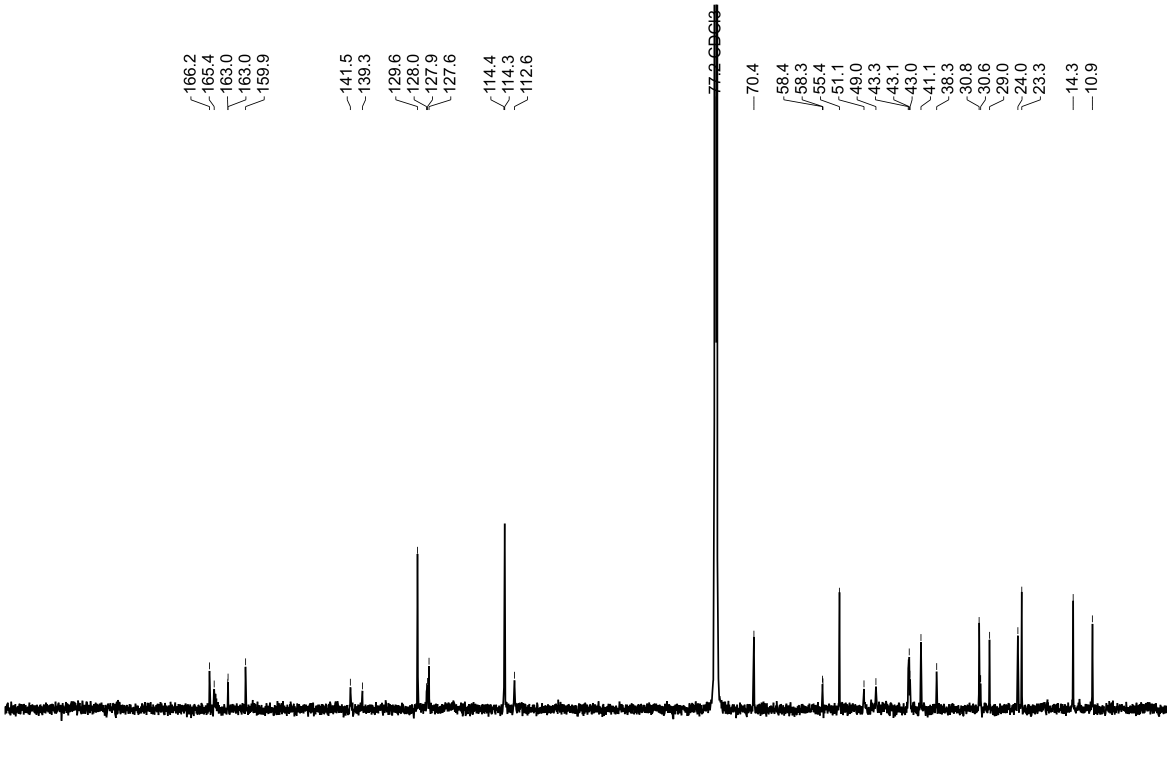

Figure S45. ^{13}C NMR (176 MHz, $CDCl_3$) of compound **16**.

Synthesis of compound 18

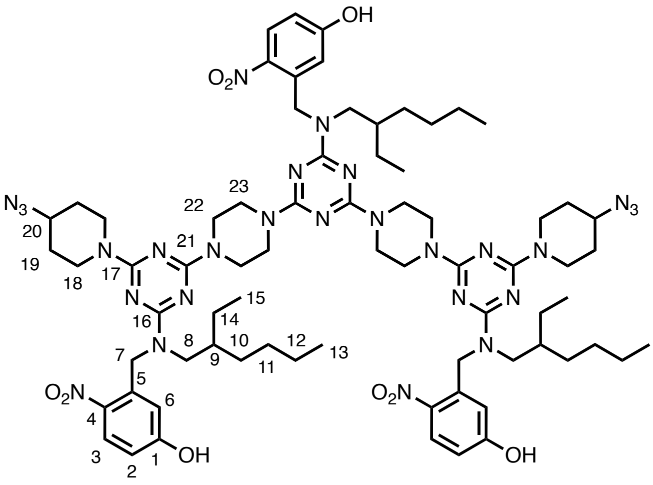

TFA (3.5 mL) was added to a solution of **16** (70 mg, 37.8 μmol) in DCM (3.5 mL) at 0°C. The solution was stirred at room temperature for 1 hour. The reaction mixture was flushed with N_2, and the resulting residue was dissolved in DCM (20 m), washed with H_2O (2 x 15 mL), dried over $MgSO_4$ and the solvent was removed under reduced pressure. The crude compound was purified by flash chromatography (SiO_2, 0-15 % MeOH in DCM) to afford compound **18** (51 mg, 90%) as a yellow oil. The NMR spectra are consistent with the presence of slowly exchanging rotamers in solution.

^{1}H NMR (700 MHz, $CDCl_3$) δ 8.22-7.93 (m, 3H, C3H), 6.73-6.22 (m, 6H, C2H-C6H), 5.32-4.81 (m, 6H, C7H_2), 4.42-4.23 (m, 2H, C18H), 3.98-2.95 (m, 28H, C18H-C22H_2-C23H_2-C8H_2), 1.97-1.87 (m, 2H, C19H), 1.85-1.71 (m, 5H, C9H-C19H), 1.69-1.46 (m, 2H, C19H), 1.46-1.56 (m, 26H, C10H_2- C11H_2-C12H_2-C14H_2-C19H), 0.91-0.82 (m, 18H, C15H_3-C13H_3).

^{13}C NMR (176 MHz, $CDCl_3$) δ 166.3-164.7 (C=N), 141.3, 139.7, 128.4 (C3), 114.6 (C2 or C6), 113.9 (C2 or C6), 58.1 (C20), 51.3 (C8), 49.1 (C7), 43.1 (C22-C23), 41.2 (C18), 38.4 (C9), 30.8-30.5 (C19), 29.8 (C10 or C14), 29.0 (C10 or C14), 24.0 (C11), 23.3 (C12), 14.3 (C13 or C15), 10.9 (C13 or C15).

HRMS: (ESI^+) calculated for $[C_{72}H_{104}N_{27}O_9]^+$: 1490.8505, found $[M+H]^+$: 1490.8545.

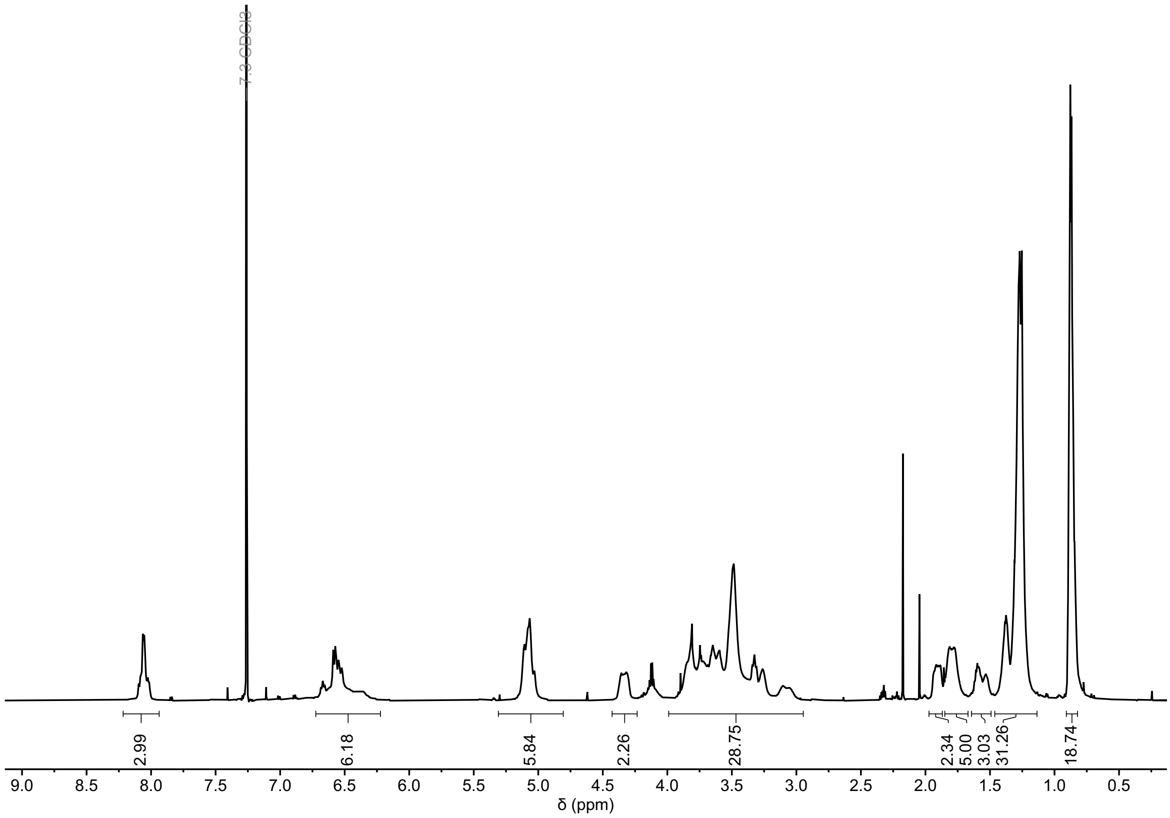

Figure S46. ^{1}H NMR (700 MHz, $CDCl_3$) of compound **18**.

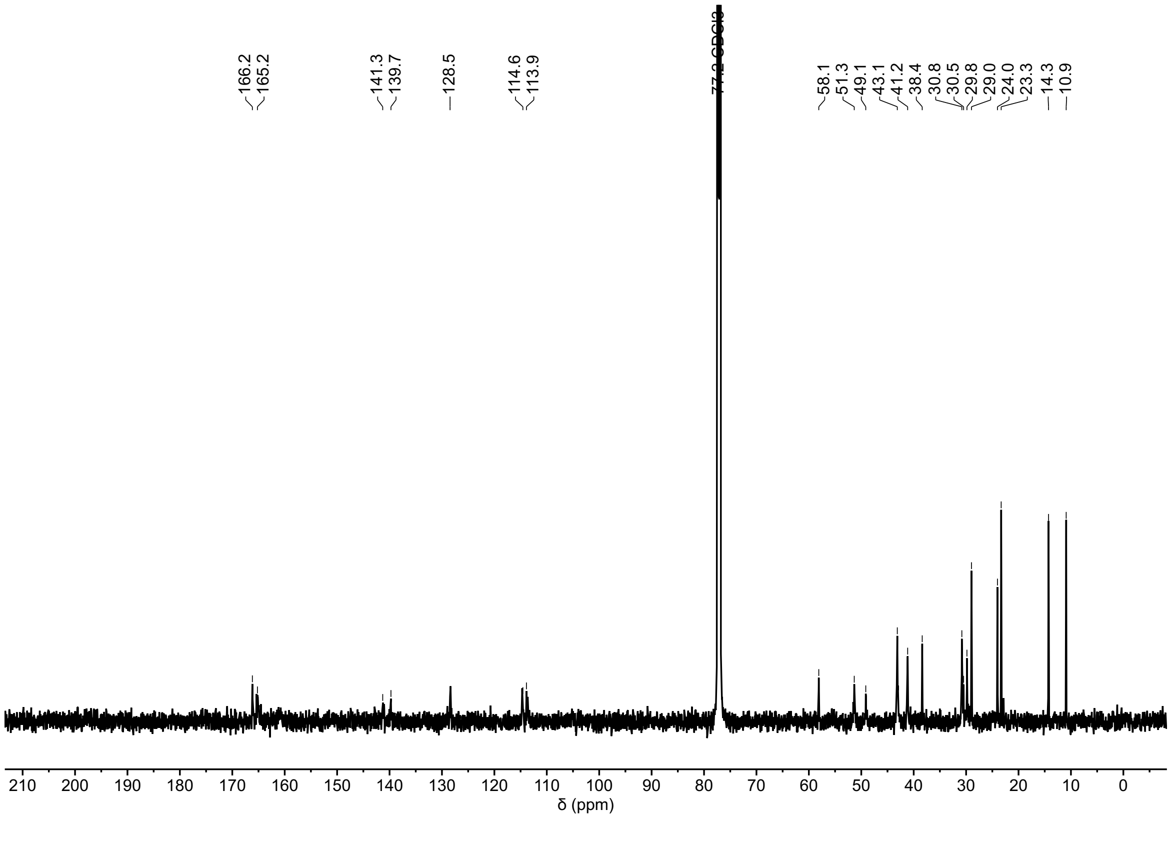

Figure S47. ^{13}C NMR (176 MHz, $CDCl_3$) of compound **18**.

3. Template experiments

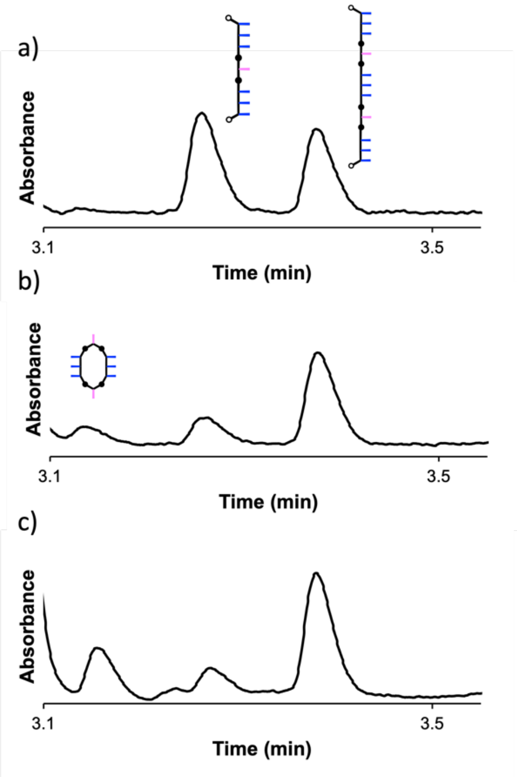

Figure S48. Partial UPLC traces of the crude reaction mixtures obtained in the template reaction using the 11-mer template **pAAA-C-AAA-C-AAAp** 0.005 mM, **zDDDz** 0.09 mM and various amount of **zAAAz**: a) 0.045 mM, b) 0.09 mM, c), d) 0.18 mM. Conditions: 40 µM [Cu(CH_3CN)$_4$]PF_6, 40 µM TBTA, 48h, r.t., DCM. UPLC conditions: C4 at 40°C using gradient of 30–100% of THF/formic acid (0.1%) in water/formic acid (0.1%) over 4 min, then 100% THF/formic acid (0.1%) over 2 min. The UV-vis absorbance at 254 nm is plotted.

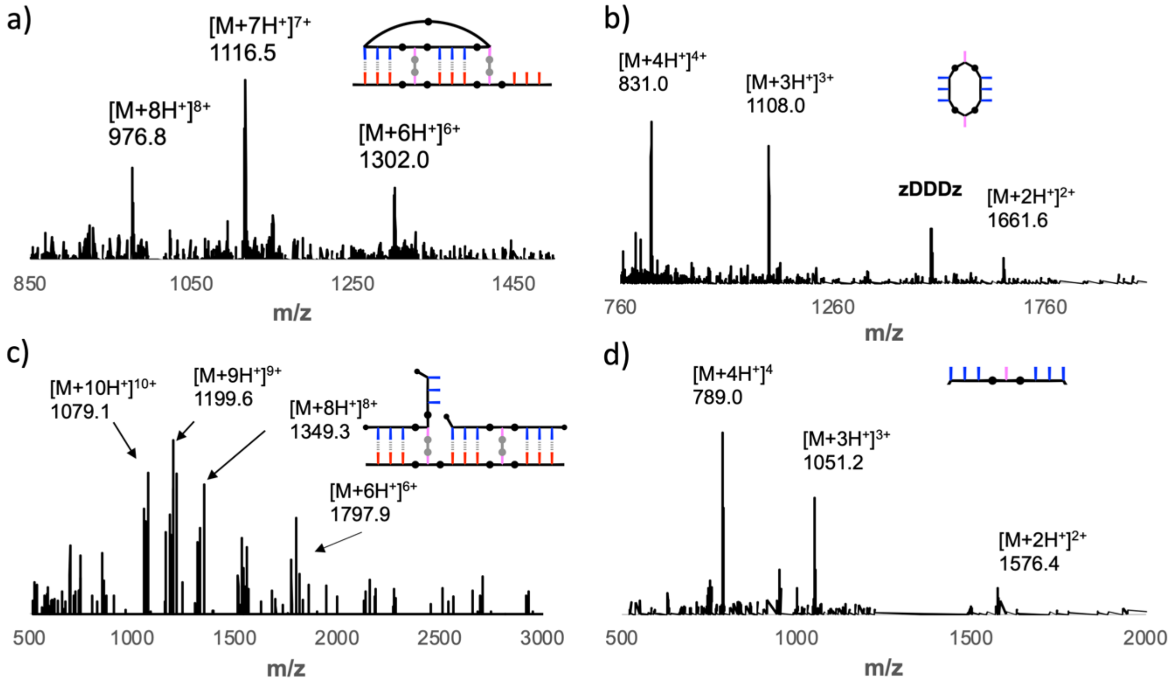

Figure S49. Side products formed using the 11-mer template. a) ESI-MS of the UPLC peak due to the macrocyclic side product formed in the ZIP step (labelled with an asterisk in Figure 8d of the main text). Calculated mass (ESI^+): 1302.0 $[M+6H]^{6+}$, 1116.1 $[M+7H]^{7+}$, 976.7 $[M+8H]^{8+}$. b) ESI-MS of the UPLC peak due to the macrocyclic side product obtained after the cleave step (labelled with an asterisk in Figure 8e of the main text). Calculated mass (ESI^+): 1661.9 $[M+2H]^{2+}$, 1107.9 $[M+3H]^{3+}$, 831.2 $[M+4H]^{4+}$. c) ESI-MS of the UPLC peak due to the second side product formed in the ZIP step (labelled with an asterisk in Figure 8d of the main text). Calculated mass (ESI^+): 1798.6 $[M+6H]^{6+}$, 1349.2 $[M+8H]^{8+}$, 1199.4 $[M+9H]^{9+}$, 1079.5 $[M+10H]^{10+}$. d) ESI-MS of the UPLC peak due to the truncated side product obtained after the cleave step (labelled with an asterisk in Figure 8e of the main text). Calculated mass (ESI^+): 1576.4 $[M+2H]^{2+}$, 1051.6 $[M+3H]^{3+}$, 788.7 $[M+4H]^{4+}$.

4. Binding studies

UV-vis titrations were carried out on an Agilent Cary 60 UV-Vis spectrophotometer, using standard titration protocols. A sample of the host (p**D**p or z**DDD**z) was prepared at a known concentration in DCM (spectroscopic grade). The UV-vis spectrum of the free host (2 mL) was recorded. The guest was dissolved (**pAp** or **zAAAz**) in 2 mL of the host solution at a known concentration. Aliquots of the guest solution were successively added to the cuvette, and the UV-vis absorption spectrum was recorded after each addition. The UV-vis absorption spectra were analysed using a purpose-built Python script to fit the changes in the absorption at fixed wavelengths to a 1:1 binding isotherm by optimizing the association constant and absorption of the free and bound host.

pAp was synthesised as previously reported.[3]

Synthesis of pDp

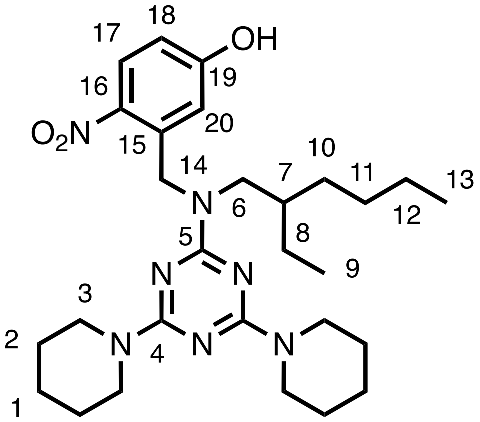

A solution of piperidine (0.22 mL, 2.2 mmol) and DIPEA (0.7 mL, 4 mmol) in THF (2 mL) was added dropwise to a solution of compound **9** (120 mg, 0.219 mmol) in THF (3 mL) at 0°C. The reaction mixture was stirred at room temperature for 1 hour. EtOAc (20 mL) was added to the solution and the organic layer was washed with H_2O (3 x 20 mL) and dried with $MgSO_4$. The solvent was removed *in vacuo* and the residue was dissolved in DCM (2.5 mL). TFA (2.5 ml) was assed dropwise at 0°C and the resulting solution was stirred at room temperature for 1 hour. The reaction mixture was flushed with N_2 and the obtained residue was dissolved in DCM (20 m), washed with H_2O (2 x 15 mL), dried over $MgSO_4$ and the solvent was removed under reduced pressure. The crude compound was purified by flash chromatography (SiO_2, 20-40 % EtOAc in petroleum ether 40-60°C) to afford **pDp** (78 mg, 68%).

^{1}H NMR (700 MHz, $CDCl_3$) δ 8.03 (d, *J* = 8.8 Hz, 1H, C17H), 6.59 (d, *J* = 2.7 Hz, 1H, C20H), 6.57 (dd, *J* = 8.9, 2.7 Hz, 1H, C18H), 5.10 (ABq, J_{AB} = 18.0 Hz, $\Delta\delta_{AB}$ = 0.02, 2H, C14H_2), 3.74 (m, 4H, C3H_2), 3.52 (m, 4H, C3H_2), 3.50 – 3.41 (m, 2H, C6H_2), 1.82 (m, 1H, C7H), 1.68 – 1.62 (m, 2H, C1H_2), 1.60-1.50 (m, 6H, C1H_2-C2H_2), 1.45-1.37 (m, 4H, C2H_2), 1.33 – 1.22 (m, 8H, C8H_2-C10H_2-C11H_2-C12H_2), 0.87 (m, 6H, C9H_3-C13H_3).

^{13}C NMR (176 MHz, $CDCl_3$) δ 166.2 (C5), 165.1 (C4), 161.1 (C19), 141.1 (C15), 140.0 (C16), 128.2 (C17), 114.9 (C20), 113.8 (C18), 51.1 (C6), 48.9 (C14), 44.4 (C3), 44.3 (C3), 38.3 (C7), 30.8 (C8 or C10), 29.0 (C8 or C10), 26.0 (C2), 25.8 (C2), 25.2 (C1), 25.0 (C1), 24.0 (C11), 23.3 (C12), 14.3 (C9 or C13), 10.8 (C9 or C13).

HRMS: (ESI^+) calculated for $[C_{28}H_{43}N_7O_3]^+$: 525.3500, found $[M+H]^+$: 526.3497.

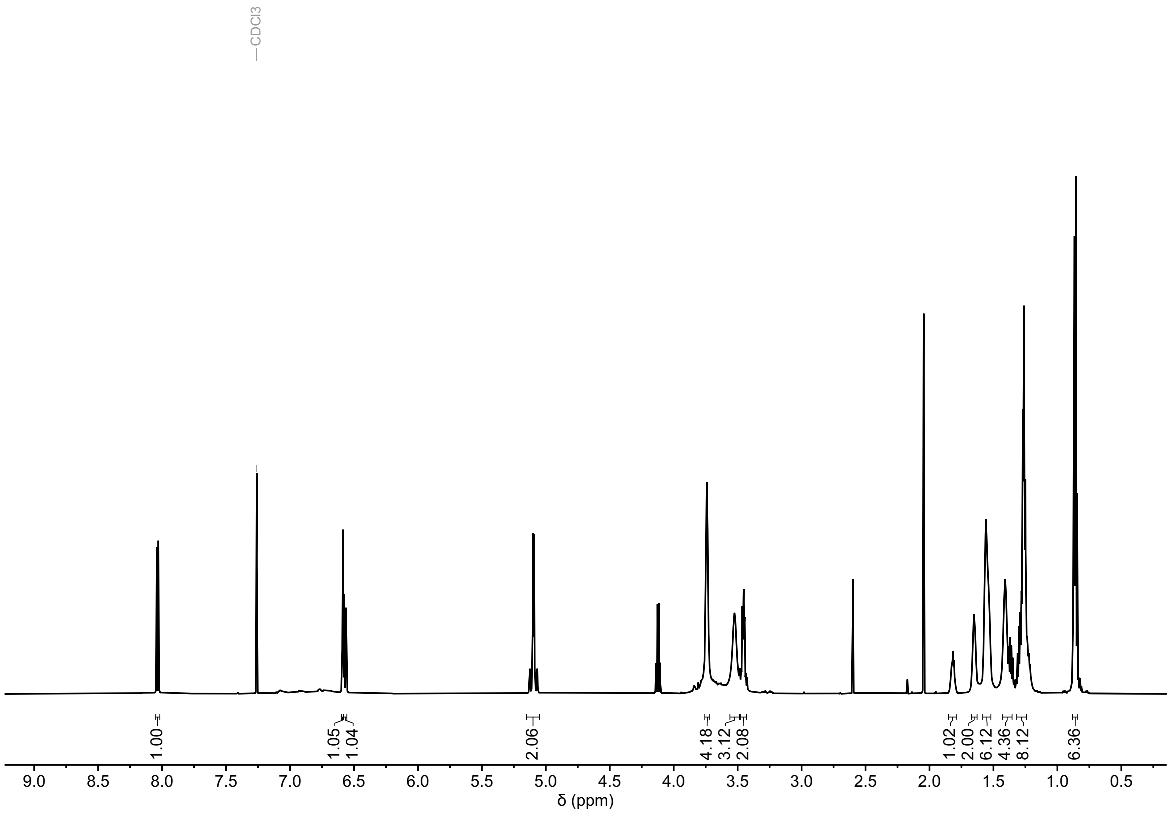

Figure S50. ^{1}H NMR (700 MHz, $CDCl_3$) of **pDp**.

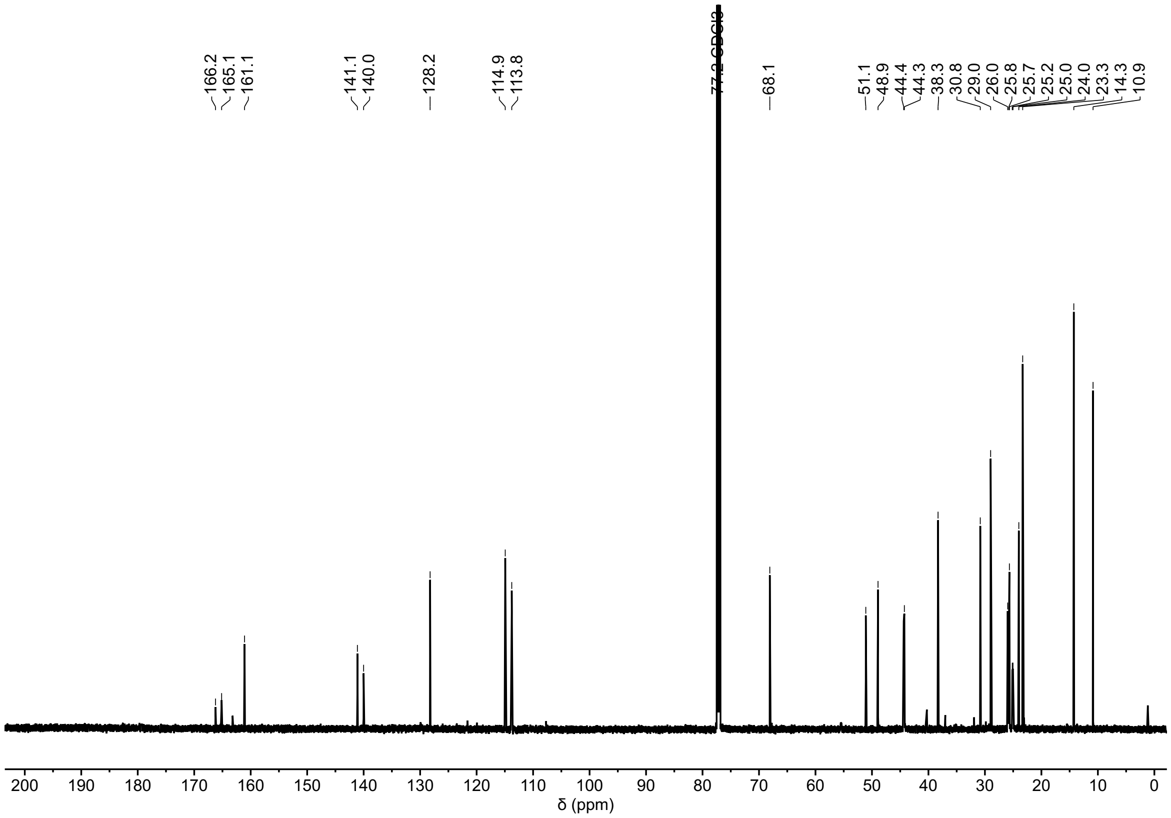

Figure S51. ^{13}C NMR (176 MHz, $CDCl_3$) of compound **pDp**.

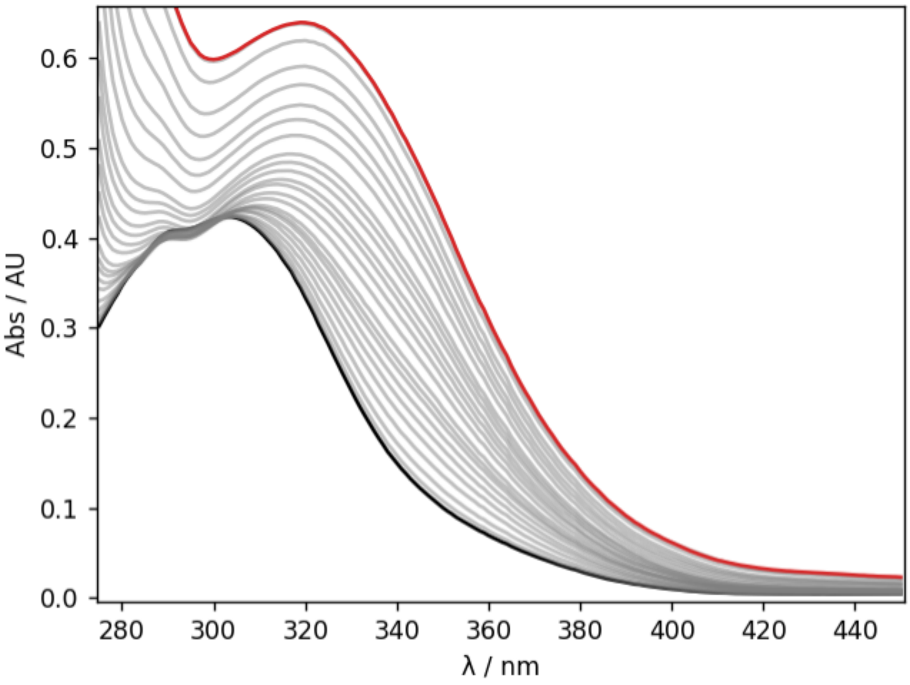

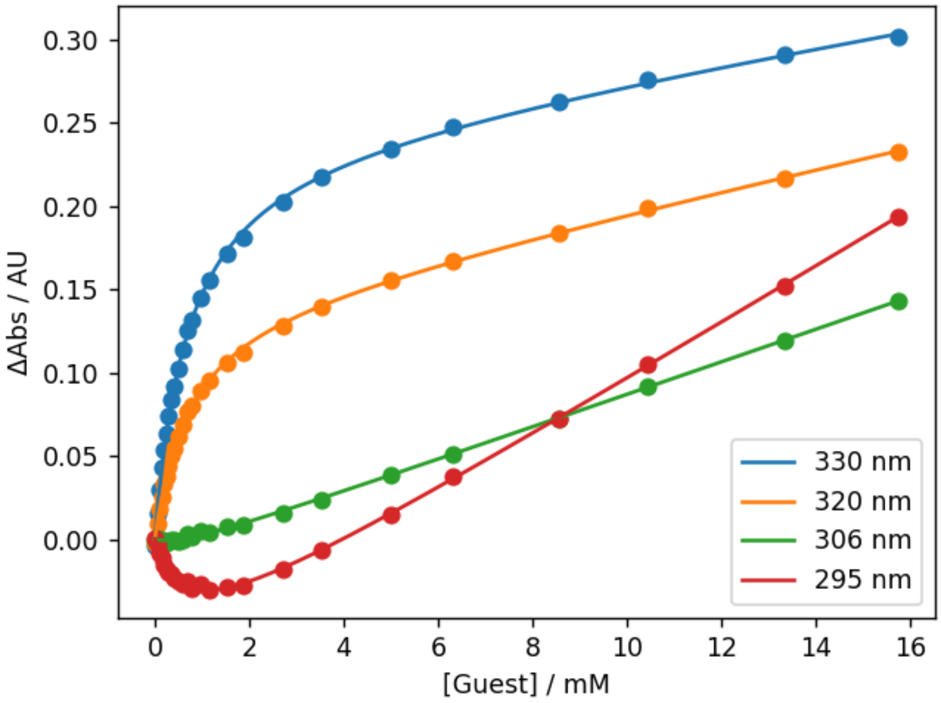

Figure S52. UV-vis absorption titration of **pAp** into **pDp** in dichloromethane at 298 K. On the top, overlay of UV/Vis absorption spectra. The initial spectrum is shown in black, the final spectrum in red. At the bottom, the points are the experimental measurements at selected wavelengths, and the lines are the best fit to a 1:1 binding isotherm that allows for the absorption of the guest (K_a = (1.5±2) x10^3 M^{-1} (average of three titrations).

5. References

[1] Cao S., Sun C., Wang J., Jiang Q., Qiu Y., Wang H., Liao Y., Xie X. *Macromol. Rapid. Commun*., **2022**, *43*, 2200238.

[2] Sikai H., Inoue H., Toba T., Murata K., Narii N., Shimmyo Y., Igawa Y. Matsumoto T., Takemoto N. *Bioorganic & Medicinal Chemistry Letters* **2019**, *29*, 2332-2337.

[3] Troselj P., Bolgar P., Ballester P. and Hunter C.A. *J. Am. Chem. Soc.* **2021**, *143*, 8669−8678.
